# Open optimism as an “embodied-health” ethic for the information era

**DOI:** 10.3389/fphar.2024.1331237

**Published:** 2024-06-17

**Authors:** Meshandren Naidoo

**Affiliations:** School of Law, University of KwaZulu-Natal, Durban, South Africa

**Keywords:** information, freedom, autonomy, artificial intelligence, quantum mechanics, bioethics, non-linear dynamics, health

## Abstract

This article forms part of a series on “openness,” “non-linearity,” and “embodied-health” in the post-physical, informational (virtual) era of society. This is vital given that the threats posed by advances in artificial intelligence call for a holistic, embodied approach. Typically, health is separated into different categories, for example, (psycho)mental health, biological/bodily health, genetic health, environmental health, or reproductive health. However, this separation only serves to undermine health; there can be no separation of health into subgroups (psychosomatics, for example). Embodied health contains no false divisions and relies on “optimism” as the key framing value. Optimism is only achieved through the mechanism/enabling condition of *openness*. Openness is vital to secure the embodied health for individuals *and societies*. Optimism demands that persons become active participants within their own lives and are not mere blank slates, painted in the colors of physical determinism (thus a move away from nihilism—which is the *annihilation* of freedom/autonomy/quality). To build an account of embodied health, the following themes/aims are analyzed, built, and validated: (1) a modern re-interpretation and validation of German idealism (the crux of many legal–ethical systems) and Freud; (2) ascertaining the bounded rationality and conceptual semantics of openness (which underlies thermodynamics, psychosocial relations, individual autonomy, ethics, and as being a central constitutional governmental value for many regulatory systems); (3) the link between openness and societal/individual embodied health, freedom, and autonomy; (4) securing the role of individualism/subjectivity in constituting openness; (5) the vital role of nonlinear dynamics in securing optimism and embodied health; (6) validation of arguments using the methodological scientific value of *invariance* (generalization value) by drawing evidence from (i) information and computer sciences, (ii) quantum theory, and (iii) bio-genetic evolutionary evidence; and (7) a validation and promotion of the inalienable role of *theoretic philosophy* in constituting embodied health, and how modern society denigrates embodied health, by misconstruing and undermining theoretics. Thus, this paper provides and defends an up-to-date non-physical account of embodied health by creating a psycho-physical–biological–computational–philosophical construction. Thus, this paper also brings invaluable coherence to legal and ethical debates on points of technicality from the empirical sciences, demonstrating that each field is saying the same thing.

## 1 Introduction

In 2023, Geoffrey Hinton, better known as “the Godfather of artificial intelligence,” given his role in constructing the regressive function, quit his job at Google, citing the immanent dangers of the recent advances in the field. Hinton’s basic argument is that humanity will end up being slaves to AI, as AI is soon to supersede human intelligence.

Parsimony describes an action, or mode of acting, with the least amount of resource expenditure, which requires the least number of assumptions being made, prior to any act or explanation. In other words, parsimony describes a chosen trajectory or path that is selected based on efficiency. Efficiency thus involves the choosing of a trajectory that involves the least amount of risk, uncertainty, or resistance (as resistance requires more resource expenditure). Hinton understands the concept of parsimony well, given that movements of efficiency are mirrored by the mechanisms of backward propagation/regressive function (below), which are implemented in gradient descent algorithms. Freud’s (now validated) economic theory of mind is based on the notion of energy efficiency. See “Freud” and “Sedimentation and entrenchment” given in Supplementary Material.

To alter Hinton’s claim slightly, Hinton seems to claim that humans and societies are slaves to *intelligence*—which is not merely a “human” phenomenon. Intelligence, in terms of Hinton’s claim, is reflected by the linear *movement of efficiency*. Efficiency is the process of reducing the degrees of freedom and degrees of meta-freedom (Naidoo, 2023a). This movement *creates linearity*. The way in which nihilism arises and is presented in societies is explored in this paper. As an introduction to the concept of nihilism, typically, nihilism arises (in philosophic presentation) because of linear dynamics, in the form of (1) “something” that linearly arises from the *void* of nothingness or (2) something that proceeds in a linear movement toward nothingness, as the *abyss,* or endpoint. The solution to (1) is simply to understand that *quantity* can only exist by being derived from and attached to some *quality*—(no)thing is not nothing (Naidoo, 2023e). Quality is theory itself or subjective rationalizations, hence the fundamental need to protect subjective freedoms and meta-freedoms. The solution to (2) is to secure meta-freedom, which is the ability to construct/define and reconstruct/redefine subjective qualities, as opposed to promoting quantity, as done by the society. Quality can also be understood as the production of creative or critical thought (abstract thinking), whereas quantity can be understood as the production of said quality (in the form of objects) and consumerism.

## 2 Stochastics and human biology

Stochastic reasoning/planning is a process used to account for risk and uncertainty when outcomes are unpredictable. One can map risks according to qualitative and quantitative stochastic modeling. Simulation generators (like Monte Carlo generators) are used to model many alternative sample paths/histories. These are modeled paths and not just outcome predictions (Taleb, 2004). The concept of “paths” embodies a wider range of contingencies, in comparison to a direct outcome analysis. Path analyses involve the *qualitative* study of sequential information, within any scenario, for all possible paths, over a certain period. One can uncover what outcomes are (im)possible, what event sequence leads to which outcomes, and possible stopping points upon path progression, including information as to which stops affect an outcome (and in what way). Although humans are poor at learning from history, alternative histories through stochastic modeling improve risk management strategies by rendering them antifragile (Taleb, 2004). These models highlight possibilities and omissions. Risk strategies can be built by comparing ratios and other *qualitative inferences* (Taleb, 2004).

Stochastic modeling occurs naturally in human biology. The prefrontal cortex (PFC) is part of the cortical system and the “emotional” limbic system (Sapolsky, 2017). The PFC is divided into (1) the dorsolateral PFC (PFCDL) and (2) the ventromedial PFC (PFCVP). The PFCDL is the rational, cognitive, utilitarian, and unsentimental decision-maker. It is also the last region of the brain to fully mature. The PFCVP is concerned with the emotional aspects of decision-making. Decisions and thoughts are thus intermingled with the “emotional” limbic system (Sapolsky, 2017). Both regions run real-time simulations of alternative histories. The PFCDL is concerned with utilitarian outcomes, while the PFCVP is concerned with the subjective “how would I feel.” This is responsible for that intuition one has about a course of action. The “correct” course of action resolves around the negation (or repression) of “failed” or unsuitable *non-existing*, but possible alternative histories, which are produced through the simulations. These simulations are a mechanism for determining the most *efficient* routes of risk management. This is thus a *repression of nothing,* as Freud noted about the unconscious (it is the repression of the fact that there is nothing to repress, which constitutes the unconscious). Details on “Freud” are given in Supplementary Material. It is a difficult concept to understand and accept, but it is trite that history runs forward and not backward (Taleb, 2007), which is the movement of regression functions, as mentioned above. The author has provided a navigational schema for the reader to follow demonstrating the various levels of interconnectivity and supplemental explanations in Supplementary Material.

## 3 Frame axioms: epistemology and computation

The frame axiom problem in programming involves how to frame or reason about problems—and also describes the problems in determining what changes, and does not change, as a consequence of certain events or actions. Frame axioms determine methods of making assumptions about the world, thus enabling agents to make predictions regarding actions and possible consequences (Allen and Ferguson, 1994). There are two issues to consider when deconstructing problems and predictions: (1) the epistemological problem and (2) the computational problem.

The epistemological problem involves the kinds of assumptions made about the world. The computational problem concerns the issues involved in determining how to compute and use those epistemological assumptions in a formulism (Allen and Ferguson, 1994). In terms of the epistemological problem, for example, intelligent design programmers must decide whether to make assumptions about any changes in properties or event occurrences. Computational problems, on the other hand, involve the issues in determining which kinds of techniques should be used in a model to implement those epistemological assumptions. Computational problems typically involve the use of explicit axioms, such as the situation calculus approach or the explanation closure technique.

## 4 Planning, prediction, and explanation

Sense (coherence) making and reasoning require three concepts: (1) explanations; (2) planning; and (3) predictions. Planning means having an initial description of a world or context and a desired goal. Actual planning describes finding a course of action that will be conducive to a goal. Explanations involve finding the best-fit system or model, which best fits the sets of observational data (Allen and Ferguson, 1994).

Plan recognition involves the prediction of an agent’s top-level plans based on observing its actions. It is an *abductive reasoning task*, where plans are inferred that best explain observed actions. First-order logic is often used for plan recognition, but these methods cannot handle uncertainty in data. The other option is probabilistic graphic models, but these cannot handle representations (ScienceDirect, n.d.). It is a problem when one observes other agents’ actions (data points) and wishes to obtain the agents’ plan in order to construct an explanation. This is known as the *plan recognition problem*.

As described in “The brain” in Supplementary Material, prediction is the process modality for world-building and correction (Hawkins and Dawkins, 2021). Prediction involves “foreseeing” or predicting the effects of new actions and events that will occur and then updating the model as required (Allen and Ferguson, 1994). If temporal logic is used, the world model will likely contain some information relating to the past or future actions and events. Prediction requires the creation of sequences of events or experiences based on the world models created and the information contained therein. Planning requires prediction to determine what actions will accomplish the set goals. Planning can be decomposed into (1) generating a set of candidate actions and (2) evaluating whether these actions will be successful (Allen and Ferguson, 1994). Explanations also require prediction. Explanations can be decomposed into the generation of a possible set of events that might explain observations, followed by a verification of whether the said events would cause the observed effects (Allen and Ferguson, 1994). Both thus would work on a predictive model to generate these steps.

Prediction is a probabilistic graphic model (Allen and Ferguson, 1994). Predictive information describes information within channel input about the channel output. Hence, this is a temporal movement “forward” in time. This information then relates to objects or events that do not exist at the time when the information is processed. Restorative information, on the other hand, is information contained in the channel output about the input (hence a “backward” temporal movement). This information relates to something that, at the time of processing, does not exist (in a specific form) as it did prior to the processing. A non-symmetric channel can have different effects on different symbols (relative), and the outputs can change. In these kinds of systems, there can be large distribution changes (because the physical processes that induce transmission are different for different symbols), including average abstract information quantity gains being made if the channel is used frequently.

Simple models, using a standard backward chaining planning algorithm, function by chaining backwards from the goal state (regression). The starting point is the goal state, which is compared to the initial state. This is followed by using a set of propositions, which differ in truth value between the states. Then, an action is enacted, which results in the obtaining of one of those propositions. The state of the world prior to the enacted action is computed using regression (which inverts the add and delete lists in an action definition). The new state now becomes the goal state, and the process continues until the initial state is derived. After this process, the algorithm now has a sequence of actions that lead from the initial state to the desired goal state. The predictive model here functions according to two broad assumptions: (1) no other events or changes occur in the world except for planned actions and (2) the action definitions completely describe all changes that occur as the result of the action (Allen and Ferguson, 1994). Prediction can be accomplished using these two steps. Regression techniques were designed specifically to exclude an explicit prediction step because of the assumptions (related to the incomplete absolute discussed below). This technique allows for the regression of an operator from state B to state A (as the preceding state) and the guarantee that predicting from state A with the same actions would yield state B. Using this, a plan can be constructed *in a backwards fashion*. Once a plan is found, it will achieve the said goals.

## 5 Explanation closure

Explanations are deductions, based on axioms, which assist with “sense-making” or reducing uncertainty. There are many difficulties in designing artificial intelligence systems and explanation axioms. When dealing with predictive models (the human brain too operates via prediction [Hawkins and Dawkins, 2021]), designers are faced with the problem of assumptions (epistemology, which is necessary in any design) that do not work. Reasoning processes are far more complex, which simply follow designed-in assumptions.

Situation calculus and temporal logic approaches, on the other hand, do not require those operational assumptions. These theories of logic do not commit to how states resulting from actions relate to states before actions. Instead, the properties of the resulting state must be specified through axioms; the framing problem revolves around how best to specify these properties (Allen and Ferguson, 1994). The first approach is to use explicit frame axioms, where each axiom is stipulated, each of which describes which properties are not to be changed by actions. However, this is an impossible task because there are too many axioms one would need to create (Allen and Ferguson, 1994). To overcome this, the frame axiom approach was relegated in intelligent designs.

The solution to the epistemological problem was to build models that utilize persistence or inertia assumptions. Here, the assumption is that all changes caused by an action are specified, and all properties not asserted to change do not change (Allen and Ferguson, 1994). This is an undesirable approach because if there is uncertainty about whether a property might change, the approach will erroneously assume that the property does not change. Other approaches focus on minimizing property changes or have constraints imposed on temporal ordering or properties or causal relationships. However, these approaches are problematic because they cannot handle simultaneous actions or external events well. Assumptions that are rather *based on events* lead to a more intuitive characterization of problems, wherein the logic would be related to an intuitive fact about the world (Allen and Ferguson, 1994). This approach handles a wider range and more complex problems.

The better approach is to *specify for each property what events can change it*, instead of trying to specify a host of different actions that could change a property (Allen and Ferguson, 1994). This reduces the problem of reasoning about changes to instead *reasoning about what events may or may not occur*. This includes both “external” events and actions of the agent itself. This is called *explanation closure*. Assumptions are still present in this technique but enable a large reduction in the number of frame axioms required to produce workable sets of axioms for a problem (Allen and Ferguson, 1994). Event-centric approaches to physics have gained ground recently, showing great potential to solve issues within physics. To observe a physical state associated with the probabilities generated from quantum mechanics is impossible because one needs an uncountable set of identically prepared quantum states and measurement apparatus to perform continuous measurements. Every bit of information collected from the environment is a consequence of discrete interactions between material objects—these interactions are called events. Instead of the notion of “particles” or “fields,” events offer different axioms for physical systems. Within an event-centric paradigm, the continuous evolution of particles/fields is replaced with the discrete evolution of causal networks of events (Powers and Stojkovic, 2023).

Explanation closure axioms are a method of treating events on a case-by-case basis. This allows for idiosyncratic events and properties to be represented, even though they do not fit the norm. These approaches code solutions into the axioms themselves. Some have dubbed this “cheating” since it provides the agent with some explicitly encoded assumptions that would make the representation work (Allen and Ferguson, 1994). However, this is part of a common-sense logic of the world that agents would require. They are also problem-independent. Furthermore, it is not accurate to suggest that all these axioms need to be “programmed in” by coders—they can in fact be generated automatically during action or “on the fly” (Green, 1969), which means that they are *separable from the formulism*, unlike other approaches (Allen and Ferguson, 1994). This is known as contextual import. Mechanisms of backward propagation loops or regressive functions include the processes of *iteration* or *recursion.* Iteration is a context-independent constraint that *feeds back information from the output* of one trial run *into the initial conditions of the next run*. Hence, iteration acts as a temporal constraint, *which alters the probability of the next output*. The iteration process is repeated. *Recursive iteration* is a process wherein full sequences are fed back on themselves. This form of looping results in processes and sequences becoming *self-referential*. When the last step of a sequence feeds back into itself *to become the first step of the next iteration*, *a self-referential configuration* with *non-linearities* is created. The latter introduces multiscale and multidimensional interdependencies (Juarrero, 2023). Thus, iteration and recursion are processes that import/incorporate meaningful information from contexts/the world back into the system through the alteration of the weights of the middle-layer connections in the algorithm. The system can thus become more suitable and calibrated to its context. This also allows for *qualitatively novel* results/features to emerge. Contextual import *enables representation to be possible*. Properties here only change upon the occurrence of certain events. Furthermore, importantly, these assumptions do not need to be correct; where wrong or false, they also need to be made explicit in the representations (Allen and Ferguson, 1994).

The solution to the computation problem (or what mechanism can be used to make assumptions) is to either (1) explicitly add axioms that encode all assumptions or (2) use a nonmonotonic model theory that defines a new notion of entailment, including the assumptions (Allen and Ferguson, 1994). There are many ways to do this, with each having positives and negatives (which are mostly reduced to the ease at which a formulism can be achieved). Explanation closure axioms, on the other hand, allow for a flexible system that can handle complex issues in representing actions. The representation that results from this will operate in standard first-order logic, thus making it relatively easy to determine if consequences follow from axioms. Furthermore, the handling of exceptional cases does not require extending language syntax to include special predicates, which complicates the reasoning process and can lead to unintuitive formalisms (Allen and Ferguson, 1994).

## 6 Hegel’s theology: events and virtuality

Hegel’s radical re-interpretation of Christian theology involved the birth of Christ as God only being able to recognize his own existence through the Othering of himself (as the form of Christ) (Žižek et al., 2011). The death of Christ on the cross symbolized the death of God (the transcendental God, or the Platonic God) or his belief in himself. Hegel, in forming his account of embodiment, merged the transcendental and materialist positions—the Absolute is to be understood as both substance and subject. Žižek (2003) elucidates this position and calls for the abandonment of the traditional view of Hegelian Spirit:

“The point this reading misses is the ultimate lesson to be learned from the divine Incarnation: the finite existence of mortal humans is the only site of the Spirit, the site where Spirit achieves its actuality … Spirit is a virtual entity in the sense that its status is that of a subjective presupposition: it exists only insofar as subjects act as if it exists. Its status is similar to that of an ideological cause like Communism or Nation: it is the substance of the individuals who recognize themselves in it, the ground of their entire existence, the point of reference which provides the ultimate horizon of meaning to their lives, something for which these individuals are ready to give their lives, yet the only thing that really exists are these individuals and their activity, so this substance is actual only insofar as individuals believe in it and act accordingly. The crucial mistake to be avoided is therefore to grasp the Hegelian Spirit as a kind of meta-Subject, a Mind, much larger than an individual human mind, aware of itself: once we do this, Hegel has to appear as a ridiculous spiritualist obscurantist, claiming that there is a kind of mega-Spirit controlling our history … This holds especially for the Holy Spirit: our awareness, the (self )consciousness of finite humans, is its only actual site … although God is the substance of our (human) entire being, he is impotent without us, he acts only in and through us, he is posited through our activity as its presupposition.”

Hegelian Spirit is thus a *virtual entity*, whose existence actualizes (or becomes) upon the recognition within the registers of the subjects—via belief, for example. In this way, the *virtual becomes actual* within subjective beliefs; however, that actualized virtual is not reducible back to the said subjective beliefs. The virtual event is constituted by the subject as such—because *beliefs are a response* to events (they occur after events). *Subjects interpret events as (to be) events.* The critical question here is whether events are only events upon recognition and registration by subjects.

Some have argued that the ability of subjects to register events as such is not a requisite for events to be events in themselves (Žižek et al., 2011). The counter argument is that events themselves are instantiated into things as part of the virtuality of the substance itself, which, when expressed, are then registered by subjects and their beliefs. Considering this, the Spirit would be that which arises in response to events and performs an interpretation after the occurrence of the event. Žižek’s position is that of a “subjective presupposition”; however, the counter argument advocates that events can be constituted without a subject. The virtuality of events may be registered by subjects, but it can also be registered in the energies of “things” and, hence, may be “felt” in things themselves. The virtual, in this way, is also substance and subject, which is *ex post* realized as such by subjects, who then name it as such. Thus, in the Hegelian ontology, God takes the *form of events*.

## 7 Dialectical discrete event-centric physics: events and observers

Events can be construed to be the building blocks of spacetime. In the current relativity theory, events are understood to be discrete units of volume in spacetime. Importantly, an event-centric approach allows for the mathematical structures in general relativity to be constructed using *discrete elements*. This offers solutions to the two most important issues in modern physics and, hence, can be used to construct a new interpretation of physics (Powers and Stojkovic, 2023).

Primitive network elements can form part of a bigger causal network of events. These primitive network elements consist of two events, which can share direct or indirect causal connections. A direct causal connection implies that they are related through a third event. Indirect causal connections, on the other hand, correspond to experiments involving entangled particles, such as the Bell test (Powers and Stojkovic, 2023).

*Observers* are to be construed as entities capable of assembling information about events (Naidoo, 2023a; see “Topology and spacetime” of Supplementary Material). Observers can use this information to construct physical models, which can be used to infer properties of future events. To obtain information about events, an observer must participate in the said event, which means that events must have a structure. This structure is a one-part system and one-part observer (Powers and Stojkovic, 2023). This means that an observer and an event are *reciprocally constitutive* (Naidoo, 2023f). Thus, as mentioned above, events are only events as such upon registration in the registers of subjects. This accords with the QBism (Naidoo, 2023e) interpretation of quantum mechanics, whereby quantum states (as a navigational tool) are interlinked with subjective beliefs regarding the outcomes of experiments (Caves et al., 2002). This thus involves an inherent interlinking with a (subjective) Bayesian (and surprise-oriented) approach to probability (Naidoo, 2023a; Naidoo, 2023f). Bayesian probabilistic inferential reasoning is a highly accredited theory regarding human reasoning in the cognitive and sensory domains (Pouget et al., 2013).

The notion of observers is thus important and should be part of any model of physics being proposed (Powers and Stojkovic, 2023). The act of measurement ought to be understood as the performance of an experiment and can be described as the revelation of the pre-existing state of the system under the study. “System” is to be understood as two causally connected events (below), which has a wide range of implications. The kind of event-centric view used in the study (Powers and Stojkovic, 2023) is naturally implied by quantum contextuality and is also related to Bell’s inequality.

## 8 Maps and events

Maps are to be used as the basis for physically observable variables (see “The brain” and “Topology and spacetime” in Supplementary Material). For a single event, a map can be used to generate a second event in a way that the important aspects of the causal relationship between those events are encoded within the map (Powers and Stojkovic, 2023). If taken independently, however, neither the initial event nor the map will contain enough information to completely determine the second event. The information is stored in the causal relationship between two events, which are related through the map. To summarize, a map thus connects two events.

Novel properties can emerge from this causal relationship, and these novel properties can be distinguished from those associated with events and maps using the notion of *locality* (Powers and Stojkovic, 2023). Locality describes properties that are associated with either event 1 or event 2 or of the map itself (see “Quantum theory” in Supplementary Material). Locality thus describes *non-emergent properties*. *Non-locality*, on the other hand, describes properties which are emergent from the causal relationship itself. These are *non-local degrees of freedom*. Events can be described as measurements that are performed by observers. In other words, events are only as such, when they are inscribed into the registers of subjects, as observers (Naidoo, 2023a). The nature of this registration thus depends on how, or the type of rationalization/explanatory theory/interpretation, the subject affords to the event.

## 9 The Absolute

The “Absolute” is a concept originating from German philosophy and is a general term used to describe the metaphysical conception of a fundamental “totality.” The Absolute is thus that which is self-sufficient, meaning the “thing” upon which all other things depend, but which itself supposedly depends on nothing outside of itself (Žižek et al., 2011). The Hegelian conception of the Absolute aimed to overcome dualisms, especially those in Kantian philosophy (such as phenomenal versus noumenal worlds), by providing a metaphysical grounding for these dualisms. The Hegelian conception of the Absolute thus was a unifying metaphysical ground for all knowledge (Moyar, 2017). In legal–political terms, the Absolute corresponds with the governing state, and its corresponding laws, which delineates the bounds of freedom for persons.

The concept of the Absolute as such was ambiguous, in that it undermined nihilism, while also threatening to be nihilistic. In the first sense, it undermined nihilism as non-linearity was implied, thus simultaneously undermining the concepts of “finite” and “finitude.” In the second sense, it is nihilistic in itself as it seemingly eliminates “free subjectivity” because it implies absolute predetermination (and not independence) by an Absolute totality (Moyar, 2017). This means that there would be no individuality, free choice, or free will, and the finite would be illusory and be akin to the Kantian “puppet-on-a-string” (Žižek, 2003). Hegel’s solution was thus to construct a theory wherein finite subjects, and finite objects, are positive “moments” in the Absolute. Thus, Hegel posits the Absolute as being an *internally differentiated whole* (Moyar, 2017).

## 10 The incomplete Absolute

During its unfolding, the Hegelian Absolute passes through a process of its own becoming, which involves the Absolute realizing that a part of itself must always remain beyond itself. The Absolute thus sublates its otherness, in its identity. Gabriel (2011) noted how this also means that the Absolute is simultaneously finite and infinite:

“The absolute idea is only grasped in the context of a theory of self-constitution of logical space, i.e. of the concept in an eminent singular … The answer, therefore, to the question: how does the infinite become finite? is this: that there is not an infinite which is first of all infinite and only subsequently has need to become finite, to go forth into [herausgehen] finitude; on the contrary, it is on its own account just as much finite as infinite.”

Gabriel (2011) noted that

“This movement of the negation of negation is precisely what takes place in the chapter on ‘the Absolute’ in the Logic, the introduction and first subchapter (A) of which proceed in three steps. First the absolute is determined as absolute transcendence, or as absolute identity which outstrips our conceptual capacities. It can only be paradoxically determined by the negation of all predicates. Second this movement, which is a movement of reflection, is made transparent as reflection. In order to steer clear of the problem of absolute transcendence, the finite is determined as an image of the absolute, which has being far more than any finite being due to its pure positivity, a position Hegel ascribes to Spinoza … Third this whole movement is presented as a process by which we eventually arrive at the form determination of the absolute form, where form and content of reflection coincide in the ‘self-exposition’ of the absolute, i.e. in the reflection of reflection.”

This means that the Absolute cannot exist as a preconceived/presupposed substance prior to the process of its own becoming; hence, the Absolute retroactively posits/manifests itself through the logical process of self-negation and the negation of determinate concepts. Hence, it is a retroactive position of the Absolute from a logical space (Gabriel, 2011). This Absolute is both the form and the content of logic, and it is not something prior to the manifestation of itself in logical thought. This is the logic of the “return of the repressed,” which Freud later expressed, that constituted the unconscious as discussed.

This kind of unfolding is thus a movement of *pure thought*, which Hegel (2010) described in the *Science of Logic*. This movement of pure thought means that the Absolute is just a grounding concept that makes the finite intelligible, but the Absolute itself does not have any content other than being the grounding that separates two relata (and, thus, two entities). Why is this so? Because of reflection. By reflecting on a transcendental Absolute, reflection must think “beyond itself” (Gleason, 2021). Hence, reflection must then negate all predicates (of existence). However, by doing this, reflection just creates the Absolute as a transcendent only insofar as the Absolute cannot be determined by predicates. The Absolute in this way is undermined because it would then be determined as that which cannot determine it. Hence, it would not be a true Absolute since it is dependent in this way. Hegel then moves from external reflection to Absolute reflection, the result of which posits the difference as being internal to the Absolute, and not external to it. It is this very movement that constitutes the Absolute. Žižek (2014) explains this as follows:

“This, then, is the dialectical process: an inconsistent mess (first phase, the starting point) which is negated, and through negation, the Origin is projected or posited backwards, so that a tension is created between the present and the lost Origin (second phase). In the third phase, the Origin is perceived as inaccessible, relativized – we are in external reflection, that is, our reflection is external to the posited Origin which is experienced as a transcendent presupposition. In the fourth phase of absolute reflection, our external reflexive movement is transposed back into the Origin itself, as its own self-withdrawal or decentring. We thus reach the triad of positing, external reflection, and absolute reflection.”

This means that the Absolute is internally incomplete. The transcendent is internal to the immanent as the Absolute is always already beyond itself. In fact, the Absolute is constituted precisely by its own failure to fully grasp itself. The expanse between the transcendent and the immanent is internal to the constitution of the immanent–transcendent, as noted by Gabriel (2009) on this–“The crucial point of Hegel’s dialectic of the absolute is that metaphysical reflection must not be external reflection. We cannot determine the absolute as absolute substance ontologically anteceding our conceptualization of it. Therefore, reflection has to become absolute, i.e. self-referential.”

The Absolute self-discovers itself as infinite, and in doing so, this is simultaneously how it limits itself and surpasses its own self-imposed limits. Hence, there are parts of the Absolute that are unknown to the Absolute itself. The infinite is then the Absolute’s ongoing process of self-constitution, which is not determined by anything external to the process itself. This means that there is a realization of the overall coherence of the overall movement. Simply put, consciousness discovers that it is the Absolute itself that moves between self-transcendence and identity (Gleason, 2021). Absolute reflection then is the difference between the immanent and transcendent as being internal to the Absolute itself. It operates in such a way that it was the Absolute itself that distanced itself from itself. The Absolute is thus the oscillation of thought between indeterminacy and determinacy (and *vice versa*) in time (Gleason, 2021). The immanence of the Absolute is, thus, not exclusionary to the world of experience (see “Retroactivity and logic” in Supplementary Material for a noteworthy summative quote).

## 11 Epistemic constraints and possibility

The study accounts for the seemingly non-deterministic property of nature through enforcing an *epistemic constraint on observers*. The information, which observers are permitted to have about the causal relationship between two events, is limited to *local counts* (for information on locality, see Naidoo, 2023c). This implies that the information stored in the ordering of symbols (see “Symbols” in Supplementary Material), within events and maps, is *hidden* from observers. This accords with Hegel’s assertion about the *content hidden in the form* (Naidoo, 2023b) (see “Content hidden in form” in Supplementary Material). This hidden information is that of qualitative meta-degrees of meta-freedom, which amounts to the freedom to interpret and re-interpret (Naidoo, 2023a; Naidoo, 2023b).

Under this constraint, *one can interpret a particular choice of local counts* as specifying the macroscopic state of the causal network, which is analogous to a quantum state (Powers and Stojkovic, 2023). For any macroscopic state, there will typically be many associated microscopic states, each of which is modeled by a unique sequence. These microstates represent possible ontic states (or real physical states) of the underlying causal network. This epistemic constraint secures an *irreducible* ambiguity, which, in turn, ensures that inferential and interpretative capacity is non-eliminable (Naidoo, 2023a). In other words, shades of freedom lie within *qualitative theorization,* as opposed to quantitative application (Naidoo, 2023a). How is an irreducible ambiguity constructed? The solution that Naidoo (2023a) presented is that of *meta-difference*. This will be explored in detail in further work; however, ambiguity does express itself in the following ways: (1) the Kantian transcendental apperception/imagination (Naidoo, 2023d); (2) the incomplete Absolute as explained above; (3) Darwin’s utter extinction (see “Utter extinction” in Supplementary Material); and (4) the Kantian infinite/sublime judgement (below).

## 12 Ambiguity, evolution, and novelty

Context-independent constraints are preset configurations concerning dimensions and possibilities. They (context-independent constraints) are limits to multivariate/multidimensional landscapes. The boundaries and contours form the initial conditions that represent the context-independent constraints. These context-independent constraints bias the direction of energy flow, but factually, they do not *strictly determine from the outset which, of the multiply realizable alternatives within that possibility space, will be realized*. It is thus *not true* that context-independent constraints *are determinative of identities or outcomes*. For example, context-independent constraints mapped one-to-one with a given phenotype would render it impossible for any variation, specification, or individualization to occur other than “by-chance” mutations. Correlatively, the cosmos would have never been able to evolve in the past or at present. The cosmos would never reach the complexity we know it to reflect today (Juarrero, 2023).

For the kind of complexity we observe today, the initial constraints must be vague/ambiguous in terms of their scope/reach. In other words, the initial constraints must not be fixed and must rather be flexible or in flux. Context-independent constraints are thus flexible and contain “feasibility regions” wherein subsequent constraints can interact—similar to a stage whereby a variety of different narratives or plotlines play out/simulate (Juarrero, 2023).

Furthermore, maintenance of any kind of dynamic equilibriums or homeostasis (in terms of biological bodies) requires a continuous balancing and re-adjustment of bodily properties *in lieu* of the context (Juarrero, 2023). A balancing of this sort requires flexibility. Lastly, even though context-independent constraints bring systems into non-equilibrium, they do not produce complexity or persistent structures or dynamics. They also cannot transmit complex messages (although they can aid fidelity of the transmission of communication systems). Hence, they cannot be foundational for complexity formation. How then does complexification arise, and what does flexibility even mean?

First, flexibility simply means that context-independent constraints must be *ambiguous, vague*, and *allow for multiple realizability* (of the various hills and ridges of the epigenetic landscape). What is necessary then to achieve flexibility are *context-dependent constraints* that enable context adaption, maintenance of dynamic equilibrium, and specific realization (Juarrero, 2023). In terms of complexity, the same reasoning applies. Complexity in biology, cosmology, or social systems requires two kinds of constraints, i.e., context-independent and context-dependent ones. Both operate concurrently and do interact with one another and as a unit.

Within the evolutionary discourse, this is represented by Waddington’s landscape with hallows and hills, which arise from different, multiply realizable constraints.

According to Waddington, who based his epigenetic topographic-landscape theory on dynamical system theory, different dimensions of the landscape correspond to different physical quantities and qualities. Ontogenetic developments and differentiations are like a ball rolling down from a ridge into a valley, which are representative of context-independent constraints (Walsh, 2015). The trajectory of the ball is directed by the contours of the walls of the valley. Any perturbations to the system would result in the ball moving up on the side of the walls and then back down toward its regular path—in this way, the topography of the epigenetic landscape secures a robust development of forms (Walsh, 2015). In the presence of significant perturbations, the ball can scale and overcome the walls, which form the epigenetic buffer development against systemic shock. The results produced by the ball overcoming these buffering valleys are strange phenotypes, known as “phenocopies.” The production of phenocopies, according to Waddington, can alter epigenetic landscapes and reshape them in such ways that novel phenotypic traits become increasingly canalized (Walsh, 2015). These are exceptional phenotypes/traits, which are unstable but can become stable through processes of genetic assimilation.

In short, the motion of the ball thus influences and changes the landscape; they are mutually constitutive. In the words of John Wheeler—*space tells matter how to move, and matter tells space how to curve*. Novelty too can be introduced by environmental perturbations as well, thus resulting in the obtaining of a novel, exceptional phenotype/trait (in the form of a novel path/valley created), which can be stabilized by a genetic system. In this way, the previous trajectory/path, including the determined outcome, is negated, with a new outcome *in the process of being obtained*.

This system proposed by Waddington demonstrates that even initial fundamental constraints are not deterministic and are in fact *vague* (Juarrero, 2023). Hence, even initial constraints allow for the enactment of later local time-dependent contextual constraints, which can add complexity to systems while delaying heat death in the form of the second law of thermodynamics. As mentioned earlier, events, as perturbations, require co-constitution via subjective interpretation/registration. In this way, there is an agency afforded to any system, relative to the system’s ability to respond to and interpret perturbations, thus facilitating their own evolution, as proposed by Naidoo (2023f). In cryptography, ambiguity is secured through by using a cryptographic tool known as indistinguishability obfuscation, which renders programs unintelligible, while still preserving functionality (Jain et al., 2020).

## 13 Ontic-state spaces and encoding

If the information observers have about causal networks is limited to macroscopic data, this information must be treated statistically. This means that the information an observer has about a given causal network *will always* take the form of an ensemble of microstates called the ontic (like ontology)-state space (Powers and Stojkovic, 2023). If events are separated in spacetime, then each event must have a separate ontic-state space. The ontic-state spaces are associated with two different observers or with the same observer who observes at two different times. Each different kind of observer will encode observed macroscopic information about an event available to them, at the site of the event. Quantum theory here *describes the discrete evolution of statistical ensembles of causal networks* (by counting the path between each state space) (Powers and Stojkovic, 2023). It has already been demonstrated that discrete formulisms can support physics models (Powers and Stojkovic, 2023). Hence, this is a model for a *non-deterministic system/nature*. To be more precise than the study, this is an *asynchronous* system, i.e., a system that is non-linear or ordered by causality, temporality, or physicality. The organization of the system is that of selective synchronization, with parts working independently and coordinating through non-linear and non-physical/logical means, such as information transmission/propagation, beliefs (Naidoo, 2023g), or other forms of subjective registration/recognition. Thus, the full extent of the synchronicity of the system is contingent on subjective recognition. In the presence of a *lack of recognition* (ignorance) or an *intentional misrecognition* (an intentional negligence—also known as the Kantian infinite judgement, as explained below), there is no mutual or dialectic recognition and, thus, constitution, thus leading to the event not being constituted. This is also akin to the plan recognition problem as mentioned above. For example, in South Africa, the “open society” is an underlying constitutional principle, tethered to other constitutional values such as freedom and dignity, which are implicit. These values require subjective registration to be actualized (see “Sedimentation and memory” in Supplementary Material). For example, a provision is not unconstitutional, or not constitutionally aligned with the values and ethos of a constitution, until it is demonstrated to be—through subjective demonstration/interpretation or construction. A state constitution represents a 1:1 mapping with any system constitution (or mind).

## 14 Inferences and variables

The non-local emergent properties mentioned are those that drive inference. Events, as measurements, can take the form of two separate observers or the same observer at different times (known as the split-brain [Naidoo, 2023a]). These are two sources of information that would *fully characterize an outcome* (Powers and Stojkovic, 2023). Each ontic space contains the encoded information of each observer, measured in their respective physical contexts.

The physical context can be identified using quantum numbers and divided into four categories: (1) random variable; (2) conditioning variable; (3) local nuisance variable; and (4) non-local nuisance variable. The conditioning variable can be described as a continuity parameter in models that enables probabilities to become arbitrarily smooth (see “(Self)entrenchment and flexibility” in Supplementary Material). These variables are selected or controlled in model experiments. Random variables, on the other hand, are physically observable variables that are neither selected nor controlled (explanation closure axioms). They can take on any value permitted by the conditioning variables. Nuisance variables are physical quantitates, which are not observed in the experiment being performed.

This can be translated into the legal lexicon, being that of the four statutory interpretative methods used. The conditioning variable is the literal interpretative method, given that the immediate language of a statute typically implies a bounded rationality (thus smoothing the probabilities into a set of possibilities). Random variables are akin to the contextual interpretative method, given that contextual information or knowledge oscillates rapidly, which is not controllable, but rather subject to any particular time, place, and epoch. Random variables are akin to *subjective* purposive interpretation (instances of agency). Local nuisance variables are akin to the constitutional interpretative method, speaking to the immediacy aspect of constitutionalism, being the wording of the constitutional documents themselves. *Non-local* nuisance variables are the histories, values, and philosophies (like the open society) underlying specific constitutions, which are merely implicit and not explicitly stated. These non-local instances require subjective actualizations to come into being, given that they are merely implicit. This renders non-local (and local) nuisance variables, largely contingent on subjective construction.

## 15 Probability

Within this formulism, probabilities arise because of *hidden information*, which implies that *probability is epistemic in origin* (Naidoo, 2023a; Powers and Stojkovic, 2023). *Ambiguity* is thus epistemic in origin. State spaces that are associated with distinct choices of observables *are always disjoint* (a Hegelian and Freudian concept), which means that no single-base 16 sequence will ever appear in state spaces associated with two different sets of observables (see “Freud” in Supplementary Material). The frequentist interpretation of probabilities states that the size of the conditioning variable (n) will have a significant impact on the size of the state spaces and, hence, on the continuity of probabilities.

If (n) is small, Alice and Bob (as two different observers) will be able to perform enough experiments to have observed all possible ontic states (if we assume that no ontic state will ever occur more than once). Upon doing so, the predicted probability and their measurement results will match exactly (which is in line with the frequentist approach to probability). Another consequence of a small (n) is the loss of statistical independence (assuming once more that no state occurs more than once). Hence, Alice and Bob’s past measurement outcomes would influence what they will know about future experiments or measurements (*memory*). This is a reciprocal/dualistic approach to self-constitution, which leads to each observer to secure (or “predict”) the results of their future observations/experiments. This leads to a possibility for completely deterministic experiments (see “Negation and prediction” in Supplementary Material). In computer science, “fuzzy logic” is used to model human decision under vague information (Zadeh, 1999). Recently, important links have been established between fuzzy logic and Bayesian inference (Gentili, 2021).

Relating back to contextual import, both iteration and recursion interweave the subject’s/system’s own history, paths, choices, and outcomes back into the subject/system. *Feedforward processes* can *modify themselves by using contextual information* as described to anticipate expected conditions and events. Feedforward loops are those in which the *attractors are anticipatory (predictive).* It is important to understand that self-modification or anticipation describes *probability distribution changes relating to events within a possibility space* (Naidoo, 2023g; Juarrero, 2023). More information on securing a desired state, instead of trying to predict it, is given in the study by Naidoo (2023g). This also circumvents the is–ought false dichotomy.

## 16 An ontology of reality

Events that are “fixed” are directly related to their ontological status. All observed properties of these fixed events are taken to be definite states of reality. Any base symbols within an event, whose counts are observed during an experiment, *cannot* vary in configuration within the associated observer state space. When the four base-4 symbols are held fixed in either observer state space, the assumption is that all four quantum numbers associated with each of their events are either random or conditioning variables. This experimental design requires *two sources* (duality) of information to fully characterize an outcome; hence, the *product of both observer state spaces* is fundamental to calculate probabilities. The *joint state space* (a *dialectic*) represents that both observers have reached a consensus (Naidoo, 2023a) on all local quantum numbers for each experimental outcome (Powers and Stojkovic, 2023) (see “Reciprocal self-constitution” in Supplementary Material).

In this formulism, the information that observers are permitted to have regarding a causal network is *limited* to local quantum numbers, which results in indistinguishable ontic states. Indistinguishable ontic states are linked to permutations and variance. The first type of the indistinguishable ontic state arises from permutations in local quantum numbers; *the invariance* of both observers’ base-4 sequence is maintained. This symmetry arises because the information hidden in the ordering of the symbols constituting a sequence is hidden from observers.

The second type arises from permutations that lead to *variations in the non-local quantum numbers*, while the local quantum numbers remain unchanged. Here, the symmetries of local quantum numbers are maintained, but not that of the non-local quantum numbers (Powers and Stojkovic, 2023). These symmetries arise because the numerical values of the non-local quantum numbers are hidden from observers. Both symmetries describe the nature of hidden information—if information was not hidden from observers, then no two ontic states would be indistinguishable. Hence, there would not be any meta-degrees of freedom, for inferences or interpretation. In other words, the system of reality is determined to be open (Naidoo, 2023b; Naidoo, 2023e; Naidoo, 2023g).

## 17 Nihilism: linearity and optimism

Linearity results in feelings of nihilism, which is a lack of intrinsic purposiveness or optimism, since life becomes nothing more than a transient stop from the abyss to the void of nothingness instead of being purposive. This inherent groundlessness can be liberating since the absence of an underlying or guaranteed truth/objective can result in the unrestricted maximization of freedom. Ultimately, everything can be upturned or changed since nothing is grounded (Naidoo, 2023e). However, the focus on the point of nothingness, as being the origin, and endpoint/destination reduces optimism in individuals and society. The typical attitude of “get to the point” in all social and professional spheres of life demonstrates an inherent linear nihilistic tendency as if the point (or nothingness) itself is the purposive of things. This kind of thinking is *prevalent in academic culture.* Society fails to realize that information only exists *if there is no point*—that is why it is called in-formation (or evolving). *It is that which is currently in formation and, thus, unfinished or incomplete.*

The dopaminergic system is a double-edged sword (Sapolsky, 2018). Dopamine is about reward. Dopamine is created in multiple regions such as the *ventral tegmental* (VT) area located near the old brain stem. Certain stimuli activate the VT area, which then activates the release of dopamine. Chronic stress or pain decreases dopamine and the sensitivity of the NA, leading to depression. The pleasure system is primarily probability-based; the higher the probability, the less dopamine released, the lower the probability, the more reward released. Thus, *the dopamine reward system is not absolute and is instead relative to the reward value of alternative outcomes* (Sapolsky, 2017). Habituation results in less dopamine release. It is the *surprise* factor that determines how much dopamine is released. Thus, dopamine’s motivational function (“feeling good feeling”) is not concerned with the reward or outcome, but rather, it serves to drive action to obtain an outcome based on the *anticipation of a reward and not the reward itself* (Sapolsky, 2017). This anticipation builds up and requires learning—which comes from contexts—and *is not inherent*. It is this modality of motivation that *builds optimism* and drives action (binding the reward to action) to pursue an *uncertain* or *unpredictable* eventuation of a reward. From this, we can surmise the following:

• Human reward systems function to reward failures to correctly predict (an unexpected surprise). Incorrect predictions thus lead to a reward—there is pleasure in pain!
• Rewards and pleasure are linked to fundamental biological drives, including reproduction, social status, and organization, but they can also be ontogenetically programmed into the brain. They can be developmentally/contextually based.
• Rare states, things, or achievements require more reward for anticipatory motivation because they require more energy expenditure; less rare states release less reward because of the converse.
• Human reward systems motivate the pursuance of rarer states, things, and experiences. Humans thus want what they cannot have or what is difficult to have.
• Thus, the human reward system sets us on a path that has a great risk of failure because rarity is a risky pursuit.

Paradoxically then, *the linear movement of efficiency, also known as optimization, functions inversely to dopamine release and, thus, enjoyment*. The reward system seeks out and functions on non-linearity or *uncertainty*. The certainty inherent to linearity is harmful to human optimism and enjoyment. This begs the question of whether there is any scope for freedom, autonomy, or enjoyment? Both Kant and Freud have answers for this, which, given the above, have now been validated.

## 18 Kantian aesthetics: the Sublime

In the *Critique of Power Judgement*, Kant begins by discussing the difference between the “beautiful” and the “Sublime.” For Kant, beauty is connected to the form of an object, which has boundaries or containment, whereas the sublime is formless; to be more precise, the Sublime is described as boundless or formless (simply formlessness is ugly and produces displeasure) (Doran, 2015). Both are concerned with *feeling*. In describing the sublime in *Observations on the Feeling of the Beautiful and Sublime*, Kant (1987) says

“For what is sublime, in the proper meaning of the term, cannot be contained in any sensible form but concerns only ideas of reason, which, though they cannot be exhibited adequately, are aroused and called to mind by this very inadequacy, which can be exhibited in sensibility. Thus the vast ocean heaved up by storms cannot be called sublime. The sight of it is horrible; and one must already have filled one's mind with all sorts of ideas if such an intuition is to attune it to a feeling that is itself sublime, inasmuch as the mind is induced to abandon sensibility and occupy itself with ideas containing a higher purposiveness.”

The Sublime presents the Kantian mind with a special kind of dissatisfaction. On the necessity of the Sublime, Kant (2015) says

“…that which excites in us, without any reasoning about it, but in the mere apprehension of it, the feeling of the sublime, may appear as regards its form to violate purpose in respect of the Judgement, to be unsuited to our presentative faculty, and, as it were, to do violence to the Imagination; and yet it is judged to be only the more sublime.”

Kant argues here that the rational faculty of the mind paradoxically requires the contra-purposiveness of the Sublime (hence making the Sublime a purposive–contra-purposive necessity for the whole mind itself). The Sublime is thus that without a purpose, which is a requisite for purposiveness as the Beautiful (of the Understanding) itself. For Kant, subjective purposiveness of the mind is produced through the unison of the Kantian Imagination and the Understanding (when judging the beautiful) (Doran, 2015). In that same light, the unison of the Imagination and Reason produces their own subjective purposiveness *through conflict*.

We experience the Sublime as a sort of transcendence as freedom from sensible constraints because of the asymmetrical conflict between Reason (as the higher faculty) and the Imagination as the lower faculty. Reason imposes its superiority over the Imagination (Doran, 2015). This is a transcendence not by harmony in unison, *but rather by agonism*. The Imagination is this overwhelming capacity of the mind; Reason is this rational capacity that steps in when the Understanding is overwhelmed by the magnitude or force of something (Kant, 2015; Žižek, 2020).

Orgasms release a huge amount of dopamine (as mentioned above). Importantly, as Sapolsky noted, during orgasm, *the amygdala deactivates* in both men and women. Both sex and aggression activate the sympathetic nervous system (SNS), and there is no distinction in the heart rate in states of orgasm and states of murderous rage (Sapolsky, 2017). In other words, as Kant noted, an experience of the Sublime involved the subjectification of the Understanding (experience) to the higher faculty of Reason.

Kant splits the Sublime into the Mathematical and the Dynamical Sublime. In brief, the Mathematical Sublime is that which is the judgment related to the esthetic estimations of magnitude (Doran, 2015). It concerns itself with ideas of boundlessness and formlessness, especially in reference to “totality.” The Dynamically Sublime is the esthetic judgement of nature as a power (which derives from the need for autonomy as opposed to totality) (Doran, 2015). The transcendence produced by agonism is a *felt pleasure in pain*. The pain is produced by the overbearing grandeur of the Mathematical Sublime or the dominating forces of the Dynamical Sublime of nature (Doran, 2015). The pleasure that follows this pain arises through Reason’s own transcendence into the supersensible (Doran, 2015). The Sublime is a thing of the mind itself, a mental elevation that comes from the transcendence of sensibility, i.e., the abandoning of sensibility in the pursuit of ideas that contain a higher purposiveness (Kant, 2015). *Reason introduces the idea of infinity, which only it contains* (Žižek, 2020). This Kantian idea is supported by renowned mathematician David Hilbert (1925) in his discussion on the mathematical concept of the infinite in his paper called *On the Infinite.* Hilbert argued that the infinite was fundamental to all thought and reason including mathematics itself. He even went insofar as expressing explicitly that Kant was right. The infinite thus was a constitutive necessity for all thinking and arose within thinking exclusively.

The idea of infinity is much larger than any magnitude an object can present to our understanding, hence diminishing the overwhelming sensations magnitude presents. In this way, we shift from our sensory experiences to a recognition of the higher transcendental powers of Reason that can ideate about infinity (Žižek, 2020). *This is a transcendence through a power of resistance*. It is a pleasure in overcoming. The faculty of Reason *transcends through failure* (by positing the infinite or the *an sich*—something that it can only circumscribe through failure). This is transcendence through de-sublimation.

There are two kinds of objects, namely, the beautiful and the ugly. Ugly objects are divided into contra-purposiveness and purposefully contra-purposive (Doran, 2015). The contra-purposive objects are those that are plainly ugly, while those that are purposefully contra-purposive (in the sense of being boundlessly formless) can be used to create the transcendence mentioned. This is a contingent judgment; in other words, one in which the mind itself reveals its own purposiveness by using “nature” as a means to an end (thus giving it its relative status) (Doran, 2015). This modality of judgement, of which the it purpose is to create a *transcendence or super-sensible feeling*, is not based on the concept of the object itself, *but rather a subjective purposiveness of the mind itself* (Doran, 2015).

In the *Critique of Pure Reason*, Kant (1890) presented the antinomies that result when pure reason tries to access the noumenal world. These antinomies are the (1) mathematical antinomies and (2) dynamical antinomies. The Kantian *ding an sich* could only be circumscribed through failures produced by the antinomies. These indeterminate concepts of Reason (Reason ideating) are the Sublime. They are *fruitful failures* because although one cannot have a positive knowledge of them, *one can know what it is not*. Hence, one can circumscribe the Sublime through failures (sort of like bumping into boundaries of a thing without knowing what the thing is itself). The Sublime is thus defined by its very *indefinability*. It is an aesthetic judgement, which refers not to objects *but to the mind itself* (Doran, 2015). The Sublime is that which could not be contained in any sensuous form, but rather speaks to ideas of reason itself. For example, the expression “I cannot express how much I love you,” by its very impossibility or failure, constitutes an expression of the love. *Kant knew that the only way to access essence is to create it through failing to access something* (the fall). Failing to depict essence is paradoxically essence itself.

## 19 Kant, Gödel, Bartleby, Hegel, and Žižek

Kant (1890) introduced a third category of judgment, i.e., *infinite judgment*. The purpose of this judgement was to explain concepts and judgements from pure understanding (Guyer and Wood, 1998), meaning concepts and judgements that do not arise from perceptions or the empirical senses. Pure judgements of this kind, of the pure understanding, are logical forms of judgements, as pure concepts, which are the logical categories. In terms of *quality*, there are two forms, namely, *affirmation* and *negation*. One can explain this using zombies. One can affirm a predicate, an example of which is that *something is alive*. One can also negate the predicate, meaning that something is *not alive*—being dead. The infinite judgement, on the other hand, is the in between of affirmation and negation. The infinite judgement is the *affirmation of a non-predicate*. Something can be alive, or it can be dead, or it can be *un*dead (a zombie). What is affirmed in the latter is a non-predicate, being the “un.” Thus, Kant toppled the traditional binary of affirmation and negation by introducing this third, in-between category. This category is precisely defined by the way it cannot be defined, and it does not require any sensory/empirical information.

The “un” is important, and it relates to the German language. In English, we typically understand finite as being the opposite of infinite. However, this is not the case in German, which is dialectic in nature. The German word for infinity is “*unendlichkeit.*” The “un” predicate indicates an uncertainty from within the subject (*endlichkeit*). Unlike its English counterpart, the German concept of infinity does not describe something endless—it describes the concept of the finite as something which contains within itself its own negation. This self-negation then creates something else. It is similar to the Freudian concept of “*unheimlich,*” which is translated into uncanny (Freud, 1919). This term means that which undermines itself from within. The German infinity thus describes a negation of negation, which is the affirmation of a non-predicate, or the Kantian infinite judgement. *Infinite judgements are Sublime.*

Turing’s halting problem and Kurt Gödel’s incompleteness theorem are of relevance. In brief, Turing demonstrated that computers cannot produce completely self-referential statements about themselves; computers cannot reveal truths about all computer programs. Gödel then demonstrated that mathematical systems do express truths about their own logics; mathematical systems express *what they can and cannot prove.* This is only true for mathematical systems based on computational logics. Hence, if axioms are computable, according to Gödel’s theorem, mathematical systems *cannot simultaneously be consistent and complete*. Hence, a computational system can only (1) prove a false statement or (2) the computational system would fail to prove a true statement. Hence, all computational systems would contain true statements, which it cannot prove. If the system does prove the statement, then the system is proving that the statement is false (Scientific American, 2006). Hence, computational logics can only (1) *fail* to prove a truth or (2) uncover that the truth itself is of a *failure*. Most importantly, the Gödel theorem demonstrated that mathematics and numbers are just quantitative measures, which would otherwise not exist without a quality to which they could attach. In other words, mathematics is derived from quality (or identity) and not the other way around (Naidoo, 2023b).

*In Bartleby, the Scrivener: A Story of Wall Street* (Melville, 2011), a clerk is hired by a wall-street lawyer to perform administrative tasks. After a bout of hard work, when the clerk is asked to perform another task, the clerk responds with “I would prefer not to.” This refusal then results in the workplace being sent into disarray. As noted by Žižek (2008),

“Sometimes doing nothing is the most violent thing to do.”

Although Bartleby *does nothing*, this nothing is destructive and turns out to be much more effective than doing “something.” For example, corruption is commonly understood as a hidden act. Most commonly, however, corruption involves hiding the fact that *nothing has been done*, as opposed to something. Corruption is an *intentional negligence*. As Žižek pointed out, “I would prefer not to” is an example of the infinite judgement, which would take the form of a refusal to accept false given, false dichotomies (as ideological oppositions), or choices. The use of “I would prefer not to” is, thus, a strong form of destructive autonomy, which highlights the self-undermining truth of all logical propositions.

## 20 The owl of Minerva

“When Philosophy paints its grey in grey, a shape of life has grown old, and it cannot be rejuvenated, but only recognized, by the grey in grey of philosophy; the owl of Minerva begins its flight only with the onset of dusk” (Hegel and Woods, 1991).

Hegel’s argument here is that philosophy (rationality, reason, and coherence) *occurs only after* the occurrence of events. Rationalizations, as stories, narratives, or explanations, are those created by implementing causality on events and information. These rationalizations are of the past, but they form part of the present, and they proceed forward into the future, unless that presupposed rationalization is altered. This reduction in the dimensions of information is necessary for efficiency (biasing) purposes for processing. “Un” information requires a higher energy expenditure to compute; hence, memories perform abstraction processes to create efficiency. *Memories are modes for theorizing about the world*, with explanations following this process of theorization. These narratives require impositions of biased causal and logic links, thus creating relations between informational points—creating a logical sequence (Taleb, 2007).

## 21 An open future and “the end of history”

“Men make their own history, but they do not make it as they please; they do not make it under self-selected circumstances, but under circumstances existing already, given and transmitted from the past.” (Marx, 1852).

Meta-freedom, as Naidoo (2023a) noted, is qualitative freedom, which is not simply the freedom to choose between a selection of presented choices but to construe the concept of freedom, or freedom of choice itself, differently. A degradation of meta-freedom, or qualitative freedom, is exemplary of an unhealthy, totalitarian, static, risky, closed, dead, and deterministic society/system (including a mind). In other words, this is an efficient society. Imagination, as Naidoo (2023a) noted, is lacking in closed and efficient systems, as there is no place for autonomy, contradiction, differences, or freedom of thought/expression.

Hegelian teleological historicity has often been mistakenly criticized as being closed, or leading to totalitarianism (Popper, 1945; Naidoo, 2023a). The idea of any pre-determined teleological unfolding of history typically gives rise to naïve notions of strong determinism, a lack of free will, and thus, a lack of autonomy and freedom. The notion of fate is thus always attached to determinism and unfreedom. However, as Hegel (2010) have pointed out, both freedom and free will are only possible *because* of fate, not in spite of fate. Free will is not a concept that describes being able to do what one wants. Properly construed, free will is an inverse (counterfactual) relation, which is contingent on alternative histories. Free will requires a situation, wherein there is an exact copy of the actor and their universe elsewhere. The actor is free, only insofar as they can enact an outcome, which their corresponding copy, in the alternative universe, could not do. In other words, they follow another path to their copy. Free will thus depends on the degrees of meta-freedom (Naidoo, 2023a), which one would counterfactually not have if one did not have free will.

It is not that we must imagine that Sisyphus can do what he wants; we must imagine that Sisyphus *wants what he does*. *Freedom lies in the narrative interpretations that people create*. Fate is the enabling condition for both the concepts of freedom and free will because both concepts can only have meaning if they are contrasted to another concept as their contradiction being fate. Hence, without the concept of fate, there can be no freedom or free will.

Any historical process contains within it the overlapping of necessity and contingency. However, as Hegel (2010) demonstrated in his extrication of contingency and necessity, it is not that an underlying deeper necessity is realized through a set of contingent actualizations. It is, instead, the contingent actualizations that determine the fate of necessity itself. This means that necessity, as a concept itself, only arises through retroactive interpretation of contingent events. This is how subjective narratives are formed by the brain, which are semantic relations between events and facts, which are biased toward the observing subject (Naidoo, 2023a) (see “Sedimentation and memory” in Supplementary Material).

Thus, true freedom (and openness) is, for Hegel, the ability to change previously accepted presuppositions because all presuppositions are groundless (Naidoo, 2023e). As such, all presuppositions are intrinsically open to change and re-interpretations through the processes of self-referential reasoning and abduction (Naidoo, 2023f) (see also “(Self)entrenchment and flexibility” and “Temporality and generative entrenchment” in Supplementary Material). A history that is not open to re-interpretation, or stagnant, is one that is dead. For example, an interpretation of a work constructed in 2023 *must be different* to an interpretation *of the same work* constructed in the 1950s. For any future to be open, it is necessary for any past narrative to be subject to a modern re-interpretation of society.

Freedom is the ability to constitute and interpret (or negate) events, which is to write and re-write events (Naidoo, 2023e). Information, which is repeated, surprising, or recent tends to be prime for storage and usage because this enables the brain to better predict later temporal occurrences (Hawkins and Dawkins, 2021). Recalling information involves the recollection of subjectively imposed narratives of events, which, upon each recollection, are slightly altered (Taleb, 2007). Charles Baudelaire was the first to theorize this, where he compared our memories to palimpsests that could be written and re-written on continuously. This has neurochemical backing—when new memories are formed, the brain actively “breaks” DNA to store the new memories. Alzheimer’s occurs when the repair process degrades that would “fix” this (Miller, 2021). This is a physical and violent re-ordering of the past (see “(Self)entrenchment and flexibility” in Supplementary Material).

Neurobiologist Robert Sapolsky demonstrated that there is fundamentally *no biological difference between love and hate*. Alertness is a function of the amygdala in conjunction with other regions. The amygdala activates a part of the brain stem (1) called the *locus coeruleus* (LC) (Breton-Provencher et al., 2021), which is like the brain’s very own SNS. This sends norepinephrine projections throughout the brain—including the cortex. If the LC is not “excited,” then the human is calm and unalert. If it demonstrates high activation, then this is a massive state of alertness in which *perception is amplified*. Importantly, this means that *the autonomic emotional patterns influence the intensity of feeling*/state, *but it does not determine the content of what one feels*. Both love and anger (positive and negative) work fundamentally in the same way, i.e., heightening or lowering feeling. If I “love” something very much, I tend to have a state of high alertness for that something, whether it is a person or observing the color blue of the sky. If I hate something equally, like the blue sky, I will have the same state and intensity of experience. As Sapolsky (2017) recalled, *the opposite of love is not hate, but rather indifference*.

Oxytocin is vastly considered to be the “love” drug (or chemical, to be more accurate). However, despite its benefits, there is also a negative side to oxytocin (Azar, 2011; Northwestern University, 2013; Badcock, 2016). Although oxytocin assists in the formation of mother–infant and monogamous pair bonds, lessens anxiety and stress, increases trust and social affiliation, and causes people to be more cooperative and generous, it only enhances pro-sociality towards the “us.” Oxytocin presence in interactions with “them” causes ethnocentricity and xenophobia. That which fosters love and sociality is also that which *causes divide. It is not dissimilar to how we justify war under the auspices of peace.*

## 22 Todestrieb: saving the death-drive

The Freudian notion of the death drive has a long tradition of being misconstrued as a literal death drive. However, Freud’s *todestrieb* described the view that man held about death, being something, which is staged, within life. The death drive describes the process of de-sublimation or de-subjectivization. This development, or ones *becoming*, occurs through this process of constitutive negativity or the Hegelian negation of negation (self-relating negativity). This is a pure form of agency, whereby one’s unfolding is a process of incremental “deaths,” which allows for development and the occurrence of heightened feelings (Naidoo, 2023b). Freud described that feeling humans obtain when closest to death (like rollercoasters).

There are four propositions that are important when considering the death drive (Hook, 2016): (1) biological instinct; (2) a cosmic principle; (3) the Nirvana-like release of tension; and (4) the impulse to self-annihilation. On (1), Žižek (1989) noted that

“[W]e have to abstract Freud’s biologism: ‘death drive’ is not a biological fact but a notion indicating that the human psychic apparatus is subordinated to a blind automatism of repetition beyond pleasure-seeking, self-preservation, accordance between man and his milieu. Man is – Hegel dixit – ‘an animal sick unto death’, an animal excoriated by an insatiable parasite (reason, logos, language). In this perspective, the ‘death drive’, this dimension of radical negativity… defines la condition humaine as such…. All ‘culture’ is in a way a reaction-formation, an attempt to limit, canalize – to cultivate this imbalance, this traumatic kernel, this radical antagonism through which man cuts his umbilical cord with nature, with animal homeostasis.”

The above statement is a Lacanian proposition that can be found in the Seminar on *The Purloined Letter* (Lacan, 2006), wherein Lacan equates the death drive with a form of symbolic constitutive repetition (automatic repetition). Thus, the death drive is not a biological instinct to return to pre-life (inanimation). This repetition is a form of obsessive compulsive disorder. In his 1964 seminar *The Four Fundamental Concepts of Psychoanalysis*, Lacan (1979) highlighted how the death drive is inherent to the Freudian unconscious and memory (Hook, 2016). The unconscious, in this way, is not able to satisfy itself other than by re-finding an object that has forever been lost to it (Hook, 2016). Thus, for Freud, repetition was not something *determined by humans but something which determined humans.* Repetition is an instance of “more-making” that serves to preserve stability (Eldredge, 2015). As a context-independent constraint, repetition increases the magnitude of a value within state spaces. Large increases in magnitude correlate to an increase in density, which can then *deform a state space*, thus potentially driving systems even further away from equilibrium and thermodynamic heat death. Repetition is also an insurance mechanism that ensures that valuable traits are not lost when perturbations occur, like the repeated nucleotide bases in the genome (Juarrero, 2023). This is the process of creating redundancy. Redundancy ensures that there are additional components in a system, thus introducing “fail-safe” measures, which preserve systematic functionality, even if individual components fail (rendering systems *robust*). Repetition is also important for communication as it improves the fidelity of transmission within noisy mediums as repetition communicates information relating to regularity, which is rare. Thus, repetition and redundancy preserve and transmit information and keep systems further away from equilibrium while preserving coherence and metastability (Juarrero, 2023).

The Lacanian death drive involves *a death in form* (the mortification of the Symbolic) rather than in content (morality and death) (Hook, 2016). The death drive in this way compels the subject, using antagonism, to transcend from the natural (the animal or the human) to the denaturalized subject (culture, identity, and the symbolics). This also involves a fundamental denaturalization of human sexuality (agent like sexuality that is not aimed at reproduction) as opposed to animal sexuality (instinctual coupling) (Hook, 2016). In this way, the death drive is the reason why the human animal is denaturalized and *not subject to the normal course of evolutionary adaptation*.

In *The Selfish Gene,* Richard Dawkins (1976) coined the term “meme” that described a transmissible piece of information, which can be sociocultural or symbolic–linguistic units, which circulate among entities who can share and use these units (Johnstone, 2008). They (memes) establish boundaries, co-ordinates, and attractors in mental, physical, and social spaces (Juarrero, 2023). They serve to frame and bias cognitive possibility spaces, thus spreading mental and social attractors. They are contagious, in that they are easily socially transmitted. The more predominant a meme is, the more they alter social and mental attractors, thus reconfiguring mental and physical spaces (Juarrero, 2023), including cultures, languages, and laws. Memes co-evolve with their contexts, and they display an inertia to change, even when conditions no longer require them or when they may be harmful.

Exaptation, an idea coined by Stephen Gould describes situations where the original evolutionary function of a feature is different to its current usage (Gould and Vrba, 1982). Wings, for example, originally evolved to increase surface area, thus enhancing thermoregulation. Flight as an ability was only exploited by organisms after the evolution of wings. Hence, features can have different functions, or no useable functions in one context, but *new/novel functions can emerge* in other contexts. Dawkins also noted the concepts of “de-aptation” or “de-aption.” These concepts describe instances whereby a meme can override the interests of those who created them (or genetic programming) and can change aspects of its founder. Although memes can arise from genes/biology, they are not necessarily genetic/biological, and they can obtain an independence from the material substrate from which they arise—a transcendence of sorts. Memes can hegemonize the biological substance of humans (Johnston, 2008), subjecting them to “non-biological” or denaturalized structures. This kind of indirect adaptation is described in “Negation and prediction” in Supplementary Material.

It was Schelling (1802) who first described the Universe as biological. He realized that some things could not be derived logically but could only be narrated (Žižek, 2020). Schelling’s conception of the Real as the primordial drives is meant to demonstrate a move from *logos to mythos*. The ancient Greeks believed (Schelling too) that the orgasm is the height of human experience because it symbolized the unification of the Two into the One. This unification was thought to be the Absolute, or the perfections, wherein harmony is achieved (no conflict) and differences are reconciled. However, there is no harmonious unity, only a unity-in-difference (a failure to unify, or an antagonistic gap), which enables a true brush with the Absolute (see “Temporality and generative entrenchment” in Supplementary Material). This is constitutive of human sexuality (Žižek, 2020) in two ways. The first, as an expression of autonomy, is through denaturalized sexual interactions and the second is through the introduction of novelty. Sexual reproduction involves a vertical genetic transferral (not duplication) from organisms to their offspring and a horizontal genetic transferral between bacteria and unicellular eukaryotes (Juarrero, 2023). The latter introduces novelty through *contextual import*, which is a multiple realizable constraint (non-random) that results in an expanded possibility space, with novelty and variation. Through both vertical and horizontal mechanisms, *novelty and change* are introduced. Sexual reproduction thus introduces genetic shuffling, which preserves a species lineage while also introducing novel variants and combinations of traits (Juarrero, 2023). This allows for a larger set of traits and behaviors while remaining true to type (see “Sex and novelty” in Supplementary Material). What this means is that *it is antagonism, contradiction, and failure to unify that is raised to the level of the Absolute.* Love is the death drive in a dance of de-subjectification. It is visceral and violent, carving away at one’s past and an emptying out of one’s content to de-subjectivize oneself for the other. This is also why “love” is commonly known as a truth event of pure freedom/autonomy as it involves a subjective interpretation and self-entrenchment (see “(Self)entrenchment and flexibility” in Supplementary Material). In other words, *the death drive is not biological, but ethical* (Hook, 2016). This forms the starting point for solving the is–ought dilemma, which will be explored in proceeding works.

To prove Kant right once more, the PFC *silences* nonpathologically during states of orgasm, which produces incredible amounts of emotion (Sapolsky, 2017). Hence, it is through its own *desublimation (silencing) of the superego as the PFC and ultimate reason and logic do we experience orgasm as the site of the most heightened experience*. The orgasm, as the work of the death drive, enables a brush with the Absolute (Žižek, 2020).

On (2), the death drive is not to be understood as the conflict between two opposing forces, but as an inherent *blockage* of the drives (Naidoo, 2023a; 2023f). This blockage then creates the appearance of two opposing cosmic forces (Eros and Thanatos), whom need to be chosen among and reconciled for there to be harmony. This will be dealt with in future works but relates to the concept of *semantic closure*. The death drive is the inner inconsistency of the psychical apparatus, *a constitutive gap that distinguishes drive from instinct* (Hook, 2016).

“There is only one drive; and the libido which aims for enjoyment and the death drive is the curved space of its formal structure” (Žižek, 2010; Hook, 2016).

As the drives involve blind psychically repetitive behaviors in pursuit of their own satisfaction (Freud, 1920), they obtain pleasure from beyond the pleasure principle. This is pleasure in pain; humans *seek out pleasure beyond the pleasure principle, i.e., in pain* (Freud, 1920). In other words, this is excessive or surplus enjoyment that links to (3). Going beyond the pleasure principle involves developmental excesses, which arise when systems are antifragile (Taleb, 2004). Antifragile systems are those that benefit from failure by incorporating failure into their constitutions. These are “safe-to-fail” systems, as opposed to “fail-safe” robust systems. Antifragile systems can self-ratchet and self-modify through self-reference, thus ensuring their stability. *Post-traumatic growth* in muscles perfectly describes this (see “Temporality and generative entrenchment” in Supplementary Material). Importantly, the concept of the death drive is fundamental to non-linear distributed networked communications, which will be explored in further work.

In terms of (3), the death drive is not the nirvana principle that seeks equilibrium or balance. Rather, it is *a mode for possibility* (Hook, 2016). The death drive is a mechanism of *expressing autonomy*. Properly conceived, the death drive then is not a compulsion to return to the void of pre-life that can be obtained through death; it is a movement in the opposite direction. It is the movement to lifeless life (the undead), also known as *the Kantian infinite judgement* (this judgement involves the affirmation of a non-predicate, like the undead, for example). *It is a movement away from death and toward immortality* (Hook, 2016). This is the source of human enjoyment, being the seeking and obtaining of surplus. Surplus enjoyment is something that is beyond repetitive biological life. It is a break with repetition, which is more than both life and death. *It is an excess of life*. *Jouissance* is the excess of pleasure that there is in pain, the perverted pleasure of the pain of repeatedly missing one’s goals (a misrecognition, or a *failure to unify/complete*) (Žižek, 2000; Hook, 2016). Importantly, in linking the death drive with the unconscious, Žižek noted that

“The unconscious intervenes when something ‘goes wrong’ in the order of causality that encompasses our daily activity: a slip of the tongue … a failed gesture … However … psychoanalytic interpretation does not simply fill in this gap by way of providing the hidden complete network of causality that explains the slip: the cause whose “insistence” interrupts the normal functioning of the order of causal is not another positive entity … it belongs rather to the order of the nonrealised or thwarted … that is in itself as a gap, a void insisting indefinitely on its fulfilment … The psychoanalytic name for this gap, of course, is the death drive, while its philosophical name in German Idealism is “abstract negativity,” the point of absolute self-contraction that constitutes the subject as the void of pure self-relating” (Žižek, 2005; Hook, 2016).

This intervention (slippages in causality or where there is a misprediction or misrecognition) enables consciousness to arise (Hawkins and Dawkins, 2021). Importantly, Žižek highlighted the unconscious intervention as being a non-realized or thwarted gap instead of being a positive entity. This is not dissimilar to the simulative processes in the PFC. Undesirable outcomes that do not exist other than mere probabilities as alternative histories are thwarted. Decisions are made based on the thwarting of undesirable outcomes based on non-existing probabilities (not dissimilar to the collapse of the wavefunction [see Quantum theory in Supplementary Material]).

In terms of (4), the death drive is the movement both toward immortality as I mentioned above and the movement toward the destruction of the *metaphysical conception of immortality* (as life that persists beyond death) (Hook, 2016). Thus, it is the destruction of life, but not the destruction of that which is in life, more than life itself. It is a movement against moderation, which is in one’s best biological interests. This is a form of self-relating negativity *a la Hegel*.

## 23 Conclusion: a brush with the Absolute?

A brush with the Absolute entails a brush with the explanation closure axioms of reality, which is a brush with a true *form of agency or freedom*. This is because explanation closure axioms specify (1) which events may/may not occur; (2) specification of which events can change a property (thus reducing the logic to simply reasoning about what events may or may not occur); and (3) axioms themselves encode solutions (however, not all solutions need to be coded within them, some can be generated in action). In other words, explanation closure axioms specify *that which they do not specify* (an incomplete Absolute). This is non-formulaic and enables representation, interpretation, and causation. Plan recognition includes instances whereby the pre-programmed epistemological axioms/assumptions “fail” in the sense that they did not predict an event that may have occurred or the solution is one that is generated on the fly through *acts of true freedom or agency* since these acts would be free from the formulism.

Importantly, these epistemological assumptions are evidence of pre-programmed *planning*. Both recursion and iteration are only possible after planning wherein temporal dependencies/sequences are formed and then used (Juarrero, 2023). Explanation axioms *do not need to encode for all events* like those caused by natural forces. This means that natural forces, as events, can interfere with the prediction process, and it would be *impossible* to prove that an event did not occur (a constraint). Kant described this perfectly when he painted a picture of the dynamically Sublime, which describes incredibly massive forces of nature that, when recognized in its representative form, does not threaten our lives, which instead fills us up with power of *resistance* to it (enables acts of freedom and heroism) (Doran, 2015; Žižek, 2020). In other words, *a failure of the plan produces an otherwise unprovable truth*. It may even be so that subjective interpretation *is itself a form of natural force, which alters the course of events*. This will be argued in proceeding works.

In this way, one can circumscribe the “existence” of a plan through a proof by contradiction. This was also the modality used to prove the most difficult mathematics theorem from the 17th century, Fermat’s Last Theorem. Andrew Wiles constructed a proof by contradiction wherein Fermat’s Last Theorem was proved to be unsolvable *and, thus, true* (Klarreich, 2020). Proofs of this nature (indirect proofs) establish the validity or truth of a proposition by demonstrating that if the proposition were to be false, it would lead to a contradiction. It is thus a proof by assuming the opposite of what one intends to prove as true—by relying on purposefully bringing about a contradiction. If the contradiction arises, the assumption is incorrect, and the conclusion is true. In this way, one can prove the existence of a kind of object, without providing an example of it (this is known as a non-constructive or existence proof) (MathWorld, n.d.). There are existing truths, which are not computationally identifiable (since they are epistemological). From the perspective of the planned for (or *plan*), it would be an instance of the Sublime, because there is a *lack/failure to obtain knowledge of the plan itself* (the epistemic constraint mentioned above). However, one can “identify” the contours, or existence of the plan itself, by circumscription (through failure or impossibility). Hence, the Absolute are the framing axioms of humans (our epistemology), and the brush with these axioms is a proof by contradiction of a programmer. Thus, truth is obtained through an identification of “failures” or “gaps” or “limits” of initial planning axioms (their ambiguity). The brush with the Absolute, as the failure to obtain truth, is constitutive for individual (as the collective human) autonomy or freedom, as Kant described. A plan, however, does not negate freedom; *a plan enables it*.

The discussions of the various epistemological ontologies, including the scientific ones, served to highlight the invariant cross-cutting theme of radical, intrinsic openness. Each thus demonstrates the intrinsic property of openness as being the ability to write and rewrite (determine, re-determine, constitute, and reconstitute) narratives, knowledge, and systems. In other words, one is always able to go back to the drawing board, or as Hegel put it, necessity is just an instance of actualized contingency posited retroactively. Rationalizations occur in hindsight, and rationalizations *must always remain* open to be re-rationalization. Thus, degrees of freedom are afforded by this radical openness, which enables freedom of subjective interpretation and, ultimately, *agency*. Building on this, a truly open society and system is one that is measured by its ability to re-write itself and whether it actively creates conditions where its components can *challenge/change* their own presupposed truths/rationalizations. This is *semantic/imaginative freedom*.

Unfortunately, philosophy got lost along the way. Philosophy is the study of thought, logic, and rationalization itself. Philosophy *requires* taking the impossible topological (external, or Bartlebyian) view. Philosophy *is* knowledge; knowledge *is* coherence; coherence *is* trust; trust *is* subjective order; subjective order *is* embodied health. Unfortunately, current times seem to separate philosophy from other sciences, forgetting that the natural/empirical sciences used to be called “natural philosophy” until recently. Remember, there is a philosophy of science, but there is no science of philosophy. This attitude toward philosophy, or critical thinking largely emanates from capitalism, and its fair maiden, the analytic and empirical sciences (like physics). The domination of the latter schools of natural philosophy requires that philosophy must always be attached to another discipline and provide “practical applications.” Practicality, properly speaking, is the *efficient cultivation of objects rather than persons*. Moreover, the schools of natural philosophy tend to repress the fact that they are philosophy, so they can repress other schools of philosophical thought and secure more resources (funding and primacy) for themselves at the cost of others (Naidoo, 2023a). The schools of natural philosophy would do well to remember that claiming a depoliticized, or objective zone, free from bias or subjectivity, *is the most political act*. Subjective ideology is at its strongest where it is universalized or not experienced as such. The most political act *is to claim a space of non-politics* (ultra-politics). In other bodies of work (Naidoo, 2023a; 2023b; 2023c; 2023e), it has been demonstrated that the logics underlying the empirical sciences come from the German Idealists, most notably, Hegel, who obtained such from Christian theology. This is a truth that must be rendered.

Implicit in the denigration of thought are the following assumptions: (1) thinking is not practical and (2) thinking is not valuable. In terms of (1), this is a false separation between theory and practice, arising from Kant (Naidoo, 2023a). There can be no practice without theory, and practice is blind without theory. All quantity is derived from a quality. This is the common mistake that mathematicians make as well; magnitude or counting cannot exist without having at first a *quality*, which is to be counted. Quality is difference or identity. Identity is theory, or a rationalization, or an epistemology, which is used as a framing device. Proper thinking is speculative processes, which thinks *quality and quantity together*; the *good and the bad are thought together*.

In terms of (2), the denigration of thinking as not being valuable unless it has a market application only serves to entrench terrible presuppositions rather than creating an environment where presuppositions can be tested, validated, and continually replaced. Viewing thinking and theorizing as not being valuable is harmful to the embodied health of persons and societies since it prevents both from actualizing novel rationalizations, which are more suited to remaining both viable and open. In societal terms, this attitude can lead to totalitarianism, pessimism, rigidity, and societal fragility. It is *contra-evolution*. Moreover, as mentioned, memories are modes for theorization. Thus, if meta-degrees of freedom for theorization are reduced, so too would be memory. The loss of individual or societal memory (semantics) is the reason why past mistakes are repeated and is a *sign of decline* (in terms of normal health, and especially embodied health). Memory is vital for understanding, creativity, production, and the continuance of life.

When one considers value in terms of a singular outcome, being a market application, one only serves to create a linear and nihilistic subject, which becomes a servant to a single truth; this truth is typically in the interests of someone other than the subject. This is the movement of efficiency, being a servant or a subject, of a single truth, instead of serving as many different truths as one wills. One cannot will different truths, if one is not able to construct different qualities. Value must remain ambiguous because this would enable persons to construct their own agency in relation to what is valuable to them. Ultimately, what is of *value to society* is the cultivation of individuals who can *think critically*. Critical thinking (*taking the topological view*) requires the use of metaphysics and meta-reasoning (Naidoo, 2023a), which is the ability to construct *qualitative rationalizations or new theories*. Why is this necessary? The importance of meta-thinking, or epistemological questioning, is that one can use it to ensure that a system/ontology remains open. In terms of health, the said methods allow one to critically question whether the ways in which problems are framed *actually reproduce those problems instead of solving them*. One needs theory to determine where practice is failing and to jury-rig new practices. This is also the importance of the violent act of love (or the death drive); it is love that is the enabling condition for rewriting one’s past, steering the course of oneself, or society, into a more optimistic direction when previous paths lead to failure. Of course, the denigration of love today can be traced back to natural philosophy (Descartes, in particular), which seeks to cut people off from their emotions and feelings, and the self-empowerment associated with being able to steer the course of one’s emotions. This happens only so that the schools of natural philosophy can maintain their entrenched positions.

Society today is one that could not produce valuable and creative thinkers, even if it willed. This is incredibly pessimistic for maintaining an open, liberal, optimistic, and free society. This is terrible for embodied health as is the only position critical thinkers are afforded today:

“Better to do nothing than to engage in localized acts whose ultimate function is to make the system run more smoothly (acts like providing space for the multitude of new subjectivities, and so on). The threat today is not passivity but pseudo-activity, the urge to “be active,” to “participate,” to mask the Nothingness of what goes on. People intervene all the time, “do something”; academics participate in meaningless “debates,” and so forth, and the truly difficult thing is to step back, to withdraw from all this. Those in power often prefer even a “critical” participation, a dialogue, to silence—just to engage us in a “dialogue,” to make sure our ominous passivity is broken” (Žižek, 2006).

Society tends to forget that people theorize and rationalize to make sense of the randomness, chaos, and suffering that surrounds them so that they may survive. In thermodynamic terms, they do this to reduce their entropies by creating order. People theorize to keep their futures undetermined, open, and optimistic. A healthy society is one that *provides resources* to people so that they may cultivate their own embodied health, as opposed to objects. As Kant noted, transcendence, or optimism, relies on *subjective purposiveness and not objects*. The human dopamine system supports this, wherein it is the subjective path, which is prime, not the object itself. Moreover, people simply enjoy and, thus, are optimistic about creating theory because it is the act of subjective purposiveness.

## Data Availability

The raw data supporting the conclusion of this article will be made available by the authors, without undue reservation.
