# Supplementary material for: Open optimism as an “embodied-health” ethic for the information era
Source: Front Pharmacol. 2024 Jun 17;15:1331237. doi: 10.3389/fphar.2024.1331237 (PMC11215117; doi:10.3389/fphar.2024.1331237)
Supplement: Supplementary file 1 [file DataSheet7.pdf]

frontiers

Supplementary Appendix

Open-optimism as an "embodied-health" ethic for the information era

1 Retroactivity and logic

Concluding his discussion on the Hegelian dialectic, Gabriel (2011) says this:

"Given that transcendent metaphysics conceives the absolute as the entirely other that transcends the totality of determinations, it cannot characterize it through any positive predicate. For this reason, the transcendent absolute is traditionally dealt with in terms of an absolute oneness or absolute identity which cannot positively be described, as this would make it something determinate and, hence, part of the world, part of the network of determinate beings. As Hegel has it, the simple substantial identity of the absolute is indeterminate, or rather in it every determinateness of essence and Existence, or of being in general, as well as of reflection, has dissolved itself. Accordingly, the process of determining what the absolute is has a negative outcome, and the absolute itself appears only as the negation of all predicates and as the void. It is obvious that the negation of all predicates cannot be a reflection performed by the negative absolute itself. Otherwise we would have to ascribe some sort of self determining activity to it, a move that would contradict its alleged absolute identity. Hence, it is our own reflection that accomplishes the negation of all predicates. However, this entails that the absolute is already determined in opposition to our reflection as that which does not accomplish the negation itself. This in turn implies that our reflection has merely been an 'external reflection' up to this point. Reflection opposes itself by positing an absolute: it posits the absolute as if it were not posited by reflection. Yet it is, hereby, already determined by reflection. This motivates a countermove. If it makes sense to talk about the absolute at all, we cannot define it in opposition to reflection, lest this opposition relativize it. Reflection must not 'stand over against the absolute identity of the absolute.' This is why the absolute has to be understood as the 'ground' of totality, with no determinate content but that of grounding. For this reason, the correct determination of the absolute has to be the 'absolute form,' which is in and for itself 'the absolute content,' as Hegel puts it. It is nothing but the name for the grounding relation, by which the finite becomes intelligible as such. Yet, this grounding relation does not have a content apart from that of being a grounding relation, it is no particular relation, which would hold between two relata and, therefore, between two entities. An absolute, which satisfies this prima facie weird condition can only be the movement of pure thought performed by the Science of Logic itself. The Logic itself is the unfolding, the exposition of the absolute. The absolute is both the form and the content of the Logic and is, hence, not something prior to its manifestation in logical thought".
