# Supplementary material for: Open optimism as an “embodied-health” ethic for the information era
Source: Front Pharmacol. 2024 Jun 17;15:1331237. doi: 10.3389/fphar.2024.1331237 (PMC11215117; doi:10.3389/fphar.2024.1331237)
Supplement: Supplementary file 2 [file DataSheet13.pdf]

Supplementary Appendix

Open-optimism as an "embodied-health" ethic for the information era

1 The brain

1.1 The old lizard brain

The old brain, known as the autonomic nervous system, is responsible for maintaining regulatory bodily functions, hormone release, and homeostasis. Importantly, this region (the brain stem and mid-brain) is responsible for stress responses. It contains the sympathetic nervous system (SNS) and the parasympathetic nervous system (PNS) (Alshak and Das 2022; Waxenbaum et al., 2022). The SNS is responsible for mediating responses to arousing stress related responses including fear, fight, flight, and sex (it releases norepinephrine and epinephrine—different kinds of adrenaline). The SNS is responsible for increasing heart rate. The PNS is about inhibition, calming (reducing heart rate), and vegetative states. Mark Solms has situated the old brain as being the seat of consciousness (Solms, 2017). This is because consciousness is about *affect*.

1.2 The limbic system

The limbic system (Rajmohan and Mohandas, 2007), which is more expanded in mammals, is responsible for emotions. If you "feel" a certain way, this region commands other parts of the brain to make changes. It can also initiate stress responses. In primates, including humans, the limbic system tends to be dominated by visual inputs, but other inputs have influence too. This system includes the amygdala, hippocampus, septum, habenula, and the body. There is no central or unificatory part of the system which processes emotion, and its structure is highly interconnected and complex. It works via an excitation and inhibition process. The limbic structures mainly influence what the hypothalamus does.

1.3 The hypothalamus

The hypothalamus is the interface between: (1) the old brain; and (2) the limbic system (Saper and Lowell, 2014)—it is responsible for emotional regulation. As it is part of (2), it gets massive input from (2) and sends a lesser amount back to (1)—in doing so, it influences (1). Emotions change bodily functions, thought, desires, and so forth. There are many vital functions of the hypothalamus, including the regulating hormone release (Sapolsky, 2017). So, the limbic system influences hormones and autonomous activity, both of which are fed back to the body, and then back to the brain—this influences both thoughts and behaviors. The limbic system also influences (3) the cortex. While a lot of sensory information is decoded within the cortex, there is no actual separation or hierarchy between both—they have a dialectic relationship, with each one talking to, and influencing, the other. When Descartes separated both, he was mistaken, as pointed out by Damasio in *Descartes Error: Emotion, Reason, and the Human Brain* (Damasio, 2005).

1.4 The amygdala

The amygdala is part of the temporal lobe and plays leading roles in regulating aggression, anxiety, and fear. When the amygdala receives certain "fear inducing" stimuli, it commands that behavior

expressing fear to be enacted. This region also processes information which comes in at a subliminal level (too quick or too small for the other regions, including the cortex, to process) (Sapolsky, 2017).

Importantly, the amygdala is responsible for both learned and “innate” fears. Innate fears are complex, but it is understood to be a more akin to a “prepared learning” approach to innate fears, meaning that it is easier to learn to fear (X) as opposed to (Y) (Sapolsky, 2017). This includes a predisposition to fearing certain appearances and contexts (when the hippocampus is drawn into the framework as described below).

This region is also vital for social functions—namely, in situations where there is uncertainty. It plays a large role in social and emotional decision-making. Decisions to reject something or someone are routed in a desire to “punish”. It works in an interesting way—the human natural state is to trust and be altruistic. However, when there is a negative social experience, the basolateral amygdala (BLA) activates and injects a change in the amygdala. This is to have distrust and vigilance in social interaction into the amygdala.[1] Decisions in social environments tend to be emotionally based and are driven by rewards. The rewards are either short-term or long-term. If the payoff on offer is poor, or does not result, this results in anger and rejection. Interestingly, experiments have shown that *the amygdala does not seem to activate when it is a computer which offers a poor payoff instead of another human as there is less vigilance and more focus on maximizing long-term rewards* (Sapolsky, 2017; Adolphs et al., 1998). The amygdala is most responsive to positive stimuli when the value of the reward is shifting. The neurons in the BLA also respond in the same way when something “bad” is happening. These neurons are more concerned with “changes” regardless of whether it is an increase or decrease in reward. Thus, shifts in rewards and punishments have the same affect. The upshot of this is that *the amygdala is not about experiencing pleasure per se—it is rather about the uncertain and yearning for a potential pleasure*. It is also concerned with anxiety, fear, and anger—if the reward may not happen or is smaller than expected (Sapolsky, 2017).

The amygdala is also responsible for dealing with pain. It receives information regarding a trigger of fear and aggression—which is pain. This is mediated by the *periaqueductal gray* (PAG), which is part of the old brain structure. In line with the above, it is *unpredictable or unpredicted pain*, rather than pain itself, which activates the amygdala. Thus, the context is vital—if the brain makes a prediction and there is an unexpected trigger, then the amygdala activates to condition and learn a fear. This is how learning works—both in infants and in conditioning a child for learning within a specific environment (like a classroom). The conditioning does not need to be “physical” pain, as described below, the brain mixes up visceral pain with psychical pain—which results in the same activation and conditioning (Sapolsky, 2017).

Another important aspect which contributes to my arguments is the role that the amygdala plays in disgust. The amygdala receives information from the *insular cortex* (part of the pre-frontal cortex). When one experiences any form of sensory disgust, the insular cortex activates. Interestingly, this includes *thoughts of moral disgust*. Moral disgust can include the breaking of social norms. The disgust in the insular cortex activates the amygdala (Sapolsky, 2017).

As mentioned above, the amygdala can enact actions. It “talks” to other regions including the prefrontal cortex (PFC), insula, PAG, sensory regions, and hippocampus (including other limbic structures)—it modulates all of their *sensitivity*. The amygdala is concerned with “setting off alarms” in the brain and body. The central amygdala is the core of the amygdala, and projections from there

[1] This is evident in patients with a damaged BLA which then does not process negative experience resulting in the state of altruism remaining.

go to the *bed nucleus of the stria terminalis* (BNST). The BNST then projects to the parts of the hypothalamus which can initiate hormonal stress responses. It also projects to the midbrain and brain stem parts which active the PNS. Thus, if something with emotional *effect* happens, layer two (limbic) signals to layer one (lizard) and heart rate and blood pressure increase. This is vital for alertness (Sapolsky, 2017).

1.5 The basolateral amygdala

The BLA plays a huge role in innate fears. In mammals, the BLA has functions in both learning fear and innate fears. Innate triggers of fear—such as that of shock (shock does not need to be electrical; it is just “surprise” the brain experiences when there is an outcome different to its prediction). It therefore is *unconditioned stimulus* that causes shock. When there is shock, the amygdala activates, and stress hormones are released by the SNS (producing the freezing of the body). One can condition the stimulus by adding an un-innate fear (including a sound or a context like a classroom)—one can condition a fear-stress response correlation. This conditioning excites the neurons in the BLA, which are then remapped to activate the amygdala. Importantly, the neurons respond to the meaning of stimulus, not its modality (this means that it responds to the correlation/association of X with shock—X can be any modality). There are synaptic changes that occur too—the BLA neurons become more excitable (more receptors and spikes) and growth in the connections between the BLA and amygdala occur (Sapolsky, 2017). Nonetheless, fears can also be unlearned (via the PFC). *The PFC can break associations*, which is active learning. The BLA gets input from all sensory systems/organs—and some sensory information can *bypass the cortex*. If there is sensory information “like a shock” or “subliminal cues”, which occurs in the presence of more excitable neurons in the BLA, the BLA will then respond before the cortex can register this information. It will then feed it to the amygdala, as part of a process of emotional condition and fear learning, and it can create automatic behavior (through instructing the motor regions to act) before the cortex is aware. It is not as accurate as the cortex, and often makes mistakes, but this kind of ability and interaction can lead to harm from mistakes.

1.6 The hippocampus

The hippocampus is also fundamental for numerous reasons. It learns detached and unemotional facts, while the amygdala learns content—such as “fears”. As mentioned, the amygdala can recruit the hippocampus in fear learning. The amygdala will learn the stimulus cues and the hippocampus learns contexts in which those cues arise. The hippocampus remembers contexts to the extent of excitation in the amygdala (and the BLA which conditions it) (Sapolsky, 2017). The hippocampus is responsible for learning models of the world. But how does it create models of the world? It does so through its use of grid cells. These gird cells create a conceptual map of the world (which is then stored in memory) that allows movement and orientation in the world. The grid cells create a reference frame. However, the content of the grid cells are filled in by the cells in the entorhinal cortex (EC).

1.7 The entorhinal cortex

The EC contains the place cells which give content aspects to what is found at locations. The combination of both place and grid cells not only map the environment, but they track the body as well (Hafting et al., 2005).

1.8 The prefrontal cortex

The PFC is one of the four folds of the cortex.[2] It is understood to be the interface between the limbic and the cortex layers—it is a member of both, despite being the most recently evolved layer of the cortex (Nauta, 1971). They both simulate, inhibit, collaborate, and co-ordinate one another. The PFC is the "decider" in executive action. The choice among options is made with reference to cognition and emotions. Once a decision is made, the PFC sends projections to the frontal cortex (FC) which then sends projections to the motor cortex, thus causing behavior. It functions *to do the right thing when it is the harder thing to do* (Sapolsky, 2017). This means the ability to enact thoughts and actions in accordance with internal goals (reward function). The FC is also responsible for task focus—including abstractions such as rule learning, pattern abstraction, correlations, rule applying, and rule changing (strategic action in terms of rules). It is also responsible for grouping or categorical thinking—like semantic grouping. Importantly, this FC is a highly energy demanding region, and it is also subject to high metabolic rates (Sapolsky, 2017). After periods of intense PFC activation, there is also a decline in performance from other regions. Once a rule is internalised, it gets stored in the *cerebellum* which makes it activation automatic/reflexive. The PFC also unlearns rules by inhibiting the BLA.

1.9 The cortex

1.9.1 Structure

The cortex is the most recently evolved region of the brain, as well as the biggest—occupying around 70% of the brain's volume (Hawkins and Dawkins, 2021). The cortex is often associated with the seat of higher intelligence—including conceptual knowledge. The connections of the neocortex are incredibly complex. It is connected to the old brain; this connection enables the enaction of movement when "wanted". Both regions co-operate through talking to one another. It is also a fact that functions and processing of the brain *is not top-down or hierarchical*. There are also many other facts about the neocortex which contradicts some of the traditional misconceptions surroundings its place and functionality.

1.9.2 Predictions

There are no specific sensory or motor regions per se—it is all interconnected (Hawkins and Dawkins, 2021), or dialectic. All regions look almost identical, indicating that they work in the same way. Vernon Mountcastle (1997), the father of modern neuroscience, proposed that: (1) the new brain is just a product of evolution making copies of an already existing principle of the brain; (2) this principle is that of a cortical algorithm (a simple circuit that explains all brain behavior)—the unit of that cortical algorithm is known as a cortical column, which is also the unit of intelligence; and (3) it is not the region that controls the organ of perception, it is the perception that gives function to the region—each cortical column is divided into mini-columns (Mountcastle, 2009). Each column is responsible for prediction (Hawkins and Dawkins, 2021) in all perceptions, experiences, and thought/learning, including at the most minute level (other than subliminal). These predictions are based on experiencing "objects" and "subjects" in the world. Including predictions based on conceptual information which can be acquired from other subjects (Hawkins and Dawkins, 2021). These predictions *never stop*. In the event of a misprediction, a stress response activates to *possibly* correct the built model and enhance prediction accuracy. This is followed by conscious awareness (described below). *Learning/prediction correction occurs prior to conscious awareness.* Conscious awareness is just a heightened perception caused by stress and anxiety. It is possible to argue that consciousness is not the modality of correction, but rather just the affect result of failure which brings

[2] The other three are the temporal, parietal, and occipital.

about a heightened stress response. The ability to learn a predictive model comes from physical movements in both the world and the body moving relative to the world (Hawkins and Dawkins, 2021). However, *predictions are also based on conceptual movements*. Thus, the only way to learn is for there to be a continuous *change in inputs* (known as sensory input learning).

1.9.3 Reference frames and prediction

The neocortex creates references to model and make predictions for objects and the body. The reference frames include "location on object" and "location of body relative to object" (Hawkins and Dawkins, 2021). There are multiple reference frames for each sensation and each minute part of the body/object that is being sensed. This means that there are several predictions happening in the brain at once (feeling, seeing, smelling, and so forth). This also means that the brain does not process pictures. Instead, each patch of retina takes in a little information forming a picture—which is then broken up into smaller pieces of information in order to form reference frames for objects and distance of body to object.

The function of the neocortex is to *process reference frames and not sensory input*. The cortex associates sensory input with reference frames. Reference frames are used in robotics to plan movements (Hawkins and Dawkins, 2021)! The universal cortical algorithm that underlies all brain function is reference frames (Hawkins and Dawkins, 2021; Hawkins et al., 2017)! The key to reference frames is the EC and hippocampus. Evolution works with what it has - in the brain this is called "neural reuse" (Ptak et al., 2021). Grid cells, place cells, and head direction (orientation) cells found in the old brain must be present in each cortical column in the cortex (Hawkins and Dawkins, 2021; Hawkins et al., 2018; Lewis et al., 2019). Functional magnetic resonance imaging (fMRI) experiments have shown strong evidence for the presence of grid cells in the neocortex (frontal region) (Doeller et al., 2010). Another experiment confirmed this and took it further, demonstrating that the neocortex stored visual images of birds in map-like reference frames. They also demonstrated that when subjects were thinking of birds, they were "moving" through these maps (Constantinescu et al., 2016).

What does this mean? It means that *thought is simply moving between reference frames*, and that this is how higher-end conceptual knowledge is built, organised, and stored. *This is sequential—and not random*. Thinking is a form of movement between differing frames which can have different content or different viewpoints on the same matter.

Not only do reference frames store information, but it is likely that they store other reference frames too—like a hyperlink (Hawkins and Dawkins, 2021)! This also solves the problem of *unification of perception*; it is not that the brain has a central region where all these different models of the world, self, and objects are combined—rather information is spread out everywhere. The brain *stabilises all different perceptions and unifies them through voting*. The perception one has is the result of a vote. Each column likely votes (Hawkins and Dawkins, 2021). The voting theory has been around for some time, but it did not attract much attention because it was incompatible with the older views of the brain. However, this model solves those issues. Each column can be uncertain, given that there are long range and interconnections abound, all this information will be passed around until the most common "guess" is settled on—being one's perception (Hawkins and Dawkins, 2021). This happens very quickly, and it requires learning (the infant development phase). As Hawkins and Dawkins (2021) note, this is also why perception, such as sight, is stable despite eyes continually saccading.

Higher-end cognition (reasoning about non-experiential things), such as abstract concepts, takes place within the neocortex and it does not rely on experience per se to reason. Nonetheless, the limbic

system influences the thoughts and perceptions processed in the cortex. The false dichotomy between thought and feeling was demonstrated by Damasio (2005). Thought and emotion are inseparable.

1.10 The frontal cortex

The FC is primarily responsible for executive function. This involves organizing knowledge in a strategic manner and then enacting action based on an executive decision. Additionally, it oversees gratification postponement, long-term planning, regulation of emotions, and impulsiveness control (Sapolsky, 2017). Importantly, the FC is the last region of the brain to fully mature—with the PFC's full maturation being last since it was the last region of our brains added in evolutionary time. The FC is not fully mature until the mid-twenties (Sapolsky, 2017). The difference I already mentioned between humans and other beings, including flies, is not qualitative but quantitative. The only exception to this is a neuron called the *von Economo (VE) neuron* or spindle neurons. However, these are also found in other primates, whales, dolphins, and elephants (Sapolsky, 2017). These neurons are only present in two sub-regions of the FC—the insula (disgust) and the anterior cingulate (responsible for empathy) (Sapolsky, 2017).

2 Unconscious processing and priming

Our brains are primed by cues in external environments and cultures. These include visual, auditory, smells, and touch. Each cue communicates information to us. The predominant sense in humans is vision—however, sounds and smells have an impact too. They also have an impact on our limbic system (Sapolsky, 2017). Thus, we know sounds, smells, and the rest bring up feelings in ourselves. In humans, subliminal or unconscious cuing alters the brain—including voices and interactions. Many of these cues escape our conscious or cortex grasp—but not the amygdala. Thus, these sensations shape brains and behavior in an unconscious manner. To recall, the amygdala processes sensory information. Most sensory information goes through the thalamus and then is processed by the cortex. However, the shortcut from the thalamus to the amygdala allows for sensory information to be interpreted and behavior stored or enacted much faster.

The brain also processes information about internal states—such as hunger and cold. This is interoceptive information (Kleckner et al., 2017). These also influence our behaviors and thoughts. In humans, we have atrophied olfactory systems (sense of smell). Our olfactory systems are unconscious, and they have the most direct access to the limbic system. For example pheromones are secretions which communicate internal states between members of the same species (usually). We can smell fear, anxiety, and anger although to a smaller degree than other mammals. There is more amygdaloid activation with smells. In infants, olfaction aids in identifying the mother, which is the source of survival—namely nourishment, warmth, and everything else that an infant needs. There have been many studies demonstrating this relation-approach (Setterberg, 2017; Etherington, 2020; Bowlby, 1962). Other studies in mammals have demonstrated that infants do not release glucocorticoids when the mother is around (Sapolsky, 2017).

3 Interoceptive information and emotions

Interoceptive information plays a massive role in determining our emotional state. Emotions are the physiological state that the body is in—it speaks to an intensity of a state, such as fast heartbeat and high blood pressure. Behaviors reflecting emotional states include aggression, reactions to pain, or calmness. These are different from *feelings*—described below.

Interceptive information is processed by the PFC, insula cortex, anterior cingulate cortex, and amygdala. It refers to information about the internal state of the body (such as hunger) or information from perceptions which do not first acquire recognition in consciousness (but which consciousness can pick up later). Our *feelings* (and behaviors) are based on interoceptive emotion processing, which is automatic (Sapolsky, 2017).

Importantly, interoceptive information does not only influence the brain, but it can also be influenced by the brain. The brain can alter sensitivity of the senses—either making them more sensitive or blocking them out in varying degrees. It can also make certain stimuli more important than others. When you are stressed (this is called eustress (Bienertova-Vasku et al., 2020)), you become better adaptive to your environment and can concentrate/focus/heighten awareness—thus, your ability to perceive is amplified. For example, if you are hungry, your sense of smell if amplified. On stress, we tend to confuse stress with psychological states. All stress is, is a means of enhancing perception (acute stress—chronic on the other hand is damaging). Stress is not a *feeling*, it is a bodily state, and distress is an example of a behavior or mental state which reflects the bodily state.
