# Supplementary material for: Open optimism as an “embodied-health” ethic for the information era
Source: Front Pharmacol. 2024 Jun 17;15:1331237. doi: 10.3389/fphar.2024.1331237 (PMC11215117; doi:10.3389/fphar.2024.1331237)
Supplement: Supplementary file 3 [file DataSheet2.pdf]

Supplementary Appendix

Open-optimism as an “embodied-health” ethic for the information era

1 Sedimentation and memory

Markovian mechanics, such as Newtonian mechanics, are those which are presumed to not possess any memory. These are *path-independent* mechanics, which involve a linear movement towards thermodynamic equilibrium. However, complex, self-organizing, and context-dependent systems are *path-dependent*. Path dependencies are forms of temporal constraints (generative constraints), which enables for the formation of complex forms of memory, called *records or registers*. Interdependencies which are constructed through contextual constraints generate path-dependent dynamics. These are forms of memory (Juarrero, 2023). The dynamics and structures of these interdependencies, and their correlative dynamics, are the embodiments/records of the systems *contexts, and the systems histories*. This means that aspects of the systems histories are integrated/intrinsic into/to their current constraint regimes, and hence, their constitution. In turn, this means that the histories of a system can influence their current constraint regimes—known as the *ability to remember* (Rovelli 2018). Sedimentation is thus a form of memory—which aids in sustaining coherent organizations.

Hence, historical-factual information persists in those records, and those records then influence the present, as sedimented constraints (Juarrero, 2023). Memories are thus records of real constraints, and if said memories are incorporated as governing constraints, they can *affect possibility spaces*. Memories have *weight*, are continuously updated, and they (memories) *bias future events*. They also serve to stabilize constitutive constraints. They can be harmful when they hinder the ability to reconfigure any existing constitutive constraints (considering new information) (Juarrero, 2023). History thus does *indirectly* influence the present—but *history does not determine* the present, because of the effects of multiple realizability and top-down control (Juarrero, 2023).
