# Supplementary material for: Open optimism as an “embodied-health” ethic for the information era
Source: Front Pharmacol. 2024 Jun 17;15:1331237. doi: 10.3389/fphar.2024.1331237 (PMC11215117; doi:10.3389/fphar.2024.1331237)
Supplement: Supplementary file 4 [file DataSheet4.pdf]

Supplementary Appendix

Open-optimism as an “embodied-health” ethic for the information era

1 Freud

1.1 Thresholds and consciousness

Freud was influenced by his predecessors who were trying to establish when a perception crosses a threshold and enters consciousness. This threshold was linked to the intensity of the perception (like burning candle away in the distance, you can see it as it comes closer to you only). Thus, the question at the time was how much quantity of perception (or energy) was needed to cross a threshold into consciousness which is a qualitative experience. In this light, Freud thought something similar must happen with the unconscious and the consciousness. There must be a threshold in which stimuli awareness in unconscious processes become subject to consciousness.

When Freud (1915) published his breakthrough work, *The Interpretation of Dreams*, wherein he proposed that this threshold was not a stimulus threshold but rather a defensive threshold. Freud was suggesting that the psyche was trying to maintain a homeostatic stability against states of excitation. These defences were thus necessary to prevent against states of high energy or high energy expenditure. For Freud, this separation of was based on his economic theory of mind. Conscious and unconscious was separated because of the damage which excessive energy can do since through and ideas could be invested with energy. This investment was called the *besetsum* or the cathexis as it is more widely known. *The unconscious has too much energy*.

In *Beyond the Pleasure Principle*, Freud (1920) described the protective shield against stimuli as being a two-sided surface entity which is exposed on both sides. Thus, it faces internal and external forces (Johnston, 2008; Freud, 1920). While organisms can remove itself from sources of excitation and pain in its *Umwelt* (external); it cannot escape from *Innenwelt* sources of the same. This is why it is the id-body of the drives which forms part of primal repression (Johnston, 2008). Thus, Freud has perfectly described too the nature, functions, and limits of the membrane (below).

1.2 The return of the repressed

Primordial repression is that which opens up the temporal dimension; but is itself *eternal or atemporal*. Hence, unconscious is not the opposite of consciousness—but the unconscious describes the very founding gesture of consciousness itself (Johnston, 2008). This the gesture of choosing oneself, and creating a synthetic unity of the self (Žižek, 1996; Johnston, 2008). The unconscious then includes the primal repression (as divorce) and the *trieb*, as is *atemporal* (or, out-of-joint). The divorce as an act, creates both consciousness and unconscious; with the divorce then being absorbed by the unconscious which it produced.

To relate this back to Freud’s economic mind; in *Inhibitions, Symptoms and Anxiety,* Freud speculated that primary repression must occur—due to early outbreaks of intense anxiety, which happen before the formation of the superego (Johnston, 2008; Freud, 1926). The prefrontal cortex (PFC) behaves as a superego, and it only fully matures in the mid to late twenties (Sapolsky, 2017). During this maturation, there is another process called neural Darwinism; wherein neurons must be

die for proper, efficient functioning. Thus, Freud concludes that primal repression must come about because of quantitative factors such as excessive degrees of excitation and crossing the threshold.

The truth or the retroactive construction is based in the present-as-future; hence, it the truth is contingent on us writing (and re-writing) the past. We give the past its meaning and its truth based on current understandings; retroactively. Thus, the Freudian act of repression and the return of the repressed coincide; the repressed is itself the retroactive effect of its return (Žižek, 2012).

Trauma presents a good way to present this temporal paradox. A traumatic memory is one of the past—however, it appears within the present of an individual's experience, thus blockading the individual's future. Thus, trauma is a "point of view" returning from the "future" with the aim of reconciling its own understanding and meaning. From the perspective of the "present", the trauma event is in the past. This trauma is also within the present, as it is experienced within the present. When the present tries to deal with this trauma—the present must position itself as the "past (of the trauma event)—which renders the present, "the future" within this reconciliation. This reconciliation involves creating a narrative of events of the past (to create the trauma event), from the present-as-future. Hence, this is a retroactive narration, and creation of a past event from the perspective of the future (as present).

1.3 **Neural transmissions**

At the individual neuronal connection level, the structure of learning and prediction is possible due to the action potentials (axonal spikes) in neurons which is an electrical signal. Neurons are connected to one another through axons and dendrites; the point of connection between an axon and dendrite is called a synapse. Thoughts and perceptions are determined by these spikes (Hawkins and Dawkins, 2021). The synapses are responsible for memory. *Memory is a product of sequential pattern activation in synapses of many neurons* (Hawkins and Dawkins, 2021).

Prediction includes one's own movement in every part of the body and predictions of one's own movement in the world. In terms of sequential activation, this is possible through *dendrite synapse* spikes. After receiving input, the distal synapse spike occurs when other dendrite spikes occur close-by and travel to the cell body priming the cell for activation (a predictive state) by raising its voltage (Hawkins and Dawkins, 2021). This is known as *neuronal oscillation*. The mini cortical columns' response to the same input patterns and *unexpected inputs cause more firing than expected ones* (Hawkins and Dawkins, 2021). When an input is predicted, then only the predictive state neurons fire. Therefore, prediction works by pattern formation and activation in the brain through both kinds of synapses. It is the neurons within the brain that recognise patterns of activation, thus determining when it too should activate through dendrite spikes. This is the common cortical algorithm (Hawkins and Dawkins, 2021; Hawkins and Ahmad, 2016). *Prediction happens inside neurons themselves* (Hawkins and Dawkins, 2021). The predictive state neurons *will fire*, and the others will be inhibited.

Firing of neurons means the transmission of nerve impulses through sending out electrical impulses. Activation of neurons happens as per the predictive model above. On a more technical ground, the predictive modality is based on stimulation from light, chemical information, or heat from surrounding cells or membranes. Different types of neurons require different kinds of stimulus to fire. The fluids inside neurons are separated from the outside by a polarized membrane which contains electrically charged ions.

The brain processes signals and time in the following way. At the more granular level, it is at the neuronal level there is a temporal element in the electrical signals between individual neurons. At this

level, neurons fire with the use of sodium ions and calcium. Dendritic action potentials for example occur when voltages are spiked (priming) using positive ions (when calcium is used it is called dCaAPs) (McRae, 2023). Neuronal signals operate in a wave-pulse form wherein the openings and closings of channels exchange these charged ions (sodium, chloride, potassium and now calcium too). The pulse is the flow of these ions, an action potential. These pulses are communicated in three ways: (A) AND; (B) OR; and (C) EXCLUSIVE OR. (A) stipulates that if neuron (X) AND neuron (Y) are triggered then the message is passed one. (B) stipulates that if (X) OR (Y) is triggered, the message is passed on. (C) permits a signal ONLY IF another signal is graded in a specific way. (C) is the relatively new (dCaAPs) signal pathway discovered (McRae, 2023).

When neurons reach the neural threshold (anything above causes firing, anything below does not), depolarization results (ScienceDirect, n.d.). This is a change in the cell's potential. "Potential" refers to *differences in electrical charges*. There are two types of potentials: (1) the rest potential; and (2) the action potential (as above). The neural threshold must be reached before a neuron can go from a rest to an action potential. The firing, which occurs after the threshold has been reached, means that the membrane's permeability is changed. By polarizing the membrane, a change of electrical charges (going from negative to positive) is enabled, which runs along the entirety of the cell membrane. After this, the neuron returns to its resting potential. This means that neural transmission is *determined by the threshold and whether the stimulation meets that threshold*.

Whether the threshold potential is reached is determined by the amount of charge which is transferred across the membrane (ScienceDirect, n.d.). The threshold is determined by the neuron and not the stimulus. The parameters for the stimulus within the threshold relate to strength and duration of the stimulus. *If the threshold is reached, the amplitude of the resultant action potential is the same, regardless of the level of stimulation*. This relationship between levels of stimulation and the production of impulses is called the *all or none principle* (Platkiewicz and Brette, 2010).

The action potential depends on the cell membrane permeability (a characteristic of the cell and not of the strength of the stimulus which triggers it) (ScienceDirect, n.d.). *The greater the duration of the applied pulse, the smaller the current intensity required to excite the fiber* (ScienceDirect, n.d.). However, a current can be applied for an infinite amount of time, but if it is below the threshold value, it will not cause the firing. There is also a minimal stimulation time required—which is the shortest duration of stimulation which is capable of producing excitation (even if massive currents are applied) (ScienceDirect, n.d.).

Membrane impedance is high when in the presence of high frequencies (alternating current (AC), for example). The higher the frequency, the less membrane impedance (resistance to transferal of energy per unit charge) results—and thus, the potential difference which can be produced across the cell membrane is low (ScienceDirect, n.d.). *What this means is that alternating currents of high frequencies has a smaller tendency to electrocute, and the energy of these currents can be dissipated as body heat* (ScienceDirect, n.d.). The higher the frequency of electromagnetic waves means that the wavelengths are smaller. Conversely, higher frequencies also means that it carries more energy. This is important; in plainer terms, we can say that the higher the frequency of the AC, combined with a very short duration in the alternations, is safer for the cells to transmit charge and (radiate) dissipate heat.

The question is then: On what is this threshold value based, and does it vary? One argument is that the value is determined in a non-linear fashion, namely it can be a combination of many variables including membrane potential and complex features of inputs (Platkiewicz and Brette, 2010). *This*

means that it can depend on preceding rates of depolarization and preceding inter-spike intervals (Platkiewicz and Brette, 2010). Studies have also indicated that the threshold can adapt to slow changes in the input characteristics (Platkiewicz and Brette, 2010). The variation too depends on the neuronal cell types and distances to the soma (Platkiewicz and Brette, 2010). In terms of spike threshold variability, it was found that a threshold formula provided an instantaneous time-varying value which agreed with the traditional statistical models (Platkiewicz and Brette, 2010). The sodium voltage gated channels which are constitutively responsible for cell excitation *mediate a positive feedback mechanism which produces the constitutively necessary instability required to initiate actions potentials*. Importantly, it was also determined that the *threshold for the Na activation gate depended on Na inactivation and conductance's* (Platkiewicz and Brette, 2010). This can explain the *effects of preceding spikes and membrane potential histories on cell excitability* (Platkiewicz and Brette, 2010). In other words, the threshold variability value is dependent on Na inactivation value. Thresholds are also inversely correlated with previous inter-spike intervals, namely if the inactivation time constant is long in comparison to the inter-spike interval, it is likely that the threshold would be linearly correlated with the firing rate (Platkiewicz and Brette, 2010).

Once the neuron has fired, it enters into a bi-part-phase called the *absolute or relative refractive period* (Bielajew et al., 1982). For a short period of time after firing, the absolute refractive period means that the neuron will not fire even if the stimulation threshold is reached or if there is massive stimulation. The relative refractive period allows for firing but only if the stimulus received is stronger than the threshold. After this period passes, the neuron will enter the rest potential and be able to fire "normally" once more. The more failure (in the instance of predictive error) the more neurons will fire and thus a heightened stage of consciousness arises (Hawkins and Dawkins, 2021).

On a granular level then, this is a retroactive constitutive negativity. The retroactive constitution is based on presently non-existing events or things (which may have existed in a particular state in a past time) which then shapes the "present". The negativity speaks to a negation—namely setting of a threshold. History moves forward!

1.4 The unconscious

On this, Naidoo (2023c) says:

> "When Freud said "Wo es war soll ich warden" ("where it was, shall I be") he was referring to the alienation of a specific aspect of oneself, namely the unconscious (Strachey, 1964). Freud wanted the unconscious (as substance) to be understood as subject too (Žižek, 2012). Thus, one of the goals of psychoanalysis is to enable the subject to realize that it is his own repressions that gives the appearance of content formations within the unconscious.
>
> Freud also drew a distinction between repressed content and the form of repression itself (Žižek, 2012). The key difference here is that the form of repression continues even after any repressed content is no longer being repressed. If the subject can free the repressed content, the repression itself stays behind in its form. As with Freud's conception of dreams, it is the form itself which propagates a feeling of a hidden content kernel of truth. The movement from Kant to Hegel explicates this perfectly. Briefly, Hegel's argument was that predictive reason, or apperception, creates the idea of essence as being behind appearance. The distinction between form and content is the same (Žižek, 2012). The distinction between form and content is a distinction within content itself (as the distinction between essence and appearance is within appearance itself).
>
> The Freudian unconscious is thus the primordial repression. However, it goes further than this. The primordial repression (Ur- Verdrängung) persists because the repressed content does not pre-exist the

> repression; it is rather retroactively constituted by the process of repression. It is like the notion that the law itself, through its criminalization of certain acts or ethics, can make the criminalized content desirable through its very repression. Primordial repression is not a repression of some content into the unconscious; it is rather the repression itself which constitutes the unconscious (creates it)".

The goal of psychoanalysis is to bring the subject to the understanding that *it is their own repression* which constitutes the unconscious. It is a repression of *nothing.*

1.5 Repetition, memory, and transcendence

As Sapolsky (2017) has pointed out, the prefrontal cortex is an honorary member of the limbic system whilst also being part of the cortical system. It also acts like a "superego" in decision making and rule development. Furthermore, the regions of the PFC are responsible for creating state spaces through stochastic reasoning (the subjective "how would I feels if x happened"). Evolution too functions according to creating redundancy in preparation for failure (Nowak et al., 1997). Additionally, memory also functions according to repetition. In 1954, Brenda Milner discovered that the medial temporal lobe and the hippocampus mediate declarative (explicit) memory storage, which is a conscious memory for people, objects, and places (Kandel, 1999). In a follow up study, she discovered that the same subject of her previous study was not able to consciously recall new memories about people, places of objects; but that he was still capable of learning new perceptual and motor skills (Kandel, 1999). This is known as procedural or implicit memory, and these are completely unconscious and are only observable in performance, not conscious recall. *Constant repetition which can transform declarative memory into procedural memory* (Kandel, 1999). Learning new rules requires conscious activation of the PFC to learn and abstract new rules. However, after time, these rules are stored and become a nonconscious "knowing" or activity. Learning to drive is an example—one is still thinking when one drives; just not consciously. Procedural memory is a collection of processes involving different regions and systems in the brain including recognition of stimuli, sensory regions, cued emotional states of the amygdala and the basolateral amygdala and others including processes in the cerebellum and the explicit system of the hippocampus (Kandel, 1999).
