# Supplementary material for: Open optimism as an “embodied-health” ethic for the information era
Source: Front Pharmacol. 2024 Jun 17;15:1331237. doi: 10.3389/fphar.2024.1331237 (PMC11215117; doi:10.3389/fphar.2024.1331237)
Supplement: Supplementary file 5 [file DataSheet6.pdf]

frontiers

Supplementary Appendix

Open-optimism as an "embodied-health" ethic for the information era

1 Utter extinction

Generally, evolution is thought of as evolution of life or biological form. However, evolution also applies to *all systems* and categories of knowledge including physics, cosmology, mathematics, language, and phenomenological experience. At its very basis, evolution is a law which maps the initial states of things (at a time) to the state of something (at another time, which is usually an end state or somewhere between). Thus, evolution is about reconfiguration (Hossenfelder, 2022). However, one must understand that evolution is fundamentally not about the physical state of a system, *but of its non-physical informational state*. It is an evolution of information.

When Darwin was conceptualizing his theory of evolution, he also investigated the link between "origin" and taxonomy. On this, Darwin (1895) said:

> "... the manner in which all organic beings are grouped, shows that the greater number of species of each genus, and all the species of many genera, have left no descendants but have become utterly extinct".

Darwin believed that *utter extinction* (no descendants are left behind) is what creates taxonomic classifications (Winsor, 2023). Utter extinction is not about leaving fossils or traces; *it is about leaving no descendants* (Winsor, 2023). Thus, for Darwin, not only are there common ancestors from which origins can be traced through lineages; there are also extinct past species which are no one's ancestors. In fact, most past species, he believed, fall into this category (Winsor, 2023). The loss of these species with no descendants creates taxonomy.

From around 1832, Darwin intensely sought out and studied fossils and records (particularly the Megatherium). In doing so, he was analyzing the connection between extinct and living edentates (Winsor, 2023), when it occurred to him that there was a connection between the past and the present. The primary dimension of taxonomic relations was linked to the second dimension of time (Winsor, 2023). The third dimension of space, for Darwin, spoke to distribution and relations within geologic locations. At the time, one of the common understandings of extinction was that it fit *within* taxonomic systems; not that it fit between existing groups (creating them) (Winsor, 2023). Lamarck, for example, did not believe that extinction could fit into an orderly world, hence fossils must show that species changed linearly (Winsor, 2023). Interestingly, *Darwin rejected the notion that species may have a natural life span the same way that individuals do* (Winsor, 2023). He reasoned that armadillos and sloths live in the same region where he found the remains of the giants they resemble. In terms of his tree of life diagram (or the "I think" diagram which he called because he did not want to obfuscate with metaphorical language), this would be jumping from one branch to another. However, the issue that he still had was why there were gaps in the series of connection (Winsor, 2023). His "I think" diagram he reasoned should be called the coral of life diagram *since the base dead branches could not be seen or represented* (Winsor, 2023).

Evolution is thus the cause; and taxonomic groups is the consequence. In postulating about species generation, Darwin noted that it was the *size of the gaps between species which cause genera* (plural of genus) (Winsor, 2023). There would be two genera, for example, because of a massive gap between both; it is the gap which constitutes the genera classifications/categorizations (Winsor, 2023). *Genera or genus then is somewhat subjective, but also not arbitrary because genus expresses or describes real past events* (Winsor, 2023). Darwin's "I think" diagram, in other words, works on two principles: (1) evolution proceeds by branching; and (2) genera are formed by descent by a common ancestor.

On utter extinction, Darwin said that the tendency to change and to multiply when isolated requires the deaths of species to keep numbers of forms equable. In explaining the importance of keeping the number of forms equable, Darwin says that this is based on the subdivision and amount of differences so forms would be about equally numerous (Winsor, 2023). The word "form" refers to species; it was species as the operative unit which was responsible for the branching (Winsor, 2023). In other words, in the context of genera, species must stay constant, and the addition of new species has to be balanced by the subtraction of others through utter extinction. Darwin believed that while actors on stage may change, the theatre itself does not change, hence why he believed that the number of species must remain constant (Winsor, 2023). Utter extinction thus allows for this regulation and is the constitutive gap which delineates the categories. This gap, Darwin postulated, must be the transitional organism which creates the transitory gap between categories. Groups are made by reproduction and multiplication, and extinction forms the gaps which constitute the groups (Winsor, 2023). Genera are thus formed (Winsor, 2023).

When discussing divergence in *Origin of Species* (how species became more and more different from each other as they evolve), Darwin (1895) realised that he needed to account for the tendency of organic beings descended from the same stock to diverge in character as they became modified (Winsor, 2023). Thus, he postulated that the genera are formed bearing relation/resemblance to ancient types (which, like the usage of "form", was indeterminate and not referring to any specific taxonomic group) (Winsor, 2023). In other words, everything has an ancestor, but not everything has a descendant (Winsor, 2023). Going back to the gaps, Darwin realised that these massive gaps over periods of time are to be expected, since there must be a movement towards stability of species through extinction. So, where there are gaps in categories, these gaps are produced by the utter extinction of the otherwise intermediary or transitory phase/form. This utter extinction is related to population dynamics. Nature and its groupings thus must be carved out by utter extinction, with the ancestors and their relatives being extinct too (Winsor, 2023). Darwin also postulated that groups of higher rank are separated by bigger gaps than those of small ranks (the gaps between genus are bigger than the gaps between species) (Winsor, 2023). The amounts of differences between species is then caused by the loss of ancient relatives (Winsor, 2023). The ranks of hierarchical organization reflected the different times of living between the ancestor groups and the lower ranked groups. At this point Darwin supposed that natural groups may have a repeating pattern—multiplication and utter extinction.

Utter extinction then means that most species which existed in the past are not an ancestor to anything. This is similar to dark matter which is invisible since it omits no light (Winsor, 2023). The existence of dark matter is only an indirect inference. Utterly extinct species, which are invisible but real, outnumber living ones massively, in the same way that dark matter outnumbers regular matter. Utter extinct life cannot be seen since life often vanishes without a trace, and that fossil records with resemble living species cannot tell us whether it is a direct ancestor (Winsor, 2023). *The Principle of Divergence, which Darwin wrote about in Origin of Species, details how differential extinction*

creates diversity (Winsor, 2023). The more extreme forms of a species is more likely to survive since they may have novel ways of dealing with affordances within their niches. In this way, nature does not make leaps, *it evolves over long periods of time. However, what we observe are leaps*.
