# Supplementary material for: Open optimism as an “embodied-health” ethic for the information era
Source: Front Pharmacol. 2024 Jun 17;15:1331237. doi: 10.3389/fphar.2024.1331237 (PMC11215117; doi:10.3389/fphar.2024.1331237)
Supplement: Supplementary file 6 [file DataSheet14.pdf]

Supplementary Appendix

Open-optimism as an "embodied-health" ethic for the information era

1 Temporality and generative entrenchment

Temporal constraints are the kinds of constraints which can promote flexibility, through *differential entrenchment*. Temporal constraints for example, may only be operative upstream, and not downstream (Juarrero, 2023). Differential entrenchment regimes are known as *generative entrenchment* (Wimsatt 2001; 2007). Different kinds of entrenchment precipitate different forms, and degrees of stability. *Extreme entrenchment is incompatible with the capacity to evolve*, which in turn impacts a systems *resiliency* and *antifragility* (Taleb, 2004). We observe post traumatic growth every time a bone breaks. It heals to be stronger than it was the last time. The same applies to muscles; for muscles to be built bigger (for bodybuilding), fibers must be first be torn apart and then rebuilt (during rest). We also see it in dealing with traumatic loss, wherein people experience growth and become "stronger" (Collier, 2016). Constraints which promote resiliency and antifragility, whilst simultaneously securing a *minimum value of stability*, is also known as the process of *generative entrenchment* (Barber 2016; Wimsatt 2001).

Generative entrenchment provides "safe-to-fail" insurance policies for complex systems, by creating resilience and antifragiltiy (which is the capacity to manage fluctuations and perturbations) through *self-modification* or *self-reference* (Juarrero, 1991). These are systems which incorporate failures into their repertoires, widening the scope of their repertoires by using failure as an affordance. Redundancy/robustness on the other hand, describes a system/mechanism, which is *fail-safe*, by *trying to prevent failures from occurring*. Successful generative entrenchment thus requires constraints which provide a minimal stability value of fundamental constraints, whilst also ensuring long-term flexibility.

Generative constraints can do this through differentially expressed constraints. This includes *temporal constraints* which can be *differentially, sequentially, and conditionally* activated (upon *timing* and *contextual conditions*). Generative constraints can impose rigidity upstream temporally, but more flexibility downstream, thus allowing for novel interdependencies to be formed (Juarrero, 2023). Generative entrenchment can also narrow, or firmly taper off possibilities upstream temporally, in a diachronically constrained dynamic, whilst simultaneously facilitating unanticipated interactions and other enabling constraints to appear and function downstream (Juarrero, 2023). In the long term, regimes of differentially expressed constraints that enact generative entrenchment—are *self-ratcheting*—and promote resilience/antifragility. Generative entrenchment can also stabilize novel, emergent coherences (Juarrero, 2023). They can do this, through their memories, by recalling the importance or value or reason for the entrenchments (Wimsatt, 2007). However, generative entrenchments will only perform this for those constraints which promote stability, resilience, antifragility, and those which sustain evolutionary capacities. (Wimsatt, 2007). Hence, *the capacity for evolution depends on generative entrenchment—and generative entrenchment is likewise dependent on the capacity for evolution*.

When each stage is stabilized, systems can then explore *local, current possibility spaces without the risk* of becoming unstable or incoherent. (Juarrero, 2023). Local activation of downstream constraints

enables new coherences and niches, with emergent properties and powers to appear, as per the *local context*. Hence, differential spatiotemporal constraint activation, or inhibition of governing and generative constraints promotes stability, coherence, antifragility, and resilience. Generative constraints thus account for abduction or Gould's "exaptation" which are the functional properties that emerge as novel coherent dynamics of generation through contextual constraints, which function in different contexts, and at different times (Juarrero, 2023).
