# Supplementary material for: Open optimism as an “embodied-health” ethic for the information era
Source: Front Pharmacol. 2024 Jun 17;15:1331237. doi: 10.3389/fphar.2024.1331237 (PMC11215117; doi:10.3389/fphar.2024.1331237)
Supplement: Supplementary file 7 [file DataSheet9.pdf]

Supplementary Appendix

Open-optimism as an "embodied-health" ethic for the information era

1 Sedimentation and entrenchment

Constraints both facilitate the emergence and persistence of coherence, whilst also sedimenting or entrenching said coherences—thus, enhancing *metastability*, and preventing coherences from reaching thermal equilibrium (Juarrero, 2023). Sedimentation and entrenchment (valleys, ridges, and walls) preserve existing equilibria from potential or actual perturbations. Sedimented or entrenched constraints are difficult, and costly energy wise, to relax, remove or modify, in comparison with the expenditure required to remain/persist *(efficient*). Hence, it requires more energy expenditure to overcome the hillock of the attractor in which a system is embedded, and into a neighboring attractor (Juarrero, 2023). Sedimented and entrenched constraints render attractors in *a possibility space* to be deeper embedded, and thus more persuasive or pervasive, thus enforcing governing constraints. This also serves to reinforce habits and *reduces the degrees of freedom of actualizing diversity of behaviour or phenotypic traits* (Egbert and Barandiaran 2014) of a possibility space, to a smaller set of empirical possibilities (Juarrero, 2023).

Sedimentation and entrenchment are important context-independent constraints that reinforce default governing constraints (initial conditions), preserving them from reaching thermal equilibrium. This does have a negative impact, namely, the system becomes less flexible, and has a lower response possibility space for contextual conditions (Juarrero, 2023). When systems are under high levels of stress, threat, or approaching thresholds, systems need to be able to *adapt quickly*, and *ascertain new gradients of energy or information*, or be able to quickly unlock *enabling constraints*, which can aid survival.
