# Supplementary material for: Open optimism as an “embodied-health” ethic for the information era
Source: Front Pharmacol. 2024 Jun 17;15:1331237. doi: 10.3389/fphar.2024.1331237 (PMC11215117; doi:10.3389/fphar.2024.1331237)
Supplement: Supplementary file 8 [file DataSheet11.pdf]

Supplementary Appendix

Open-optimism as an “embodied-health” ethic for the information era

1 (Self)entrenchment and flexibility

Entrenched constraints have a higher resistance than sedimented constraints. When existing governing constraints *self-reinforce* through mechanisms such as co-optation, the result is that sedimented constraints hold-fast interactional types, thus enhancing the spatiotemporal *metastability* of said interactional types (Juarrero, 2023). Entrenched constraints can reinforce metastability, by *smoothing out* short-term fluctuations or perturbations. In this way, entrenched constraints prevent short-term malfunction thus enhancing persistence. Entrenchment constraints apply to processes *and phenomena*, such as *top-down intentions* to produce an entrenching constraint or artifact, or the entrenched artifact itself (Juarrero, 2023). A legal constitution for example, is entrenched within law, (as a superseding source of law), whilst simultaneously containing its own provisions for amendment, which are purposefully difficult. If entrenched constraints are *inflexible*, they can *freeze conditions entirely* (Juarrero, 2023). If this happens, the entrenched constraints have the same function that context-independent constraints do; by “hardening” constants, co-ordinates and attractors of a possibility landscape wherein local context-dependent constraints operate (Juarrero, 2023).

Self-imposed entrenchments (like wills) can *freeze* existing conditions, by disallowing completely any future modifications. This happens when self-embracing entrenchment constraints use meta, or second-order constraints/inferences (Naidoo, 2023a) which control how and the extent that first-order constraints/inferences are able to be modified. This requires processes of recursively defining systemic rules, including the specifying procedures or instances whereby specific rules can be altered (Juarrero, 2023). Thus, systems which contain entrenchment constraints or rules which are revocable, or modifiable, are protected against complete inflexibility, through self-construction or self-embracement of entrenching mechanisms. This enables evolvability, whilst also protecting the system’s stability from perturbations. The capacity for evolvability must promote an active resilience and antifragility, both of which enhance viability and persistence over time. Hence evolvability requires that initial constraints can adapt to *changing contexts* (Juarrero, 2023).

Entrenchment can occur naturally and deliberately. Deliberate entrenchment includes the already mentioned legal constitutions, which are social systems designed to strengthen, heighten, facilitate, or stabilize social organizations. They also aim to decrease decoherences, or disintegrations of those social organizational types. Often entrenchment can be used to deliberately preserve social coherences or organizations which entrench certain dominances—thus can be nefarious.

Typically, deliberate entrenchments would begin with the mechanism of “dampening” which serves to limit fluctuations and prevents runaway accelerations. Dampening functions as a “shock-absorber” (Juarrero, 2023). Dampening thus prevents systems from getting too close to thresholds of instability, by resisting change and risk. However, the stability enforcing mechanism of dampening does contain its own risks—namely that systems which rely on dampening, can only persist if they continue to dampen, which renders them unopen and resistant to change—thus, making them inflexible and rigid. Hence, these systems cannot adapt to contexts which are in flux, and these systems cannot evaluate

long-term interests. By restricting token realizations to fewer amounts or singular realizations, any interdependencies (and systems) are made *fragile* (Juarrero, 2023). Systems must adapt and evolve if they are to persist long term. Systems which display an extreme-context dependence are also brittle—as the system is now overly reliant on contextual conditions for its fitness and survival. Thus, what is necessary, are regimes of *governing and enabling constraints*.
