# Supplementary material for: Open optimism as an “embodied-health” ethic for the information era
Source: Front Pharmacol. 2024 Jun 17;15:1331237. doi: 10.3389/fphar.2024.1331237 (PMC11215117; doi:10.3389/fphar.2024.1331237)
Supplement: Supplementary file 9 [file DataSheet3.pdf]

Supplementary Appendix

Open-optimism as an "embodied-health" ethic for the information era

1 Sex and novelty

There are important differences between replication, and reproduction (sex). Replication is mechanism of "more-making". More-making involves making copies/duplicating already formed, coherent, existing, *wholes* (as opposed to just parts of a system) and coherent entity. Biological asexual reproduction, as mitosis, is an instance of replication, wherein two identical daughter cells are produced, which are copies of an already existing and coherent whole. These are new systems, but they are copies/replicas of the parental material (Juarrero, 2023). The process of mitosis thus cannot produce type diversity or variety.

Meiosis (sexual reproduction) involves the process of producing gametes, in the form of four haploid cells, each of which would contain only half of the genetic material of the parent. Two haploid cells must combine to form a full set of genetic material. Hence the haploid germ cell is not a duplicate of the parental genetic material, which means that the germ cell does not duplicate the parents' constraints or their coherent organization. Thus, the need for combination, introduces contextual information into the process, which thus impacts the genetic material of the offspring. It introduces contextual factors, and *choice of partner*.

To summarize, gene shuffling which as the enabling context-dependent constraint of sexual reproduction, does not make copies/replicas as mitosis does. Gene shuffling creates novel combinations, in the form of novel token interdependencies (Juarrero, 2023). In doing this, gene shuffling *endogenously* introduces variation (coordinated patterns having larger possibility spaces).

By combining gene shuffling, with horizontal gene transfer, the result is that both constraints actualize the formation of multiply realizable diachronic interactional types, better known as lineages. Lineages conserve the unity of type, whilst simultaneously enabling for *increasingly diverse tokens* (Juarrero, 2023). These tokens are novel interactional types, which can have enhanced viabilities. Lineages too, persist longer individual token realizations. Lineage types thus serve as a limit to the possibility/adaptive spaces of organisms, whilst sexual reproduction introduces both flexibility and viability. Hence, lineages and sexual reproduction work in tandem.
