# Supplementary material for: Open optimism as an “embodied-health” ethic for the information era
Source: Front Pharmacol. 2024 Jun 17;15:1331237. doi: 10.3389/fphar.2024.1331237 (PMC11215117; doi:10.3389/fphar.2024.1331237)
Supplement: Supplementary file 10 [file DataSheet1.pdf]

Supplementary Appendix

Open-optimism as an “embodied-health” ethic for the information era

i. Preliminary note to readers

This is a difficult manuscript for the following reasons: (1) it requires multi-modal reasoning, being multi-thread reasoning (think, keeping multiple tabs open on a computer, with each table keeping track of an argument/concept/idea that is building, and thus being increasingly populated); (2) the equivalency, reliance and conceptual footwork done in several fields, including theoretical physics, informational sciences, biological sciences, German Idealism and others; (3) the many novel interconnected secrets, which are not always explicitly stated, but rather implicit to the built argument and (4) the thematic headings presented in this manuscript function in a synchronistic unity, and not through a linear direct causal flow. As a result of these points of difficulty, each heading/concept/argument/idea (or thread), is interlinked with others, and thus, the manuscript would typically require more than one reading to be fully digested and understood. I have done purposefully, because this manuscript has a hidden aim, which is reflected in the conclusion. This secret aim is the authors intention, which is to provoke the reader into a state of self-reflection, on their own selves. That is what the concept of openness really means – self-reflection, not reflection on an object (being this manuscript itself). In this way, I have tried to put into practice the strongest from of subjective freedom as I can, in this medium, by ensuring that the reader can actively participate in their understanding, rather than being treated as determined blank slate, upon which the ideas of this manuscript are meant to imprint. However, to help aid this process, I have provided a navigational map below, which outlines some of the interconnectivity between the sections. There are different forms of interconnectivity, including synchronization, or demonstrable equivalencies. To end, in my home country of South Africa, there is a cultural phrase which embodies this idea- “dala what you must, ekse.” This phrase means, “what you put in, is what you get out.”

ii. Introduction (1)

The introduction begins with posing the two key thematic issues, being that of linearity and efficiency. This heading relates the notion of efficiency, to backward propagation, which is an important technique in AI, and an important point of conversation, given that that Hinton, the “inventor” of the technique, cited AI as being a human-level threat. The movement of efficiency, because of backward propagation and regression techniques, involves the reduction of risk (risk of failure) and energy expenditure. A reduction of such creates linearity, through the reduction of degrees of meta-freedom (2, 11, 12, 16; 20-23), thus denigrating optimism, or embodied health (17).

iii. Stochastics and human biology (2)

Stochastic reasoning is a method/model for dealing with decision making under uncertainty, unpredictability, or incomplete information (5, 6, 9-16). Stochastic reasoning typically involves the simulation of various alternate subjective paths/trajectories (12-17). These paths map qualitative and quantitative information, such as which outcomes are possible, which evental sequences lead to certain outcomes (3-8 in particular), and other relevant information. Stochastic reasoning enables systems to be antifragile, instead of robust. Stochastic reasoning enables (as the a priori) for the possibility of qualitative inferences (13-16 and 23). Qualitative inference as described in the conclusion (23), is philosophy, which is required for the construction, maintenance, and protection of embodied health.

Stochastic modelling is thus an instance of history moving backwards, rather than forwards (this links with regression/backward propagation techniques in (1; 3-5; 9-10 and 20-21). Stochastic modelling is thus the a priori for efficiency (1) and is an instance of both the imagination (18) and meta-freedom (1, 11, 12, 16; 20-23). Stochastic modelling also links to the dopamine system as described in (17). Once more, like the dopamine system, the goal itself (the object/point) are the subjective paths, not an object, or endpoint in itself. In other words, stochastic modelling is about the journey, not the end-point/goal.

Importantly, stochastic modelling in the human brain involves the suppression of non-existing, non-ideal possibilities, from eventuating (this links to the Freudian idea of the repression of nothing described in the manuscript and in 22). This is the Kantian infinite judgement, or the intentional misrecognition, which is the subjective choice not to constitute events as such, thus rendering a system as being open (18-23). That is another demonstrated equivalency.

iv. Frame axioms: Epistemology and computation (3) and Planning, prediction, and explanation (4)

Headings (3 and 4) provide important information for non-technical audiences, like the lawyers and the ethicists. The importance of both headings (3 and 4) lies in the description of issues inherent to constructing autonomous agents, and the issues in relation to providing/enabling degrees of freedom, for said agents (degrees of freedom is an important concept throughout the manuscript – see 7, 8, 11, 13-17, 19 and 23). Importantly, this section also provides detail and explanations as to the workings of backward propagation and regressive functions. These explanations are important, given than there will be an equivalency demonstrated, between backward propagation and regressive functions, with the Hegelian notion/logic of the incomplete Absolute, which involves a retroactive construction in (9 and 10). These ideas will then be synchronized with the physics of the system of reality (6-8; 13-16).

v. Explanation closure (5)

Explanation closure axioms are the solution to prediction problems involving artificial agents, and human agents. Given that reasoning is complex, and real-world problems are complex too, one cannot account for all possible variables in the form of facts, and thus programme in factual based axioms (explicit frame axioms). Explicit frame axioms are impossible, or highly improbable to be accurate – thus epistemic assumptions (11) are necessary (the incomplete Absolute in heading 9 and 10 below)

The solution is thus to use explanation closure axioms, which code for transformations upon the occurrence of certain events. These assumptions are then event orientated. An event-centric epistemology and ontology is thus required, as provided for in (6-23). Event centric paradigms are also non-linear (17).

Moreos, explanation closure axioms and an event centric paradigm provides the framework for contextualism, contextual import, adaptation/evolution and treating information on a case-by-case basis (12). An approach like this, also is separate from the computational formulism, which is fundamental to enhancing subjectivity and subjective agency, rather than enforcing a strict determinism on “agents.” Subjective agency is what renders agents, as agents.

Most importantly, explanation closure axioms thus code for their own incompletion; explanation closure axioms are completely-incomplete, by design. It is this incompletion, which is the a priori for the possibility of agency. These explanation closure axioms are equivalent to the incomplete Absolute below.

Explanation closure axioms also enable for self-referential logic (10), and the creation of non-linearities, thus ensuring contextual import, adaptation, antifragility and optimism, as embodied health (2, 12, 17 and 23). Moreso, the idea of a complete-incompleteness, links to and the notion that nature is indeterminate (11, 13 and 16), and the Kantian idea of a subjective power of will to overcome, in the

presence of failure (when being overwhelmed by acts of nature-18). The power that Kant is describing, is an instance of true subjective freedom, given that natural events of that kind, would only be constituted by subjective choice, thus enabling a subject with the power of determination (a double meaning, given that the subject can determine an event to be, or not to be, but a subject is also filled with the power to overcome, being motivation, or optimism).

vi. Hegel's theology: Events and virtuality (6)

This heading builds off (2-5) and provides the logical undergirding for the events ontology which proceeds from this heading (as mentioned for Hegel, God takes the form of events). This heading also provides the important concept of embodied logic, wherein Hegel merged the materialist and transcendentalist positions (19). In other words, the transcendent is intrinsic to the constitution of the material, and not extrinsic to it. This links to (12), given the power of subjective choice/interpretation when systems face perturbations in evolution (they can decide whether they can overcome, thus transcend), and (19) wherein Kant describes the power/motivation he feels when facing overwhelming natural forces (the power to overcome or transcend).

It is important to note that the Hegelian logic is subjectively driven, meaning that there is no end-goal for the movement of history (1, 2, 11, 20 and 21). Hegel's argument is thus that nature is not deterministic or determinate. Historical events are thus open-and open to re-interpretation/re-constitution.

This heading highlights the fundamentality of subjective registration/interpretation/belief in the actualization of possibilities/events, hence rendering events as contingent on subjective registration as per (20 and 21). This is an application of a dialectical logic, being that of co-constitution (7 and 8). The subjective interpretation mentioned prior, involves degrees of freedom, given that subjects can freely determine the constitution of events (7,8 and 16), or whether to not constitute an event. The latter, being the choice to not constitute an event, is based on the logic of the Kantian infinite judgement (13), or the intentional misrecognition (19 and 23).

vii. Dialectical discrete event centric physics: Events and observers (7) & Maps and events (8)

These headings provide an event centric approach to the physics of the system of reality, which forms part of a dialectic corroboration and dialectic constitution (mutual corroboration and mutual constitution) with: (a) the event centric model posed by computer sciences and programming logic in headings (3-5) and (b) and the event centric model posed by Hegel in heading (6). In terms of the dialectic, events are only constituted as events, upon subjective registration (thus without subjective registration, an "event" is not constituted as an event). The aforementioned links with (6, 11, 13, 15 and 16).

Moreso, the importance of the content of these headings includes a justification for dialectics in the form of reciprocal constitution, as the a priori for the constitution of events, which accords to the interpretation of Hegel provided in (6). This mutual dialectic thus highlights the need for emotions and experience, in any form of knowledge construction, including the physics of the system of reality. Emotion and experience in this regard, accords to uncertainty and surprize, which is central to the QBism interpretation of quantum mechanics, and Bayesian probabilistic inferential reasoning in statistics and computer sciences.

The principle of non-locality describes novel emergent properties (12), that arise from causal relationships between different variables on a map, and potentially with the map itself (there always needs to be a dialectic, or two variables). These emergent properties are non-local (hence, discrete) degrees of freedom, or degrees of meta-freedom (11-16; 19-23).

viii. The Absolute (9)

The Absolute is the metaphysical grounding for all knowledge, including all fields/subjects/disciplines (such as mathematics, physics, biology, linguistics, law and so on). All knowledge bases require at least one metaphysical/epistemological assumption. Despite that fact, this central foundational axiom is overlooked.

This manuscript and the proceeding heading, deconstructs the typical understanding of the Absolute, and demonstrates that this typical construction leads to nihilism, and thus a denigration of embodied health. The typical conception of the Absolute is a threat to free subjectivity (free-will) thus leading to nihilism because the typical construction implies determinism (and linearity). The typical construction of the Absolute, is equivalent to explicit or fact-based axioms, as in (3 and 4), and the concept of certainty (12 and 17), both of which result in nihilism.

ix. The incomplete Absolute (10)

The solution to the issue presented in (9) is Hegel's construction of the Absolute. The Hegelian version of the absolute is (a) incomplete; (b) internally differentiated; (c) retroactively constructed and (d) always beyond itself. In terms of (a), the Absolute is completely-incomplete, which is known as the "incomplete Absolute." This incomplete Absolute is equivalent to an explanation closure axiom, as per (5).

An incomplete Absolute enables for (b), the internal differentiation, which are subjectivities (or context-dependent constraints). This internal differentiation, or subjectivities, are thus necessary for the constitution of the incomplete Absolute itself. In other words, there is a shared dialectic between the Absolute (the totality) and its parts (the subjectivities). An incomplete Absolute enables for contingent subjective registration or subjective actualizations (6-8), which are instances of contextual free-will, or subjective "on-the-fly" choices (5).

Lastly, in terms of (c), the incomplete Absolute is retroactively constructed, which is equivalent to backward propagation and regression functions (3)-(5). This logic is explained using Freud, and the logic of "the return of the repressed," thus enabling for a mutual corroboration and constitution between: (a) the Hegelian incomplete Absolute; (b) backward propagation and regression techniques and (c) Freudian logic. This incompleteness of the Absolute thus enables for and requires acts of subjective freedom/agency to be "filled." The dialectic between the Absolute and its subjective constituents, is known as embodied logic, given that subjective acts are necessary for the actualization of the Absolute itself. This idea also connects with (11-16) wherein nature is noted to be non-determinate.

x. Epistemic constraints and possibility (11)

Relating back to the physics of the system of reality - this heading dialectically constitutes and dialectically corroborates headings (5)-(10). Firstly, this heading involves the understanding that nature is non-deterministic (10, 13, 16, 18, 21, 23), because of an epistemic constraint which is imposed on observers (10 and 18). The epistemic constraint is equivalent to a complete-incompleteness, as embodied by explanation closure axioms (5) and the incomplete Absolute (10), given that the Absolute is always being beyond itself (unable to fully grasp itself). This also links with (18), the Kantian Sublime, which is defined as being in-definable (which is paradoxically, how essence emerges).

Moreso the importance of this epistemic constraint in this physics of reality model, is that it is the a priori for possibility itself, by securing an irreducible/ineliminable inferential and interpretive capacity for observers within the system of reality. The aforementioned capacities are equivalent to subjectivities or perspectives, or context-dependent constraints, as mentioned in (10). Inferential or

interpretative capacities, involve degrees of meta-freedom, which is qualitative freedom for theorization (this idea is continued in the conclusion– 23).

To construct an irreducible form of ambiguity, like the Kantian Sublime as above, being an epistemic constraint, there must be a form of meta-reasoning, which results in a null/no-result/no-solution. This is the logic of aufhebung, or the unity-in-difference (23), which would take the form of a consensus-of-dis-consensus (we agree to disagree). The aforementioned concepts, are instances of a meta-difference, wherein differenc itself is conceptualized differently, thus ensuring that there can be no agreement, or resolution, hence enabling for a system to remain open, and allow for contextual import (also known as a pure difference). A meta-difference forms a topological knot, that ties two positions together, in such a way, that both positions are united by differences, rather than similarities (or, conflict, as expressed by Kant in 19). A meta-difference thus ensures that a system remains incomplete (as per 5, 10, 16, 18, 20 and 23). This is a subjectively induced gap within the system of reality (23).

xi. Ambiguity, evolution, and novelty (12)

The importance of this heading is the notion that initial conditions, or context-independent constraints must be vague/ambiguous for complexity or order to arise (thus non-deterministic-linking to 5, 10, 11 and 16). It is this vagueness or ambiguity, which enables (as the a priori) for the possibility of feasibility regions consisting of multiply realizable subjective narratives (subjectivities), which can play out/simulate. These subjectivities are stochastic paths/trajectories (2) which are also known as ontogenetic differentiations.

Waddington's landscape describes the biological topographical epigenetic landscape equivalent of differentiation and subjectivity formation. Note, the dialectic between motion and matter landscapes is necessary for the constitution of both landscapes/environments (contexts) and subjectivities (phenotypes).

Importantly, the dialectic for the constitution of events (6-8) is important once more, for the development of novel phenotypes, which hinges on environmental perturbations, and a subjective response/interpretation to those perturbations, as such. In this way, the "self-development" of a phenotype too is self-driven, based on self-referential interpretation (5 and 10).

xii. Ontic state spaces and encoding (13)

This heading demonstrates that non-linearity and non-determinism is inherent to the constitution of the physics of the system of reality/nature. A system of this kind operates asynchronously, which means that this system enables, and requires subjectively driven synchronization. The aforementioned guarantees free-subjectivity as per (9)-(11). Free-subjectivity/choice is actualized through subjective registration/belief, thus rendering the synchronicity of the system, contingent on subjective registration/actualization. Where there is an intentional misrecognition, or Kantian infinite judgement (19), an event will not be constituted, which once more leads to the plan recognition problem in (3-5). This intentional misrecognition is an instance of true freedom/autonomy (19, 20 and 23). It is the aforementioned, which keeps a system open (20 and 21).

xiii. Inferences and variables (14)

To make is easier to understand for other audiences, the physics terminology is made equivalent to legal and ethical concepts. This heading highlights that non-local emergent properties drive inferences/inferential reasoning. Importantly, the non-local variables, involve subjectivities and subjective actualization to come into being, given that these variables are implicit, and not explicit. In short, the actualization of these non-local variables is contingent and require subjective construction. This links with headings (5)-(11).

xiv. Probability (15)

This heading notes that probabilities arise due to hidden information, hence rendering probability epistemic in origin. This links to headings (7)-(13). This is also once more a reference to the incomplete Absolute, and to philosophy itself, as a concept, even within the physics field, given that physics is a strand of philosophy (physics and the other sciences used to be called natural philosophy or the philosophy of nature. This is the reason why there is a philosophy of science, but there is not a science of philosophy).

Moreso, this section highlights the necessity of the dialectic once more to obtain a firm outcome, and how subjectivity (in the form of subjective paths, choice and contexts as in heading 2) are interweaved into probability and predictions. This completes the question of how agents can reason and predict, as required in the logic of explanation closure axioms.

xv. An ontology of reality (16)

This section highlights the necessity of the dialectic for the constitution of a definite state of reality. The joint state space for both observers in the dialectic, is known as a consensus. In the conclusion (23), this consensus takes the form of an agonism (11 and 19), or the unity-in-difference, wherein the consensus reached, is a consensus of dis-consensus (we agree to disagree). It is a consensus of dis-consensus which enables a system to be open and enables for subjective freedom (a brush with the Absolute). A consensus of dis-consensus is equivalent to the Kantian infinite judgement (18), or an intentional misrecognition (19), thus ensuring that events, will not be constituted as such. If events are not constituted, then the system will not close, and thus remain open (10, 20 and 21).

Moreso, this heading highlights the necessity of hidden information as an a priori for: (a) meta-freedom, (b) the ability for interpretation and (c) thus subjective freedom and purposiveness. This links back to the concept of explanation closure axioms in (5).

This heading ends with the conclusion that the system of reality, is determined-to-be-open. Herein is the co-incidence of opposites (also known as the Hegelian aufhebung), wherein two contradictory terms are held-together-whilst-simultaneously-being-held-apart, in a unity-in-difference (23-this is also the logic of chirality in biology). The co-incidence of opposites here is explained in (21), where it is that (x) which is expected to be determinantal to something else (y), turns out to be the very a priori (x) for the possibility of that which it was supposed to harm (y). The example given in (21) is that of freedom and fate; fate is that which is the necessary condition for the possibility of freedom (echoed in 23 - the conclusion).

xvi. Nihilism: linearity and optimism (17)

This heading provides empirical evidence that linearity leads to nihilism, being the lack of subjective purposiveness or optimism. Hence, the solution is to move from a linear logic to a non-linear logic. To support this idea, empirical evidence regarding dopamine is provided. Dopamine, being the "feel good" hormone, and a large contributor towards optimism and hopefulness, is not absolute (the incomplete Absolute once more in heading 10). The amount of dopamine release is relative to: (a) the anticipation of the reward, and not the reward itself; (b) the uncertainty involves- uncertain (unpredicted) trajectories result in more dopamine release compared to the release value upon the obtaining of predicted/expected objects/outcomes; (c) the surprise factor (Bayesian) and (d) contextual factors.

More dopamine is thus released in: (a) the pursuit of riskier unpredictable trajectories (higher chance of failure); (b) the presence of prediction failures and (c) the pursuance of an uncertain or unpredictable trajectory – rather than the pursuance of an outcome or object (2 and 19).

In lieu of the above, the linear movement of efficiency, or linear logics, which is also known as optimization, is contra optimism, given that efficient trajectories provide more certainty, less surprise, and thus less dopamine release. Linear logics, and the movement of efficiency, is typically exemplified by standard academic writing (linear causal flows, which are presented as being clear writing), or the notion of "get to the point." These uncertain subjective trajectories are stochastic paths, as described in (2), wherein it is the path itself that is of value, rather than the object (the point/end-point). Moreso, this subjective purposiveness, which is tied to optimism and dopamine release, is equivalent to the ideas of Kant, in (18), who discussed the purpose of the mind, was the subjective purposiveness (self-referential reasoning once more).

xvii. Kantian aesthetics: The Sublime (18) and Kant, Gödel, Bartleby, Hegel, and Žižek (19)

The Sublime and the Kantian Infinite Judgement are two fundamental concepts. The Sublime is a necessity for the mind, according to Kant and it provides the mind with a subjective purposiveness, through agonism/conflict.

The Sublime in the mind is experienced as a transcendence through the conflict/agonism (or unity-in-difference) between Reason and the Imagination. This is a harmony/unity through agonism, rather than resolution.

The Dynamically Sublime and Mathematical Sublime are two important concepts to understand; the Dynamically Sublime describes the judgment of nature as a power deriving from the need for autonomy or subjective freedom. Between the Mathematical Sublime and the Dynamically Sublime, there is a transcendence through agonism, which involves a pleasure in pain (linked to Freud, in heading 22). The dominating, grandeur and overbearing forces of the Dynamically Sublime and the Mathematical Sublime, results in Reason's transcendence into the supersensible. This transcendence comes from the mind itself, as a form of mental elevation from the normal senses, and towards the pursuit of a higher purposiveness (through the introduction of the idea of infinity). This is the already expressed idea that Hegel put forward in his embodied logic, being that the transcendent is intrinsic/immanent to the materialist position (6). Importantly a Sublime judgment reveals the minds own purposiveness, which is not tied to objects, but rather being the minds very own subjective purposiveness (2 and 17).

The introduction of the idea of infinity, enables for a transcendence through resistance, thus providing for the power to overcome overwhelming sensations of the Dynamically and Mathematical Sublime. The infinite is not something which can be described (indeterminate concept of Reason), but only circumscribed through failures (hence the pleasure in pain aspect). The Sublime thus can only be circumscribed through failures; it is a failure to define the Sublime, that is the definition of the Sublime itself, and this pain (the failure to describes/define/identify the Sublime) is itself a fruitful failure thus resulting in pleasure-in-pain. In this way, Kant demonstrated that the only way to define essence, was paradoxically the very failure to define essence (co-incidence of opposites once more).

xviii. The Owl of Minerva (20) and An open future and "the end of history" (21)

This heading demonstrates that rationalizations are retroactive constructions of the past, which impact the present and future. These rationalizations are products of efficiency, given that they involve reductions of information, for memory efficiency (1). Moreso, the importance of meta-freedom, being qualitative freedom is discussed (1,2, 11 and 23). This is the freedom to reconfigure or re-write concepts/rationalizations themselves (the semantic content of concepts). Without this ability, a closed society/system results, which is one without freedom or autonomy (23). This is a nihilistic system/society, which is involves a denigration of embodied health (23).

A re-interpretation and justification of Hegelian logic is then presented. Herein, it is demonstrated that Hegel is the key thinker of a radical openness, which protects subjective freedom. Hegel does this, by demonstrating that nothing is fundamental, and everything is emergent, based on subjective actualization (13-16). It is precisely because nothing is fundamental, that anything is possible. The subjective aspect arises from Hegel swopping the logical categories of contingency and necessity, thus demonstrating that necessity is an instance of subjectively realized retroactive contingency.

Moreso, the Hegelian idea of aufhebung, or the co-incidence of opposites (also the logic of chirality), demonstrates that freedom/free-will requires a plan/fate/determinism. In other words, that which seemingly restricts freedom, is the a priori for the possibility of freedom itself. This links precisely to explanation closure axioms, which code-in their own incompleteness- that is a form of determined-indeterminateness/non-determinateness (the incomplete Absolute again in heading 10).

xix. Todestrieb: Saving the Death-Drive (22)

This heading serves to validate Freud and Hegel too) and demonstrate that several Freudian ideas (which Freud constructed after having read Hegel).

Firstly, various conceptions of the death-drive are validated (and made equivalent) with empirical evidence from the sciences. This includes the death-drive being a mechanic of true freedom/subjectivity, which is a common theme throughout this manuscript.

Secondly, the conception of the Absolute involving the Absolute being an instance of a unity-in-difference/failure to unify (which is Hegelian aufhebung), enables for true-freedom and open systems/contextual import. The aforementioned is necessary for adaptation, and evolution (12) given that non-linearity is introduced (17).

Thirdly, the death-drive is an instance of obtaining pleasure beyond pain/ or pleasure from beyond the pleasure principle (19). The aforementioned is the logic of antifragile systems (2, 17 and 19).

Fourthly, the death-drive seeks out subjective possibility and freedom, which is linked to the Kantian infinite judgment. The death-drive is equivalent to the action of the intentional misrecognition (6, 20, 21), which thus renders a system open, and is an instance of true freedom.

xx. Conclusion: A brush with the Absolute? (23)

The conclusion ties together all headings and describes a true brush with the Absolute (explanation closure axioms) as being a brush with true subjective freedom. This subjective freedom arises in three ways: (a) the gaps that constitute explanation closure axioms (the complete-incompleteness) and (b) the ability to interpret/constitute events as such and (c) the ability to negate non-existing events (or not constitute them), thus rendering a system open. The last instance, (c), is a subjectively induced-gap within the physics of the system of reality (13 and 16).

It is precisely because of the existence of these gaps, that an indirect proof can be constructed to demonstrate the existence of a plan, in a backwards fashion. The existence of such a plan is not contra-to-freedom because it implies determinism; the existence of a plan/determinism is the very a priori for freedom itself as described (20 and 21). It is this co-incidence of opposites, that is known as the logic of aufhebung (or the unity-in-difference).

To end off, the manuscript provides self-referential/self-aware commentary on academia, but also a commentary on the way in which society views and frames “philosophy.” Philosophy is central to all knowledge and disciplines because philosophy is a system of interrelation, known as logic, which can take various forms, being causation, synchronization and others. Philosophy is the system behind the creation of semantics/meaning/order. The denigration of philosophy in the present age, and the false assertion that philosophy is other than the natural sciences, only leads to a society that lacks creative

and critical thinking abilities. These are challenges which face optimism, openness and embodied health, given that embodied health requires meta-freedom, which is the practice of philosophizing, and re-philosophizing.

1 Freud

1.1 Thresholds and consciousness

Freud was influenced by his predecessors who were trying to establish when a perception crosses a threshold and enters consciousness. This threshold was linked to the intensity of the perception (like burning candle away in the distance, you can see it as it comes closer to you only). Thus, the question at the time was how much quantity of perception (or energy) was needed to cross a threshold into consciousness which is a qualitative experience. In this light, Freud thought something similar must happen with the unconscious and the consciousness. There must be a threshold in which stimuli awareness in unconscious processes become subject to consciousness.

When Freud (1915) published his breakthrough work, *The Interpretation of Dreams*, wherein he proposed that this threshold was not a stimulus threshold but rather a defensive threshold. Freud was suggesting that the psyche was trying to maintain a homeostatic stability against states of excitation. These defences were thus necessary to prevent against states of high energy or high energy expenditure. For Freud, this separation of was based on his economic theory of mind. Conscious and unconscious was separated because of the damage which excessive energy can do since through and ideas could be invested with energy. This investment was called the *besetsum* or the cathexis as it is more widely known. *The unconscious has too much energy*.

In *Beyond the Pleasure Principle*, Freud (1920) described the protective shield against stimuli as being a two-sided surface entity which is exposed on both sides. Thus, it faces internal and external forces (Johnston, 2008; Freud, 1920). While organisms can remove itself from sources of excitation and pain in its *Umwelt* (external); it cannot escape from *Innenwelt* sources of the same. This is why it is the id-body of the drives which forms part of primal repression (Johnston, 2008). Thus, Freud has perfectly described too the nature, functions, and limits of the membrane (below).

1.2 The return of the repressed

Primordial repression is that which opens up the temporal dimension; but is itself *eternal or atemporal*. Hence, unconscious is not the opposite of consciousness—but the unconscious describes the very founding gesture of consciousness itself (Johnston, 2008). This the gesture of choosing oneself, and creating a synthetic unity of the self (Žižek, 1996; Johnston, 2008). The unconscious then includes the primal repression (as divorce) and the *trieb*, as is *atemporal* (or, out-of-joint). The divorce as an act, creates both consciousness and unconscious; with the divorce then being absorbed by the unconscious which it produced.

To relate this back to Freud's economic mind; in *Inhibitions, Symptoms and Anxiety*, Freud speculated that primary repression must occur—due to early outbreaks of intense anxiety, which happen before the formation of the superego (Johnston, 2008; Freud, 1926). The prefrontal cortex (PFC) behaves as a superego, and it only fully matures in the mid to late twenties (Sapolsky, 2017). During this maturation, there is another process called neural Darwinism; wherein neurons must be die for proper, efficient functioning. Thus, Freud concludes that primal repression must come about because of quantitative factors such as excessive degrees of excitation and crossing the threshold.

The truth or the retroactive construction is based in the present-as-future; hence, it the truth is contingent on us writing (and re-writing) the past. We give the past its meaning and its truth based on current understandings; retroactively. Thus, the Freudian act of repression and the return of the repressed coincide; the repressed is itself the retroactive effect of its return (Žižek, 2012).

Trauma presents a good way to present this temporal paradox. A traumatic memory is one of the past—however, it appears within the present of an individual's experience, thus blockading the individual's future. Thus, trauma is a "point of view" returning from the "future" with the aim of reconciling its own understanding and meaning. From the perspective of the "present", the trauma event is in the past. This trauma is also within the present, as it is experienced within the present. When the present tries to deal with this trauma—the present must position itself as the "past (of the trauma event)—which renders the present, "the future" within this reconciliation. This reconciliation involves creating a narrative of events of the past (to create the trauma event), from the present-as-future. Hence, this is a retroactive narration, and creation of a past event from the perspective of the future (as present).

1.3 **Neural transmissions**

At the individual neuronal connection level, the structure of learning and prediction is possible due to the action potentials (axonal spikes) in neurons which is an electrical signal. Neurons are connected to one another through axons and dendrites; the point of connection between an axon and dendrite is called a synapse. Thoughts and perceptions are determined by these spikes (Hawkins and Dawkins, 2021). The synapses are responsible for memory. *Memory is a product of sequential pattern activation in synapses of many neurons* (Hawkins and Dawkins, 2021).

Prediction includes one's own movement in every part of the body and predictions of one's own movement in the world. In terms of sequential activation, this is possible through *dendrite synapse* spikes. After receiving input, the distal synapse spike occurs when other dendrite spikes occur close-by and travel to the cell body priming the cell for activation (a predictive state) by raising its voltage (Hawkins and Dawkins, 2021). This is known as *neuronal oscillation*. The mini cortical columns' response to the same input patterns and *unexpected inputs cause more firing than expected ones* (Hawkins and Dawkins, 2021). When an input is predicted, then only the predictive state neurons fire. Therefore, prediction works by pattern formation and activation in the brain through both kinds of synapses. It is the neurons within the brain that recognise patterns of activation, thus determining when it too should activate through dendrite spikes. This is the common cortical algorithm (Hawkins and Dawkins, 2021; Hawkins and Ahmad, 2016). *Prediction happens inside neurons themselves* (Hawkins and Dawkins, 2021). The predictive state neurons *will fire*, and the others will be inhibited.

Firing of neurons means the transmission of nerve impulses through sending out electrical impulses. Activation of neurons happens as per the predictive model above. On a more technical ground, the predictive modality is based on stimulation from light, chemical information, or heat from surrounding cells or membranes. Different types of neurons require different kinds of stimulus to fire. The fluids inside neurons are separated from the outside by a polarized membrane which contains electrically charged ions.

The brain processes signals and time in the following way. At the more granular level, it is at the neuronal level there is a temporal element in the electrical signals between individual neurons. At this level, neurons fire with the use of sodium ions and calcium. Dendritic action potentials for example occur when voltages are spiked (priming) using positive ions (when calcium is used it is called dCaAPs) (McRae, 2023). Neuronal signals operate in a wave-pulse form wherein the openings and

closings of channels exchange these charged ions (sodium, chloride, potassium and now calcium too). The pulse is the flow of these ions, an action potential. These pulses are communicated in three ways: (A) AND; (B) OR; and (C) EXCLUSIVE OR. (A) stipulates that if neuron (X) AND neuron (Y) are triggered then the message is passed one. (B) stipulates that if (X) OR (Y) is triggered, the message is passed on. (C) permits a signal ONLY IF another signal is graded in a specific way. (C) is the relatively new (dCaAPs) signal pathway discovered (McRae, 2023).

When neurons reach the neural threshold (anything above causes firing, anything below does not), depolarization results (ScienceDirect, n.d.). This is a change in the cell's potential. "Potential" refers to *differences in electrical charges*. There are two types of potentials: (1) the rest potential; and (2) the action potential (as above). The neural threshold must be reached before a neuron can go from a rest to an action potential. The firing, which occurs after the threshold has been reached, means that the membrane's permeability is changed. By polarizing the membrane, a change of electrical charges (going from negative to positive) is enabled, which runs along the entirety of the cell membrane. After this, the neuron returns to its resting potential. This means that neural transmission is *determined by the threshold and whether the stimulation meets that threshold*.

Whether the threshold potential is reached is determined by the amount of charge which is transferred across the membrane (ScienceDirect, n.d.). The threshold is determined by the neuron and not the stimulus. The parameters for the stimulus within the threshold relate to strength and duration of the stimulus. *If the threshold is reached, the amplitude of the resultant action potential is the same, regardless of the level of stimulation*. This relationship between levels of stimulation and the production of impulses is called the *all or none principle* (Platkiewicz and Brette, 2010).

The action potential depends on the cell membrane permeability (a characteristic of the cell and not of the strength of the stimulus which triggers it) (ScienceDirect, n.d.). *The greater the duration of the applied pulse, the smaller the current intensity required to excite the fiber* (ScienceDirect, n.d.). However, a current can be applied for an infinite amount of time, but if it is below the threshold value, it will not cause the firing. There is also a minimal stimulation time required—which is the shortest duration of stimulation which is capable of producing excitation (even if massive currents are applied) (ScienceDirect, n.d.).

Membrane impedance is high when in the presence of high frequencies (alternating current (AC), for example). The higher the frequency, the less membrane impedance (resistance to transferal of energy per unit charge) results—and thus, the potential difference which can be produced across the cell membrane is low (ScienceDirect, n.d.). *What this means is that alternating currents of high frequencies has a smaller tendency to electrocute, and the energy of these currents can be dissipated as body heat* (ScienceDirect, n.d.). The higher the frequency of electromagnetic waves means that the wavelengths are smaller. Conversely, higher frequencies also means that it carries more energy. This is important; in plainer terms, we can say that the higher the frequency of the AC, combined with a very short duration in the alternations, is safer for the cells to transmit charge and (radiate) dissipate heat.

The question is then: On what is this threshold value based, and does it vary? One argument is that the value is determined in a non-linear fashion, namely it can be a combination of many variables including membrane potential and complex features of inputs (Platkiewicz and Brette, 2010). *This means that it can depend on preceding rates of depolarization and preceding inter-spike intervals* (Platkiewicz and Brette, 2010). Studies have also indicated that the threshold can adapt to slow changes in the input characteristics (Platkiewicz and Brette, 2010). The variation too depends on the

neuronal cell types and distances to the soma (Platkiewicz and Brette, 2010). In terms of spike threshold variability, it was found that a threshold formula provided an instantaneous time-varying value which agreed with the traditional statistical models (Platkiewicz and Brette, 2010). The sodium voltage gated channels which are constitutively responsible for cell excitation *mediate a positive feedback mechanism which produces the constitutively necessary instability required to initiate actions potentials*. Importantly, it was also determined that the *threshold for the Na activation gate depended on Na inactivation and conductance's* (Platkiewicz and Brette, 2010). This can explain the *effects of preceding spikes and membrane potential histories on cell excitability* (Platkiewicz and Brette, 2010). In other words, the threshold variability value is dependent on Na inactivation value. Thresholds are also inversely correlated with previous inter-spike intervals, namely if the inactivation time constant is long in comparison to the inter-spike interval, it is likely that the threshold would be linearly correlated with the firing rate (Platkiewicz and Brette, 2010).

Once the neuron has fired, it enters into a bi-part-phase called the *absolute or relative refractive period* (Bielajew et al., 1982). For a short period of time after firing, the absolute refractive period means that the neuron will not fire even if the stimulation threshold is reached or if there is massive stimulation. The relative refractive period allows for firing but only if the stimulus received is stronger than the threshold. After this period passes, the neuron will enter the rest potential and be able to fire "normally" once more. The more failure (in the instance of predictive error) the more neurons will fire and thus a heightened stage of consciousness arises (Hawkins and Dawkins, 2021).

On a granular level then, this is a retroactive constitutive negativity. The retroactive constitution is based on presently non-existing events or things (which may have existed in a particular state in a past time) which then shapes the "present". The negativity speaks to a negation—namely setting of a threshold. History moves forward!

1.4 The unconscious

On this, Naidoo (2023c) says:

> "When Freud said "Wo es war soll ich warden" ("where it was, shall I be") he was referring to the alienation of a specific aspect of oneself, namely the unconscious (Strachey, 1964). Freud wanted the unconscious (as substance) to be understood as subject too (Žižek, 2012). Thus, one of the goals of psychoanalysis is to enable the subject to realize that it is his own repressions that gives the appearance of content formations within the unconscious.
>
> Freud also drew a distinction between repressed content and the form of repression itself (Žižek, 2012). The key difference here is that the form of repression continues even after any repressed content is no longer being repressed. If the subject can free the repressed content, the repression itself stays behind in its form. As with Freud's conception of dreams, it is the form itself which propagates a feeling of a hidden content kernel of truth. The movement from Kant to Hegel explicates this perfectly. Briefly, Hegel's argument was that predictive reason, or apperception, creates the idea of essence as being behind appearance. The distinction between form and content is the same (Žižek, 2012). The distinction between form and content is a distinction within content itself (as the distinction between essence and appearance is within appearance itself).
>
> The Freudian unconscious is thus the primordial repression. However, it goes further than this. The primordial repression (Ur- Verdrängung) persists because the repressed content does not pre-exist the repression; it is rather retroactively constituted by the process of repression. It is like the notion that the law itself, through its criminalization of certain acts or ethics, can make the criminalized content

> desirable through its very repression. Primordial repression is not a repression of some content into the unconscious; it is rather the repression itself which constitutes the unconscious (creates it)".

The goal of psychoanalysis is to bring the subject to the understanding that *it is their own repression* which constitutes the unconscious. It is a repression of *nothing.*

1.5 Repetition, memory, and transcendence

As Sapolsky (2017) has pointed out, the prefrontal cortex is an honorary member of the limbic system whilst also being part of the cortical system. It also acts like a "superego" in decision making and rule development. Furthermore, the regions of the PFC are responsible for creating state spaces through stochastic reasoning (the subjective "how would I feels if x happened"). Evolution too functions according to creating redundancy in preparation for failure (Nowak et al., 1997). Additionally, memory also functions according to repetition. In 1954, Brenda Milner discovered that the medial temporal lobe and the hippocampus mediate declarative (explicit) memory storage, which is a conscious memory for people, objects, and places (Kandel, 1999). In a follow up study, she discovered that the same subject of her previous study was not able to consciously recall new memories about people, places of objects; but that he was still capable of learning new perceptual and motor skills (Kandel, 1999). This is known as procedural or implicit memory, and these are completely unconscious and are only observable in performance, not conscious recall. *Constant repetition which can transform declarative memory into procedural memory* (Kandel, 1999). Learning new rules requires conscious activation of the PFC to learn and abstract new rules. However, after time, these rules are stored and become a nonconscious "knowing" or activity. Learning to drive is an example—one is still thinking when one drives; just not consciously. Procedural memory is a collection of processes involving different regions and systems in the brain including recognition of stimuli, sensory regions, cued emotional states of the amygdala and the basolateral amygdala and others including processes in the cerebellum and the explicit system of the hippocampus (Kandel, 1999).
