# Supplementary material for: Open optimism as an “embodied-health” ethic for the information era
Source: Front Pharmacol. 2024 Jun 17;15:1331237. doi: 10.3389/fphar.2024.1331237 (PMC11215117; doi:10.3389/fphar.2024.1331237)
Supplement: Supplementary file 11 [file DataSheet5.pdf]

Supplementary Appendix

Open-optimism as an “embodied-health” ethic for the information era

1 Content hidden in form

Naidoo (2023c) says:

> “Hegel highlighted the fact that Kant did not account for the immanent content of the categories themselves, as he instead posited that the categories were devoid of any content. What Hegel is alluding to is the hidden content of the form itself. This immanent hidden content is that which constitutes the categories themselves. This content is imperceptible.
>
> Each category is linguistically dependent on and related to other categories, since categories must oppose one another for them to qualify as such (Williamson, 2016). Thus, Hegel suggests notes that those categories are not exclusively within the Kantian transcendental subject, but they constitute both the noumenal and phenomenal worlds. Hence, the categories’ structure, thought, and objects – as the supersensible presuppositions/concepts for all phenomena. As each category is dependent on others, each category is self-contradictory, which allows for one category to transition into another (Williamson, 2016). Thus, things become what they are not. This also means that the supersensible beyond, also contains self-contradiction. Thus, Hegel’s solution is one different to Aristotle, Plato, and Kant – in that it is the logical categories which are located both outside the finite/empirical/sensual world, and within it, simultaneously, constituting both”.

Continuing, Naidoo (2023c) says:

> “Freud had similar insights to Hegel. His thesis on an *Interpretation of Dreams* (Freud, 1915) revealed that when we consider dreams, we often think that there is some intrinsic hidden kernel of meaning within them that reflects some unconscious truths or desires about ourselves/out state of mind. Freud reversed this; he argued instead that there was no meaning in dream-content themselves, but that we should rather question why dream-thoughts took the form of dreams in the first place. *Freud was arguing that there is no inherent meaning hidden within the dream,* rather he was seeking the necessary conditions for this illusion to take place. *What is it about the dream-form that makes us think there is something hidden content within dreams?* Thus, dreams lie to us since they mask themselves as hiding a truth about ourselves. Freud insisted that “*there is nothing unconscious in the latent dream thought*” (Žižek, 1989). For Freud, the latent dream thought is composed of thoughts which are prevalent during consciousness, which can then be intentionally displaced into the unconscious. What constitutes the latent thought aspect of dreams is then not the thought itself, *but the work in which there is displacement and figuration of the contents of words and syllables which gives the dream its form as a dream* (Žižek, 1989). Thus, dreams do not reflect any unconscious hidden meanings, nor do they represent any hidden sexual desires; these are present during conscious or pre-conscious thoughts.
>
> These conscious thoughts are displaced into the unconscious (thus, making it seem like they originate there) due to an antagonism between it, and another desire operating within the unconscious. This unconscious “*desire has nothing to do with the latent dream thought”*. Freud (1915) says that normal thoughts of the conscious or pre-conscious is displaced into the dream-work mechanisms of the primary processes if it is painted with an unconscious wish which comes from infancy (and is repressed). This desire, for Freud, is a sexual desire which is repressed (*urverdrangung*); and it is so because it has no symbolic representation in normal everyday language (Žižek, 1989) (hence, it cannot be represented in

consciousness or everyday language), thus it can only operate within the primary process. It is thus unconscious desire which then attaches itself to the dream-thoughts, *which then creates the form of the dream. Hence, the unconscious desire is not hidden within the dream-content, it gives it its form as dream-form (it is appearance and not essence)*. Thus, there is always a triad at work; (1) latent dream text; (2) latent dream content; and (3) the unconscious desire (Žižek, 1989). *This unconscious desire is the real subject of the dream – not the content, but the form.* The unconscious desire then desublimates itself onto latent thoughts, creating a disguised form of a hidden kernel of content. Freud (1915) says: *"The form of a dream or the form in which it is dreamt is used with quite surprising frequency for representing its concealed subject matter".* Freud (1915) also says:

> "…But now that analysts at least have become reconciled to replacing the manifest dream by the meaning revealed by its interpretation, many of them have become guilty of falling into another confusion which they cling to with an equal obstinacy. They seek to find the essence of dreams in their latent content and in so doing they overlook the distinction between the latent dream-thoughts and the dream-work. At bottom, dreams are nothing other than a particular form of thinking, made possible by the conditions of the state of sleep. It is the dream-work which creates that form, and it alone is the essence of dreaming - the explanation of its peculiar nature".

Ultimately, Freud's process is two-fold: (1) he says that dreams must be understood to be meaningful which transmits a repressed message; and (2) the repressed message, however, is not in a hidden kernel of content, but in the form of the dream itself (Žižek, 1989)".
