# Supplementary material for: Open optimism as an “embodied-health” ethic for the information era
Source: Front Pharmacol. 2024 Jun 17;15:1331237. doi: 10.3389/fphar.2024.1331237 (PMC11215117; doi:10.3389/fphar.2024.1331237)
Supplement: Supplementary file 12 [file DataSheet12.pdf]

Supplementary Appendix

Open-optimism as an "embodied-health" ethic for the information era

1 Symbols

Scaffolding is the process of integrating incoming information with extant knowledge structures. It can have diverse effects on human judgement and behavior. It is the passive natural process through which new concepts are formed mostly in early childhood. During scaffolding, features of abstract concepts are mapped onto existing well understood concepts in such a way that the structure of the developmentally earlier primary concept is retained in the newly constructed concept. This structure imbues the newer concept with meaning. When an abstract concept is scaffolded onto a foundational concept, these concepts become associated much in the same way semantically related concepts are naturally associated in the mind. These scaffolded concepts are unique because of how their associations are formed.

Focus here is on the processes born from functional interactions with the environment, scaffolding processes are endemic of the general character of human thought—humans often use the structure inherent in fundamental aspects of their physical worlds to develop higher level concepts. Thus, scaffolding processes simultaneously broaden the scope of human thought—whilst tethering those thoughts to physical environment in which they occur. Concepts like time, temperature, and distance along with physically based goals highlight the processes by which sensorimotor resources can structure higher-order cognition.

When developing spatial language and concepts we first must perceptually understand these concepts which is constrained by the physical properties our perceptual tools—namely the body and environment. For example, we have come to associate "forward" and "upward" as being good because most perceptual organs are located on the "front" of the body. There is thus more information gain. The upward perception is unbounded, while the downward perception is blocked by the ground—hence down is bad. It has been shown that spatial orientations affect understanding of temporal relations (Boroditsky, 2000; Boroditsky and Ramscar, 2002)—which highlights the significance of time as an early scaffolded concept.

2 Symbols and metaphors

Symbolism and communication go hand-in-hand—and it was used by ancient humans as it is used by modern humans. It is used by other animals too, including monkeys (Livingstone et al., 2010; Sapolsky, 2017; Grouchy et al., 2016). Language is an example of useful symbolism. Language is a metaphor, and it is just the ability to transfer information from one to another; a metaphor taken too seriously at times. Friedrich Nietzsche (1896) was another to point this out. On language, Nietzsche (1896) says:

> "… a uniformly valid and binding designation is invented for things, and this legislation of language likewise establishes the first laws of truth. For the contrast between truth and lie arises here for the first time. The liar is a person who uses the valid designations, the words, in order to make something which is unreal appear to be real. He says, for example, "I am rich," when the proper designation for his

> condition would be “poor.” He misuses fixed conventions by means of arbitrary substitutions or even reversals of names”.

Nietzsche is effectively pointing out that truth is being confused with the metaphors of limited symbolics such as linguistics, culture, and tradition. Thanks to language, we can separate symbols, which is the message, from meaning (the *feels*). We are also able to represent past and future information because of this separation which increased over time. We can also communicate information that is outside the realm of meaning or reality—namely the external world, space, the universe, and even the process of lying. The highest form of symbolism is the metaphor—which exists in all shapes and sizes and represents different things.

However, in our uses of metaphor, we often confuse them with what is literal. But why? There are three main reasons for this. The first is that the neurobiological capacities which enable us to do this are very recently evolved. When we try to decipher between what is metaphorical or what is literal, we often confuse them both. For example, the anterior cingulate cortex (ACC) (which gives meaning to pain through prediction—namely there should be no harm/sensation, but there is, therefore here is pain for you) mixes up physical pain and psychical pain (Sapolsky, 2017). When humans are sad or feel alienated, the ACC fires and pain receptors elevate. This applies to the activation of the insula in response to situations of disgust—including moral disgust. Moral disgust can, and does, have physiological impacts.

The second is that metaphors allow for activation of the dopamine reward system which creates motivation for the carrying out of actions (thus, we created a metaphorical reward system). The third reason is because of political ploy. Individualism: namely confusing a metaphor with a literal is a great political ploy. It is a means by which meaning is given to lives in exchange for political support. Controlling metaphors is a great way to control humans (Lakoff and Johnson, 1980; Lakoff, 1996; Macpherson, 1962).

Feelings are another example of a process of misrecognition, or mix. For Chalmers (1996), the subjective experience (*feeling*) has risen to the status of symbol. Feeling is often confused with emotion, behaviour, and mental states. Injury is an emotional behaviour while pain and hurt would be feelings. However, since some animals do use symbolism—it is likely that they have feelings too! Perhaps then, *there is something like it is to be a bat* in Chalmers or Nagel parlance (Nagel, 1974).

In *On Truth and Lies in a Nonmoral Sense*, Nietzsche (1896) argues that *all knowledge* is simply a human construct—including morality. It is a metaphor, which exists as we understand it only within the human symbolic meaning. Nietzsche (1896) says:

> “Once upon a time, in some out of the way corner of that universe which is dispersed into numberless twinkling solar systems, there was a star upon which clever beasts invented knowing. That was the most arrogant and mendacious minute of “world history,” but nevertheless, it was only a minute”.

David Deutsch is another who pointed this out in *The Fabric of Reality* (Deutsch, 1997), and *The Beginning of Infinity* (Deutsch, 2011). In both books he argues essentially that humans have produced their own virtual realities and that every shred of knowledge or reason comes from this. Knowledge is a human symbolic.
