# Supplementary material for: Open optimism as an “embodied-health” ethic for the information era
Source: Front Pharmacol. 2024 Jun 17;15:1331237. doi: 10.3389/fphar.2024.1331237 (PMC11215117; doi:10.3389/fphar.2024.1331237)
Supplement: Supplementary file 13 [file DataSheet8.pdf]

Supplementary Appendix

Open-optimism as an "embodied-health" ethic for the information era

1 Negation and prediction

Naidoo (2023b) says:

> "Inferential logic can be atemporal if properly considered. There are two types of inferences – type one inference indicators and type two inference indicators (Colorado, n.d.). Type one indicators refer to "therefore", "consequently", "thus", "implies" and others of this sort. The quality of these inferences is that the logical flow is temporal; the steps entail a movement from the premises to the conclusion. However, type two inferences do not do the same. These include, "since", "because", "for", "is entailed by" and others. The quality of type two inferences is that the conclusion is then followed by the type two inference and then the reason. For example: Hegel was a more popular thinker than Schopenhauer *because* students flocked to his class instead of Schopenhauer's.
>
> In this example, "because" is the type two inference indicator, and it indicates that what follows it (students flocking to Hegel's class instead of Schopenhauer's) is the reason for believing what comes before the indicator (Hegel was a more popular thinker than Schopenhauer). In other words, this is abduction or hypothesizing, which is also the Hegelian notion of *setzung der voraussetzungen* – which means to posit the presuppositions. I would strongly suggest that abduction/type two inferential logic accounts for actual change in evolution. Hypothesizing is atemporal logic and it also speaks to a general notion of statistics (what kind of system best fits the data I have at hand; instead of trying to predict data from the system one has).
>
> There are also the concepts of direct and indirect adaptation when thinking about linguistics – which can also be applied to any evolutionary theory of information including biology. First, there is a distinction between primary functions and evolutionary origins. For example, the wings of an eagle can be understood in terms of its primary function of flight. However, the evolutionary origins of wings on the other hand is not flight; but rather came about as an increase of surface area (and hence increase in volume) for better thermal regulation of bodily heat (Sbardolini, 2022). It is still used for this function today by some. It was only afterwards that the benefit of flight were exploited by organisms. Thus, a feature or behaviour of an organism does not account for its origin, but in some cases it can. Thermoregulation would be direct adaptation in development; indirect adaptation would be the exploitation of flight in development (Sbardolini, 2022). Negation too can be direct (determinate) or indirect (indeterminate) in the same way. Both positive and negative information are both useful and beneficial (namely, "do that!" But also, "Do not do that!"). Consider the following on the origins of negation –
>
> > "The markedness of negation must have its source outside of language if it is to be of explanatory value: it could be mental or wordly. If the markedness of *not-A* is cognitive, we get the direction of causality backwards: the sentence *not-A* may well be more complex and harder to process than *A*, but this is because the presence of negation leads to complexity, not the other way round. After all, information that *Every student is asleep* is not more complex than information that *Some student is awake*, even though the former is equivalent to *No student is awake*. Psycholinguistic evidence has established a correlation between the use of negation and higher cognitive complexity (Dudschig et al., 2021), but this is not because negation signals a higher complexity in information that was already there to begin with, independently of our use of

> negation to convey it. Alternatively, the inherent markedness of *not-A* is metaphysical, and comes from a special property of Negativity with which some facts are endowed" (Sbardolini, 2022).

While negation is typically understood as communicating negative information (when something is not the case) or when something does not exist or is absent (Sbardolini, 2022), these do not explain its origin still. Whilst some have postulated that negation is an adaptation, it is more likely that negation is an indirect adaptation. The signal game can be used to explicate this. In the rejection game, there are two communicatees. The speaker relays facts about the environment, and the listener must then decide whether the communication is truthful by way of rejecting (ignoring it) it or accepting it (responding to it). If it is ignored, than the communication of information fails. The rejection game makes a generalist modification to the methodology; rather than accept or reject, the listener can either accept or refuse to accept. Given the setup, the speaker and listener are in a noisy channel; it is best for them to avoid (1) using more than one signal for one informational state (synonymy) and (2) the use of one signal for more than one information state (ambiguity). Thus, the speaker cannot have a signal for each bit of necessary information (too costly energy wise) nor can the speaker have too little symbols since this will result in ambiguity and thus potential rejection (Sbardolini, 2022). Negation is the solution to this issue since it allows the speaker to introduce a new symbol at a small cost. Negation enables the speaker to express in language the choice which the listener must make between refusing to accept or accepting. Negation in this light is like an additive flag to a signal to indicate that not-A is to be accepted if its counterpart A is rejected (and vice versa). The speaker can now avoid initial ambiguity which is present *when communicating in finite language over an infinite information space*. The *negation thus gives a preliminary choice to the speaker in terms of how to express him/herself*. However, this avoidance of synonymy and ambiguity is not sufficient to explain negation in full.

Since the listener cannot choose to both accept or to reject (failure of acceptance and not acceptance of the opposite) (Sbardolini, 2022) we can account for the incompatibility of both terms by reference to attitudes. Attitudes are one way in which a speaker can convey disincentives (such as there being a norm violation attributable to one line of action). If there is an expectation related to the choice, then the speaker has a motivation to recognise the difference between both A and not-A (Sbardolini, 2022). Hence, if these symbols are used synonymously then the result is that there is a sanction (so there is a disincentive to use them synonymously). It is in this light that ambiguity can be removed and information can be communicated efficiently. Thus, negation enables (1) expressive power of the language (signal number matches the number of informational states) and (2) marks incompatibility between the pairs of A and not-A. This is how it evolves. The first is about negative information which is important, *but this function comes after the second function*. This is the same logic as the wing described above. The second more explicitly relates to negation as a model of *compositional development*. This means that the negation speaks to *not contrariety or absence but instead similarity* (Sbardolini, 2022). The contrast between A and not-A comes from the opposition of acceptance and rejection; negation does not derive its meaning from information states themselves but rather negation derives its meaning of incompatibility of acceptance and rejection itself. This is a pure difference based on frequency and its role in communication (to exclude the more common understandings in favour of the less common). The not-A is less common than the A, and thus will be rarer (in this way the not-A evolved as a means to correlate the more frequently occurring informational states with those which are rarer). It is thus a device for the speaker to use in which he can mark the difference between accepting or rejecting communication in a large informational space with finite language.

This form of negation is hence an indirect adaptation in that it gave the speaker an advantage of being able to express the difference between acceptance and rejection. This allows *the speaker to anticipate the choices of the listener and avoid ambiguity*. The speaker in this way can stay ahead of the game of communication in contexts wherein the listener can accept or reject thus overcoming many issues mentioned. In this way, it *is self-referential and also embodied knowledge since it ties relative common understandings of similarity.* This can also be known as a teleological negation – since the in its corresponding Greek form, it corresponds to "to see (what is) coming." (Malabou, 2005). In other words, to *predict* by positing one's own presuppositions".
