# Supplementary material for: Open optimism as an “embodied-health” ethic for the information era
Source: Front Pharmacol. 2024 Jun 17;15:1331237. doi: 10.3389/fphar.2024.1331237 (PMC11215117; doi:10.3389/fphar.2024.1331237)
Supplement: Supplementary file 14 [file DataSheet10.pdf]

Supplementary Appendix

Open-optimism as an "embodied-health" ethic for the information era

1 Reciprocal self-constitution

Naidoo (2023b) says:

> "Unlike the subjective idealists like Descrates or Kant (Anon, 2023e), Hegel does not locate the Ideal in the human mind. In his conception of the Idea, Hegel includes material objects and finite entities. Hegel (1969) says –
>
> > "The proposition that the finite is ideal [ideell] constitutes idealism. The idealism of philosophy consists in nothing else than in recognizing that the finite has no veritable being. Every philosophy is essentially an idealism or at least has idealism for its principle, and the only question then is how far this principle is actually carried out."
>
> On the finite, Hegel describes this concept as not only variation or alteration – but also as something which ceases to be (Hegel, 1969). All physical things for Hegel are those which will cease to exist. Hence finite expands to all material or physical things, not just humans. These finite things are dependent beings, and thus not fully real. Thus, the finite must depend on an infinite – however what is this infinite? For Hegel, it is not God per se, but instead each finite being is in some way an aspect of the Idea. Hence, the finite entity itself does not correspond to its concept per se – and this is its inherent limitation which results in its own finitude. The Idea is thus the infinite, of which finite beings are part of. Things which are real, or infinite are those wherein there is a correspondence between the concept and the object (or entity itself). Reality is thus conceptual, and the finite is as nothing (Moyar, 2017).
>
> > "The true is the whole. But the whole is nothing other than the essence consummating itself through its development" (Hegel, 1977).
>
> The non-finite (as infinite) arises through the process of the finite negating itself because of its own differential constitution (incompatible parts) (Moyar, 2017). This means that the "essence" or rather the movement of the finite is to negate itself into nothingness; however, this would leave the non-finite as correlatively also being nothing. For there to be the non-finite, *the finite must subsist*. Since the finite has "nothing" as its content, this means the content of the non-finite would also be "nothing." The relation between both is based on *finitude itself*. This must mean then that then that the non-finite must be the same as the finite since the non-finite arises through the finite self-negating itself. However, this means that the non-finite must posit the finite to ensure that it can exist itself. It is an oscillation of co-constitution between both the non-finite and the finite. This is what Hegel called the *spurious infinity* or the bad infinity (Moyar, 2017).
>
> When Hegel analyses the essential determination of the finite – he finds that it tends to pass beyond its limits (the ought – which is the tension between somethings determinations and its modifications) and hence it negates itself; creating the infinite. This infinite though is limited by the finite too; it is in fact negated when it is posited (Moyar, 2017). The infinite is that which is *beyond the finite* and hence it is also limited by the finite (it is the non-finite). Hence a limitation of this sorts, makes the infinite itself finite – hence it must pass beyond its own limits into the finite. In this light, the infinite too has limits and is subject to the "ought." The limits of the infinite must then too be surpassed (the ought) by passing over into the finite and constituting it anew (Moyar, 2017). This movement of passing beyond limits –

or negating oneself is how both the finite and the infinite reproduce themselves. By passing beyond their own limits, or self-negating, they reproduce themselves/self-reference/self-affirm themselves. However, this is only complete with the return to self. Both the infinite and the finite constitute a single, identical movement of returning to self; this is becoming. Becoming is the process of alienating oneself, and then returning to oneself (Moyar, 2017).

This means that the infinite is not the negation of the finite, and vice versa. They are not the negation of each other; they are instead the negation of themselves (self-mediation) which results in a renewed positation, reproduction, or reaffirmation of themselves. This is the self-negation, or the negation of negation. Thus, the true infinite is that which returns to itself, or self-relates. Hence the infinite when self-negating (differing form itself) exhibits the otherness which constitutes being – which means fundamentally that the infinite is not an ethereal inaccessible entity which is beyond existence, but rather something which is in existence itself and accessible (Moyar, 2017). The old concept of finitude which was based on a single negation thus falls since the old conception makes infinity forever beyond access.

Hegel placed the infinite within the experiential realm of the finite – in the same way that Hilbert (1983) described. Namely, through thought or reason. This also precisely expresses the relations of the fermion and the vacuum. The dipole-dipole forces which structure the aggregation of matter and the like- *are non-local*. Moreso, matter aggregation is a process of trying to *overcome* the non-local forces (Anon, 2023f)".
