# Supplementary material for: Open optimism as an “embodied-health” ethic for the information era
Source: Front Pharmacol. 2024 Jun 17;15:1331237. doi: 10.3389/fphar.2024.1331237 (PMC11215117; doi:10.3389/fphar.2024.1331237)
Supplement: Supplementary file 15 [file DataSheet15.pdf]

Supplementary Appendix

Open-optimism as an "embodied-health" ethic for the information era

1 Quantum theory

1.1 The wavefunction and locality

Broadly, quantum theory uses non-commuting operators to extract observations from states. The *Heisenberg uncertainty principle* (HUP) (Uffink, 2016) is an axiom of quantum theory (Caltech, n.d.). All it means is that non-commuting operators are not simultaneously diagnosable—which, in turn, means there is no simultaneous eigenstates for all observables. In simpler terms, term uncertainty principle alludes to the fact that the very act of measurement violates the principles on which "systems" themselves are built. These principles in include the conservation principles of mass, energy, momentum, angular momentum, and charge—and the non-conservation principles of space and time (including gauge invariance, rotation symmetries and the like) (Rowlands, 2007). The HUP has often been confused with consciousness or the observer effect—which is partly Heisenberg's fault for explaining it in that way (Futura, 2012). The "consciousness" argument on a system has been debunked many times. Furthermore, the observer need not be a human—it can be a machine too. Measurement just analogous for interactions between quantum objects and classical systems.

Wavefunctions do not correspond to observable quantities. The wavefunction is an intermediary which we cannot observe through measurement. What we do instead is just compute the probability distributions (absolute values) for measuring other physical observables using it (Hossenfelder, 2018). *What is measurable is the probability distribution derived from wavefunctions* (Hossenfelder, 2018). After measurements, wavefunctions must not be updated so that the measurement has a probability of 1—thus is called the collapse or reduction of the wavefunction, and it is *instantaneous* regardless of distance (this means that it is *non-local in that its spread over time is not gradual but instantaneous*). Entangled particles are linked non-locally—thus, quantum theory has been described as extremely counterintuitive, obscure, or ugly to phrase it differently (Guryanova et al., 2016). All it is really is a conjecture about how quantum states come to be suppressed when interacting with environments with "large degrees of freedom" (namely measurements or observations) (Hossenfelder, 2018).

Quantum theory holds that information can never be destroyed; it can only become very mixed up to the point where it is practically impossible to recover (Hossenfelder, 2018). However, information is still preserved. Zeilinger conducted an experiment in the Canary Islands in which it was concluded that, to correctly describe particles, we must accept that they can be in two different states at once, meaning that particles do not have either of the polarizations, but instead had both before measurement (Minkel, 2007; Hossenfelder, 2018). In other theories, connections between two places means that there must be travel from one point to the other, which means that it is not instantaneous. This travel must scale to the limit imposed by the speed of light. This travel from point one to two in time is known as "locality". However, entangled particles in quantum mechanics demonstrate that particles can be coupled non-locally (Hossenfelder, 2018). However, no information is exchanged

between entangled particles because nothing can move faster than the speed of light. Hence, at the quantum level, entanglement and non-separability mean that coupling can take place non-locally.

2 The Copenhagen interpretation

A problem within the formulism of quantum theory includes the fact that its own axioms assume the existence of macroscopic objects. A fundamental theory ought to explain the emergence of macroscopic objects, not *assume* their presence (Hossenfelder, 2018). Schrödinger highlighted the issue of the emergence of the macroscopic world, wherein he described how atomic decay is in a superposition of both non-decay, and decay simultaneously, prior to measurement (the cat in the box). When measurement occurs is the point where the cat become either dead or alive. This point of "interaction" between quantum states and classical states, is characterized by a "breakdown" in the quantum state—known as decoherence (Bacciagaluppi, 2020). This interpretation is dubbed the classical Copenhagen interpretation, which is the standard but not widely accepted. Approach. Decoherence can be caused by many factors such as temperature. Decoherence is thus the movement from quantum states to normal probability distributions.

To elaborate on this—this is a quote by theoretical physicist Stephen Weinberg (Hossenfelder, 2018):

> "My own conclusion is that today there is no interpretation of quantum mechanics that does not have serious flaws. This view is not universally shared. Indeed, many physicists are satisfied with their own interpretation of quantum mechanics. But different physicists are satisfied with different interpretations. In my view we ought to take seriously the possibility of finding some more satisfactory other theory to which quantum mechanics is only a good approximation".

3 The many histories interpretation

There is another incredibly interesting school of thought opposing itself to the ugly Copenhagen model (and its practice of ignoring what happens inside the box) (Hossenfelder, 2018). This is called the many histories interpretation. This model essentially collapses the internal external dichotomy above, thus removing decoherence from the quantum theory. How it works is that a human is to be understood as a quantum system themselves. As time evolves, wave functions would have many terms in it—with each term having a description of observers. *The observers then think they are seeing something different—one would see a particle spin up, while another would see it spin down* (Hossenfelder, 2018). In this approach there is an eternal and infinite production of histories of the Universe. Weinberg notes how this theory *is not logically inconsistent—but it is repulsive*. In other words, as the recent blockbuster is named—*Everything Everywhere All at Once* (Wikipedia, 2023). The Universes *history* is continuously multiplied every time something happens. To quote Richard Feynman, "*no one understands quantum mechanics really*" (Carroll, 2019).

Supplementary Material

References

Adolphs, R., Tranel, D., and Damasio, A. R. (1998). The human amygdala in social judgment. *Nat.* 393, 470-474. doi: 10.1038/30982.

Alshak, M. N., and Das, J. M. (2022). *Neuroanatomy, Sympathetic Nervous System*. Treasure Island: StatPearls.

Bacciagaluppi, G. (2020). The role of decoherence in quantum mechanics. *Stanford Encyclopedia of Philosophy*. https://plato.stanford.edu/entries/qm-decoherence/ [Accessed February 6, 2023].

Badiou, A. (2006). *Being and Event* (translated by O. Feltham) New York: Continuum.

Barber, N. 2016. Why entrench? *International Journal of Constitutional Law*, 14(2):325-350. doi: 10.1093/icon/mow030

Bielajew, C., Lapointe, M., Kiss, I., and Shizgal, P. (1982). Absolute and relative refractory periods of the substrates for lateral hypothalamic and ventral midbrain self-stimulation. *Physiol. Behav.*, 28(1), 125-132. doi: 10.1016/0031-9384(82)90113-5

Bienertova-Vasku, J., Lenart, P., and Scheringer, M. (2020). Eustress and distress: Neither good nor bad, but rather the same? *Problems & Paradigms*, 42(7), 1-5. doi: 10.1002/bies.201900238

Boroditsky, L. (2000). Metaphoric structuring: Understanding time through spatial metaphors. *Cognition*, 75, 1-28. doi: 10.1016/S0010-0277(99)00073-6

Boroditsky, L., and Ramscar, M. (2002). The roles of body and mind in abstract thought. *Psychological Science*, 13, 185-189. doi: 10.1111/1467-9280.00434

Bowlby, J. (1962). *Attachment and Loss*. New York: Basic Books.

Breton-Provencher, V., Drummond, G. T., and Sur, M. (2021). Locus coeruleus norepinephrine in learned behavior: Anatomical modularity and spatiotemporal integration in targets. *Front. Neural Circuits*, 15, 1-11. doi: 10.3389/fncir.2021.638007

Burgess, M. (2014). Spacetimes with semantics (I): Notes on theory and formalism. arXiv [Preprint]. doi: 10.48550/arXiv.1411.5563

Caltech. (n.d.). What is the uncertainty principle and why is it important? https://scienceexchange.caltech.edu/topics/quantum-science-explained/uncertainty-principle#:~:text=Formulated%20by%20the%20German%20physicist,about%20its%20speed%20and%20vice [Accessed December 6, 2022].

Carroll, S. (2019). Even physicists don't understand quantum mechanics. *New York Times*. https://www.nytimes.com/2019/09/07/opinion/sunday/quantum-physics.html [Accessed February 23, 2023].

Chalmers, D. (1996). *The Conscious Mind: In Search of a Fundamental Theory*. Oxford: Oxford University Press.

Collier, L. (2016). Growth after trauma: Why are some people more resilient than others – and can it be taught? https://apa.org/monitor/2016/11/growth-trauma#:~:text=Post-traumatic%20growth%20(PTG),often%20see%20positive%20growth%20afterward [Accessed May 2, 2023].

Colorado. (n.d.). Philosophy 3480: Critical thinking. https://spot.colorado.edu/~tooley/LectureforExercise1Phil3480.html [Accessed February 3, 2023].

Constantinescu, A. O., O'Reilly, J. X., and Behrens, T. E. J. (2016). Organizing conceptual knowledge in humans with a gridlike code. *Science*, 352(6292), 1464-1468. doi: 10.1126/science.aaf0941

Cortês, M., Kauffman, S., Liddle, A., and Smolin, L. (2022). Biocosmology: Biology from a cosmological perspective. arXiv [Preprint]. doi: 10.48550/arXiv.2204.09379

Damasio, A. (2005). *Descartes' Error: Emotion, Reason, and the Human Brain*. New York: Penguin Publishing Group.
Darwin, C. (1895). *Origin of Species*. John Murray.
Deutsch, D. (1997). *The Fabric of Reality*. Allen Lane.
Deutsch, D. (2011). *The Beginning of Infinity*. New York: Penguin Books.
Devereaux, A., Koppl, R., Kauffman, S., and Roli, A. (2022). An incompleteness result regarding within-system modeling. *SSRN*, 1-15. doi: 10.2139/ssrn.3968077
Doeller, C. F., Barry, C., and Burgess, N. (2010). Evidence for grid cells in a human memory network. *Nature*, 463(7281), 657-661. doi: 10.1038/nature08704
Egbert, M., and X. Barandiaran. (2014). Modeling habits as self-sustaining patterns of sensorimotor behavior. *Front. Human Neuroscience,* 8, 1-15. doi: 10.3389/fnhum.2014.00590
Etherington, L. (2020). Melanie Klein and object relations theory. https://www.simplypsychology.org/Melanie-Klein.html#:~:text=Klein%20believed%20that%20ego%20formation,part%2Dobject%20'breast' [Accessed October 16, 2022].
Fichte, J. G. (2014). An attempt at a new presentation of the Wissenschaftslehre. *Sententiae*, 31(2), 97-107. doi: 10.22240/sent31.02.097
Freud, S. (1915). *The Interpretation of Dreams* (translated by A.A. Brill).
Freud, S. (1920). *Beyond the Pleasure Principle*.
Freud, S. (1926). *Inhibitions, Symptoms and Anxiety*.
Futura, A. (2012). One thing is certain: Heisenberg's uncertainty principle is not dead. *Scientific American*. https://www.scientificamerican.com/article/heisenbergs-uncertainty-principle-is-not-dead/ [Accessed February 13, 2023].
Gabriel, M. (2011). *Transcendental Ontology: Essays in German Idealism*. New York: Continuum.
Grouchy, P., D'Eleuterio, G. M., Christiansen, M. H., and Lipson, H. (2016). On the evolutionary origin of symbolic communication. *Sci. Rep.* 6, 1-9. doi: 10.1038/srep34615
Guryanova, Y., Popescu, S., Short, A. J. et al. (2016). Thermodynamics of quantum systems with multiple conserved quantities. *Nature Communications*, 7(12049), 1-9. doi: 10.1038/ncomms12049
Hafting, T., Fyhn, M., Molden, S., Moser, M-B., and Moser, E. I. (2005). Microstructure of a spatial map in the entorhinal cortex. *Nature*, 436, 801-806. doi: 10.1038/nature03721
Hawkins, J., and Ahmad, S. (2016). Why neurons have thousands of synapses, a theory of sequence memory in the neocortex. *Front. Neural Circuits*, 10(23), 1-13. doi: 10.3389/fncir.2016.00023
Hawkins, J., Ahmad, S., and Cui, Y. (2017). A theory of how columns in the neocortex enable learning the structure of the world. *Front. Neural Circuits*, 11, 1-18. doi: 10.3389/fncir.2017.00081
Hawkins, J., Lewis, M., Klukas, M., Purdy, S., and Ahmad, S. (2018). A framework for intelligence and cortical function based on grid cells in the neocortex. *Front. Neural Circuits*, 12, 1-14. doi: 10.3389/fncir.2018.00121
Hawkins, J., and Dawkins, R. (2021). *A Thousand Brains: A New Theory of Intelligence*. New York: Basic Books.
Hegel, G. W. F. (1969). *Science of Logic* (translated by A. V. Miller). Allen & Unwin.
Hegel, G. W. F. (1977). *Phenomenology of Spirit* (translated by A. V. Miller). Oxford: Oxford University Press.
Hilbert, D. (1983). "On the infinite," in*Philosophy of Mathematics: Selected Readings*, eds. P. Benacerraf and H. Putnam (Harvard: Cambridge University Press), 183-201.
Hossenfelder, S. (2018). *Lost in Math: How Beauty Leads Physics Astray*. New York: Basic Books.
Hossenfelder, S. (2022). *Existential Physics: A Scientist's Guide to Life's Biggest Questions*. Great Britain: Atlantic Books.

Juarrero, A. (1991). Fail- safe versus safe- fail: Suggestions toward an evolutionary model of justice. *Texas Law Review*, 69, 1745-1777.
Juarrero, A. (2023). *Context Changes Everything: How Constraints Create Coherence*. Cambridge: MIT Press.
Johnston, A. (2008). *Zizek's Ontology: A Transcendental Materialist Theory of Subjectivity*. Northwestern University Press.
Kandel, E. R. (1999). Biology and the future of psychoanalysis: A new intellectual framework for psychiatry revisited. *Am. J. Psychiatry,* 156, 508-509. doi: 10.1176/ajp.156.4.505
Kauffman, S. (2022). Is there a fourth law for non-ergodic systems that do work to construct their expanding phase space? *Entropy* 24(10), 1-12. doi: 10.3390/e24101383
Kleckner, I., Zhang, J., Touroutoglou, A., Chanes, L., Xia, C., Simmons, W. K., Quigley, K. S., Dickerson, B. C., and Feldman Barrett, L. (2017). Evidence for a large-scale brain system supporting allostasis and interoception in humans. *Nat. Hum. Behav.*, 1. doi: 10.1038/s41562-017-0069
Lakoff, G., and Johnson, M. (1980). *Metaphors We Live By.* Chicago: University of Chicago Press.
Lakoff, G. (1996). *Moral Politics: How Liberals and Conservatives Think*. London: University of Chicago Press.
LeDoux, J. E. (1986). *The Emotional Brain*. Plenum.
Lewis, M., Purdy, S., Ahmad, S., and Hawkins, J. (2019). Locations in the neocortex: A theory of sensorimotor object recognition using cortical grid cells. *Front. Neural Circuits*, 13, 1-18. doi: 10.3389/fncir.2019.00022
Livingstone, M. S., Srihasam, K., and Morocz, I. A. (2010). The benefit of symbols: Monkeys show linear, human-like, accuracy when using symbols to represent scalar value. *Anim. Cogn.* 13(5), 711-719. doi: 10.1007/s10071-010-0321-1
Macpherson, C. B. (1962). *The Political Theory of Possessive Individualism*. Oxford: Oxford University Press.
Malabou, C. (2005). *The Future of Hegel: Plasticity, Temporality and Dialectic* (translated by L. During). London: Routledge.
McRae, M. (2023). A first-of-its-kind signal has been detected in the human brain. https://www.sciencealert.com/a-first-of-its-kind-signal-has-been-detected-in-the-human-brain [Accessed April 23, 2023].
Minkel, J. R. (2007). Quantum spookiness spans the Canary Islands. *Scientific American*. https://www.scientificamerican.com/article/entangled-photons-quantum-spookiness/ [Accessed March 24, 2023].
Mountcastle, V. B. (1997). The columnar organization of the neocortex. *Brain*, 120, 701-722. doi: 10.1093/brain/120.4.701
Mountcastle, V. B. (2009). "Vernon B. Mountcastle," in *The History of Neuroscience in Autobiography*, ed. L. R. Squire (Elsevier Academic Press), 342-379.
Moyar, D. (ed.). (2017). *The Oxford Handbook of Hegel*. Oxford: Oxford University Press.
Nagel, T. (1974). What is it like to be a bat? *Philosophical Review*, 83(4), 435-450.
Naidoo, M. (2023a). The open ontology and information society. *Forthcoming in Front. Genet*.
Naidoo, M. (2023b). Hegel among the sciences: logical development. *Forthcoming in Humanities and Social Sciences Communications*.
Naidoo, M. (2023c). Batman & the Joker: Hegel (em)bodying Kant. *Forthcoming in American Psychological Association: Journal of Theoretical and Philosophical Psychology*.
Nauta, W. J. H. (1971). The problem of the frontal lobe: A reinterpretation. *Journal of Psychiatric Research*, 8(3-4), 167-187. doi: 10.1016/B978-0-08-017007-7.50007-0
Nietzsche, F. (1896). *On Truth and Lies in a Nonmoral Sense*.

Nowak, M. A., Boerlijst, M. C., Cooke, J., and Maynard Smith, J. (1997). Evolution of genetic redundancy. *Nature* 388, 167-171. doi: 10.1038/40618

Platkiewicz, J., and Brette, R. (2010). A threshold equation for action potential initiation. *PLoS Comput. Biol.* 6(7), 1-16. doi: 10.1371/journal.pcbi.1000850

Ptak, R., Doganci, N., and Bourgeois, A. (2021). From action to cognition: Neural reuse, network theory and the emergence of higher cognitive functions. *Brain Sci.* 11(12), 1-20. doi: 10.3390/brainsci11121652

Rajmohan, V., and Mohandas, E. (2007). The limbic system. *Indian J. Psychiatry* 49(2), 132-139. doi: 10.4103/0019-5545.33264

Rovelli, C. (2018). *The Order of Time*. New York: Riverhead.

Rowlands, P. (2007). *Zero to Infinity: The Foundations of Physics*. Singapore: World Scientific Publishing Company.

Saper, C. B., and Lowell, B. B. (2014). The hypothalamus. *Current Biology,* 24(23), R1111-R1116. doi: 10.1016/j.cub.2014.10.023

Sapolsky, R. M. (2017). *Behave: The Biology of Humans at our Best and Worst*. New York: Penguin Books.

Sbardolini, G. (2022). On the origin of negation. *Erkenntnis* 1-20. doi: 10.1007/s10670-022-00627-6

ScienceDirect. (n.d.). Neuronal depolarization. https://www.sciencedirect.com/topics/biochemistry-genetics-and-molecular-biology/neuronal-depolarization#:~:text=Neuronal%20depolarization%20depends%20on%20the,drug%20is%20rapid%20and%20brief [Accessed March 5, 2023].

ScienceDirect. (n.d.). Threshold potential. https://www.sciencedirect.com/topics/medicine-and-dentistry/threshold-potential [Accessed April 17, 2023].

Setterberg, S. (2017). The development of the mind: A three month old infant. *Shanghai Arch Psychiatry*, 29(1), 51-54. doi: 10.11919/j.issn.1002-0829.216039

Smolin, L. (2013). *Time Reborn: From the Crisis in Physics to the Future of the Universe*. New York: Houghton Mifflin Harcourt.

Solms, M. (2017). What is 'the unconscious,' and where is it located in the brain? A neuropsychoanalytic perspective. *Ann. N. Y. Acad. Sci.* 1406, 90-97. doi: 10.1111/nyas.13437

Strachey, J. (1964). *The Standard Edition of the Complete Psychological Works of Sigmund Freud: Volume XXII (1932-1936): New Introductory Lectures on Psycho-Analys is and Other Works.* Hogarth Press.

Taleb, N. N. (2004). *Fooled by Randomness: The Hidden Role of Chance in Life and in the Markets*. New York: Penguin Books.

Uffink, J. (2016). The uncertainty principle. *Stanford Encyclopedia of Philosophy*. https://plato.stanford.edu/entries/qt-uncertainty/ [Accessed February 17, 2023].

Waxenbaum, J. A., Reddy, V., and Varacallo, M. (2022). *Anatomy, Autonomic Nervous System*. Treasure Island: StatPearls.

Wikipedia. (2023). Everything everywhere all at once. https://en.wikipedia.org/wiki/Everything_Everywhere_All_at_Once [Accessed April 24, 2023].

Williamson, R. (2016). Hegel among the quantum physicists. *International Journal of Žižek Studies* 3(1), 1-15.

Wimsatt, W. C. (2001). "Generative entrenchment and the developmental systems approach to evolutionary process," in *Cycles of Contingency: Developmental Systems and Evolution*, eds. S. Oyama, R. Gray, and P. Griffiths (Cambridge: MIT Press).

Wimsatt, W. C. (2007). Reengineering philosophy for limited beings. Cambridge: Harvard University Press.

Winsor, M. P. (2023). Darwin's dark matter: Utter extinction. *Annals of Science* 1-33. doi: 10.1080/00033790.2023.2194889

Žižek, S. (1989). *The Sublime Object of Ideology*. London: Verso.
Žižek, S. (1996). *The Indivisible Remainder: An Essay on Schelling and Related Matters*. London: Verso.
Žižek, S. (2012). *Less Than Nothing: Hegel and the Shadow of Dialectical Materialism*. London: Verso.
